# Supplementary material for: Complex interventions to implement a diabetic retinopathy care pathway in the public health system in Kerala: the Nayanamritham study protocol
Source: BMJ Open. 2021 Jun 28;11(6):e040577. doi: 10.1136/bmjopen-2020-040577 (PMC8240569; doi:10.1136/bmjopen-2020-040577)
Supplement: Author's manuscript [file bmjopen-2020-040577.draft_revisions.pdf]

BMJ Open

BMJ Open is committed to open peer review. As part of this commitment we make the peer review history of every article we publish publicly available.

When an article is published we post the peer reviewers' comments and the authors' responses online. We also post the versions of the paper that were used during peer review. These are the versions that the peer review comments apply to.

The versions of the paper that follow are the versions that were submitted during the peer review process. They are not the versions of record or the final published versions. They should not be cited or distributed as the published version of this manuscript.

BMJ Open is an open access journal and the full, final, typeset and author-corrected version of record of the manuscript is available on our site with no access controls, subscription charges or pay-per-view fees (http://bmjopen.bmj.com).

If you have any questions on BMJ Open's open peer review process please email info.bmjopen@bmj.com

BMJ Open

Complex interventions to implement a diabetic retinopathy care pathway in the public health system in Kerala: an effectiveness-implementation protocol of the Nayanamritham project.

Journal:	*BMJ Open*
Manuscript ID	bmjopen-2020-040577
Article Type:	Protocol
Date Submitted by the Author:	18-May-2020
Complete List of Authors:	Sivaprasad, Sobha; Consultant Ophthalmologist, NIHR Moorfields Biomedical Research<br>Netuveli, Gopalakrishnan; University of East London<br>Wittenberg, Raphael; University of Oxford<br>Khobragade, Rajan; Government of Kerala<br>Sadanandan, Rajeev; Government of Kerala<br>Gopal, Bipin; Directorate of Health Services<br>Premnazir, Lakshmi; Directorate of Health Services<br>Conroy, Dolores; UCL Institute of Ophthalmology<br>Srinath, Jyotsna; University of East London<br>Ramakrishnan, Radha; UCL Institute of Ophthalmology<br>George, Simon; Regional Institute of Ophthalmology<br>Sahasranamam, Vasudeva Iyer ; Regional Institute of Ophthalmology
Keywords:	Diabetic retinopathy < DIABETES & ENDOCRINOLOGY, General diabetes < DIABETES & ENDOCRINOLOGY, Protocols & guidelines < HEALTH SERVICES ADMINISTRATION & MANAGEMENT

SCHOLARONE™
Manuscripts

I, the Submitting Author has the right to grant and does grant on behalf of all authors of the Work (as defined in the below author licence), an exclusive licence and/or a non-exclusive licence for contributions from authors who are: i) UK Crown employees; ii) where BMJ has agreed a CC-BY licence shall apply, and/or iii) in accordance with the terms applicable for US Federal Government officers or employees acting as part of their official duties; on a worldwide, perpetual, irrevocable, royalty-free basis to BMJ Publishing Group Ltd ("BMJ") its licensees and where the relevant Journal is co-owned by BMJ to the co-owners of the Journal, to publish the Work in this journal and any other BMJ products and to exploit all rights, as set out in our licence.

The Submitting Author accepts and understands that any supply made under these terms is made by BMJ to the Submitting Author unless you are acting as an employee on behalf of your employer or a postgraduate student of an affiliated institution which is paying any applicable article publishing charge ("APC") for Open Access articles. Where the Submitting Author wishes to make the Work available on an Open Access basis (and intends to pay the relevant APC), the terms of reuse of such Open Access shall be governed by a Creative Commons licence – details of these licences and which Creative Commons licence will apply to this Work are set out in our licence referred to above.

Other than as permitted in any relevant BMJ Author's Self Archiving Policies, I confirm this Work has not been accepted for publication elsewhere, is not being considered for publication elsewhere and does not duplicate material already published. I confirm all authors consent to publication of this Work and authorise the granting of this licence.

For peer review only

Complex interventions to implement a diabetic retinopathy care pathway in the public health system in Kerala: an effectiveness-implementation protocol of the Nayanamritham project.

Sobha Sivaprasad[1, 2], Gopalakrishnan Netuveli[3], Raphael Wittenberg4, Rajan Khobragade[5], Rajeev Sadanandan[5], Bipin Gopal[6], Lakshmi Premnazir[6], Dolores Conroy[2], Jyotsna Srinath[3], Radha Ramakrishnan[2], Simon George[7], Vasudeva Iyer Sahasranamam[7] for the Nayanamritham Project Collaborators.

1. NIHR Biomedical Research Centre, Moorfields Eye Hospital NHS Foundation Trust, London, UK
2. UCL Institute of Ophthalmology, London, UK
3. University of East London, UK
4. University of Oxford, Oxford, UK
5. Government of Kerala, India
6. Directorate of Health Services, Kerala, India
7. Regional Institute of Ophthalmology, Thiruvananthapuram, Kerala, India

For peer review only

Word count: 3696

Figures: 1

References: 17

Abstract: 291

Corresponding author

Professor Sobha Sivaprasad
NIHR Moorfields Biomedical Research Centre
Moorfields Eye Hospital NHS Foundation Trust,
162 City Road,
London EC1V 2PD, UK
Tel: 020 7253 3411
Email: senswathi@aol.com

Keywords: diabetic retinopathy, implementation study, Kerala, laser photocoagulation, diabetes, screening, visual impairment, cost-effectiveness.

Trial Registration Number: ISRCTN28942696

For peer review only - http://bmjopen.bmj.com/site/about/guidelines.xhtml

Abstract

Introduction: Diabetic retinopathy (DR) is a common cause of visual impairment in working-age people. In high income countries, established DR care pathways are effective but complex and so direct translation of these pathways to the public health system in most low and middle-income countries is challenging. This type-2 hybrid effectiveness-implementation study is a United Kingdom (UK) – Government of Kerala collaboration aimed to roll out co-designed interventions adapted to local needs and resources to initiate a DR care pathway in the public health system as a pilot project in Thiruvananthapuram district in Kerala.

Methods and analysis: Currently, there is no systematic DR screening programme in Kerala. In this study, retinal screening will be initiated for people with diabetes registered within non-communicable disease (NCDs) registers in 16 family health centres (FHCs) in the Thiruvananthapuram district. Patients with referable DR or ungradable images will be referred to three secondary care hospitals and a tertiary eye care centre. The interventions will include integrating DR screening as an episode in the electronic health records system (Ehealth), multi-level training of doctors, nurses, optometrists, data operators and community health workers, increasing public awareness through health workers, provision of retinal cameras and lasers for treatment, development of protocols for the DR care pathway and establishing a retinal image grading centre at a tertiary institute. Effectiveness outcomes will include numbers screened and treated in 12 months, presenting visual acuity of those who require treatment in the study period. Implementation outcomes include acceptability, adoption, appropriateness, feasibility, fidelity, coverage, costs and sustainability.

Ethics and Dissemination: The study was approved by Indian Medical Research Council (2018-0551). Study findings will be disseminated through scientific publications and presentations. Outputs from the study may also be adopted in other public health systems in India and globally.

For peer review only - http://bmjopen.bmj.com/site/about/guidelines.xhtml

ARTICLE SUMMARY

Strengths and limitations of this study

- This study will report the steps required to set up a DR care pathway adapted to local needs within a resource constrained environment that may be more translatable to other low and middle income countries compared to replicating established programmes in high income countries.
- The prevalence of DR in this study will provide baseline data based on capture of retinal images using low cost portable camera and telemedicine in Kerala where data on DR is currently non-existent in the public health system in Kerala.
- As there is no previous data on the prevalence of DR in the public system, the comparator will be a situational analysis of patients presenting to the tertiary centre in the same district and data obtained from a house-to-house survey in a neighbouring district.

For peer review only

For peer review only - http://bmjopen.bmj.com/site/about/guidelines.xhtml

INTRODUCTION

Background

This triad of diabetes, blindness and poverty is an urgent problem that needs an agile response in the Development Assistance Committee-listed countries as these countries are home to 80% of people with diabetes.[1] Diabetic retinopathy (DR) is one of the most common complication of diabetes.[2] Sight-threatening diabetic retinopathy (STDR) is a common cause of blindness in working-age people and unless this condition is managed early, it has considerable impact on the quality of life and productivity of the person and their family, as well as substantial financial costs to health systems. In its early stages, STDR may be asymptomatic. Therefore, DR screening programs are essential to identify STDR to enable timely treatment.[3] In DR screening programs in high income countries, people with diabetes are systematically screened using office-based retinal cameras and the retinal images are graded according to severity of DR by dedicated accredited screeners and graders. Patients with STDR are identified and referred to ophthalmologists for timely treatment with laser and/or intravitreal injections of anti-vascular endothelial growth factor.[3] In order to successfully reduce the risk of blindness due to DR in low-middle income countries (LMICs) without a well-developed primary care infrastructure, the pathway needs to begin with systematically screening for diabetes, educating the public and healthcare professionals on early detection and timely treatment of STDR and the need to optimally treat the risk factors for DR such as hyperglycaemia, hypertension and hyperlipidemia.[4] Unlike other complications of diabetes, DR screening and treatment also require technical expertise adding to the complexity of the required interventions.

Diabetes in Kerala

Kerala is the most advanced state in India in terms of literacy, health, social uplift and demographic transition. This economic success is marred by the fact that diabetes is highly prevalent in Kerala. Recent report from Kerala suggest that one in five of the Kerala adult population may have diabetes.[5] The Government of Kerala launched the Aardram Mission in 2017 to transform and gear up the State’s public health care system to achieve the Sustainable Development Goals (SDGs) in phases with short term goals

For peer review only - http://bmjopen.bmj.com/site/about/guidelines.xhtml

on building infrastructure and quality care services. The overarching objectives of the Aardram Mission included providing equitable, affordable and quality care to citizens from all socio-economic strata; strengthening the public care system by decentralising healthcare from the secondary and tertiary level to primary care led services and initiating preventive medicine to address the impact of NCDs, especially hypertension and diabetes. Primary care centres have been converted to family health centres (FHCs) with three doctors and four nurses in each FHC assigned to provide individual care plans to the allocated population and records tracked through a recently established Electronic Health Record (EHealth). These recent developments delivered through the Aardram Mission are timely contributions that will aid the implementation of a DR care pathway.

The challenge and the rationale for complex interventions

The Government of Kerala is pressing forward to achieve universal health coverage and address the SDG goals on poverty (SDG 1), health access (SDG 3), education (SDG 4) and gender equality (SDG 5). [6,7] Therefore, there is an urgent need to tackle the complications of diabetes. Systematic DR screening has been shown to be effective in reducing blindness.[3] However, in high income countries, DR screening is achieved by dedicated services due to the technicalities and expertise required in DR screening and evaluation.[8-11] Introducing an isolated DR programme will not be sufficient to address the challenge in LMICs as these countries need to initiate holistic screening service for diabetes and all its complications simultaneously and embed the DR screening within the primary care. Another challenge is that mydriasis (pupil dilatation) is compulsory in some screening programmes but not in others and this process requires local approval.[3,10] Other issues faced by LMIC are that the number of undiagnosed diabetes cases is high, the non-availability of a state-wide diabetes register with recall facilities, the lack of resources for standardised non-portable retinal cameras, the staff in the primary care are not trained to screen for DR, and the number of people with diabetes far outnumbers the capacity of the services provided by the secondary care hospitals. The research capacity and capability are in its infancy in LMICs and implementing change in a busy environment when demands on staff are great and resources are

For peer review only - http://bmjopen.bmj.com/site/about/guidelines.xhtml

limited is challenging. More importantly, cataract remains the main cause of visual impairment and retinal screening using retinal cameras through hazy media due to cataract may not be possible resulting in more ungradable images than observed in screening programmes in developed countries. Therefore, each aspect of the DR screening and care pathway has to be adapted to local needs and resources.

Rationale for the Implementation Strategy

An effectiveness evaluation alone would not provide sufficient information on scalability and sustainability to the whole Kerala State. Therefore, we need to consider implementation outcomes and will include acceptability, adoption, appropriateness, feasibility, fidelity -and sustainability.

Aims and Objectives

This study is facilitated by a UK-Kerala partnership funded by Global Challenges Research Fund, UK Research and Innovation and the Government of Kerala. We aim to introduce and evaluate complex interventions to initiate DR screening and treatment in the public health system in Kerala by developing a care pathway that spans primary, secondary and tertiary care in a pilot study in Thiruvananthapuram. The proposed study will examine (1) the clinical and cost effectiveness of the DR care pathway and (2) the implementation outcomes of the complex new interventions in a Thiruvananthapuram district-wide pilot. These interventions include building research capacity and capability in Kerala and necessitating input and changes at multiple organisational levels.

METHODS

Design: We chose a type 2- hybrid effectiveness-implementation study to evaluate the effectiveness of the complex intervention. Effectiveness will be evaluated by uptake of DR screening and numbers treated for STDR. In addition, the visual acuity at presentation will be a key indicator of the effectiveness of the programme as we expect that patients referred for STDR from the screening programme will have better visual acuity than patients who self-refer to the tertiary care centre. Data on visual acuity of self-referred patients will be collected as a situational analysis and compared to the visual acuity of patients referred from screening.

For peer review only - http://bmjopen.bmj.com/site/about/guidelines.xhtml

Target Population

People with diabetes registered in the NCD register at 16 FHCs in Thiruvananthapuram district will be invited to be screened for complications of diabetes including screening for DR. As the NCD register is likely to show an increase in newly diagnosed diabetes as a result of training the accredited social health activists (ASHAs), we will evaluate the effectiveness of the complex interventions of patients recruited at each of the 16 FHCs for this study while the implementation of the pathway will take into account all patients in the NCD register that require DR screening on a regular basis.

Target sites for implementation of DR screening and treatment in Thiruvananthapuram will include 16 FHCs for DR screening which represents the primary care where patients are screened for all complications of diabetes. The referred patients will be managed in three secondary care hospitals and the tertiary care centre, the Regional Institute of Ophthalmology (RIO) for specialist management of DR.

Description of the standard of care

Currently, patients with diabetes are not systematically screened for DR in the public health system. Most patients present to the tertiary centre voluntarily either because of increased awareness of complications of diabetes or due to visual impairment. Therefore, the current intervention will be captured as the number of patients presenting to the tertiary centre for an eye consultation for DR over a period of time as there is no baseline data in the primary and secondary care. Baseline survey of the patients presenting at the FHCs may also provide information on the prevalence of current DR screening in people registered in the NCD register.

Planned Diabetic Retinopathy Pathway for Implementation

The study will introduce pragmatic interventions to initiate a DR care pathway (figure 1) spanning primary, secondary and tertiary care to ensure:

1. DR screening of patients with diabetes registered in the NCD register at FHCs (primary care).
2. Prompt referral for timely treatment of STDR to secondary care and tertiary centres depending on the severity of the DR.
3. Treatment of patients with sight threatening DR (at secondary and tertiary care).

Complex Interventions

For peer review only - http://bmjopen.bmj.com/site/about/guidelines.xhtml

1. Increasing numbers of people with diabetes are being registered in the NCD registers at the FHCs: The public health NCD programme has already started a campaign to raise public health awareness of diabetes and hypertension, the two major risk factors for DR. ASHAs link communities with primary care and have proven to be very effective in improving basic health care services especially for poorer people. One intervention in this study is to train the ASHAs to increase their knowledge of diabetes and DR as a complication of diabetes and this increased knowledge may in turn reflect in more people being encouraged to visit the FHCs for screening for diabetes. Currently, there are 51,000 people registered in the NCD register across 16 FHCs in Thiruvananthapuram.

2. People registered in the NCD register attending the FHCs for their diabetes care will be invited for DR screening. Nurses and doctors in the FHCs will receive training on DR, pupil dilatation and capture of retinal images using low cost smartphone retinal cameras. Recent reports from India suggest good diagnostic accuracy of images captured using these retinal cameras.[14]

3. The retinal images will then be linked to the patient's electronic health record (EHealth) and transferred through a secure cloud to RIO, the tertiary centre, where the images will be graded for their DR status and the results fed back to the FHCs. This telemedicine approach has been validated in various private or public-private ventures in India and other countries with encouraging results. It is anticipated that these cameras will be further developed to incorporate AI-based automatic retinal image grading. Capacity and capability will need to be built at the tertiary care centre to ensure a quality assured reporting system is developed and image grading by trained optometrists and ophthalmologists. If the retinal images are not gradable or if the patient has STDR, the patient will be referred to a DH for dilated pupil examination and/or laser treatment for STDR. If DR requires any other interventions, the patients will be referred to RIO.

5. Ophthalmologists in DH will be trained to perform laser treatment.

6. Data entry operators will be trained to collect study-specific research data. A project manager will oversee the whole project.

For peer review only - http://bmjopen.bmj.com/site/about/guidelines.xhtml

Therefore, this study will evaluate a number of interdependent interventions with the aim to optimise the DR care pathway that can be rolled out to the whole state to ensure sustainability and generalizability.

Outcomes

Pre-specified effectiveness outcomes numbers screened per FHC; prevalence of DR and STDR in different age groups to determine appropriate age to screen for complications of diabetes including DR; completeness of the NCD register in each FHC and the increase in registration post-training of the ASHAs; prevalence of risk factors for other complications of diabetes including those with nephropathy and cardiovascular diseases; treatment outcomes of people with STDR; cost of screening. Implementation outcomes include adoption by health professionals at various levels of care with minor changes to suit service needs; acceptability will be measured by patient satisfaction, acceptable by clinicians, health professionals, health department and policy makers; appropriateness and feasibility of the programme will be evaluated by the increase in service implementation and utilisation; barriers and facilitators; fidelity will be evaluated by adherence to the co-developed DR care pathway and efficiencies recommended in structures, resources and processes; sustainability is the maintenance of the programme after the end of the study and up-scaling to other regions in Kerala; scope of implementing a diabetes register that could be linked to eHealth.

Economic Evaluation

The key outcome is the number of cases of severe visual impairment or blindness due to STDR averted as a result of the new care pathway and estimated QALY gains arising from cases averted. We will estimate the costs of the screening programme in total and per person. We will include the costs of staff training and cameras (suitably annuitized), staff costs and travel costs of patients. We will rely on EQ5D utility value findings from past studies, in particular Polack et al 2015.[12] The objectives of the health economic modelling are to estimate the costs, cost consequences and cost-effectiveness of the new care pathway. We will model the comparator as no screening at primary and secondary care and assume that the effect of no screening is as reported in historic studies that reported the visual outcome of DR if left untreated.

For peer review only - http://bmjopen.bmj.com/site/about/guidelines.xhtml

Process Evaluation

This mixed methods process evaluation will be completed at the beginning and end of the project. We will study the five domains within the Medical Research Council guidance of process evaluation of complex interventions, that is, reach, efficacy, adoption, implementation, and maintenance (RE-ALM framework) at individual and organisational levels.[13, 14]

Sample size

We have chosen the proportion of patients in the NCD register with diabetes screened for DR as our primary outcome variable.

Justification of the choice of primary outcome: This variable captures the effectiveness of our intervention as well as the fidelity of implementation. Other facts that informed our choice of the primary outcome is that we expect a short implementation time of 7 to 9 months during which other outcomes such as number of patients treated may not be a feasible option. Therefore, we have estimated the sample size based on this primary outcome.

Our calculations are complicated by the paucity of information about the size of NCD register and the baseline proportion of patients that attend the tertiary centre for screening. The prevalence of diabetes is between 10-16% in Thiruvananthapuram of which 8% are estimated to have DR and 3% to have STDR but about 20% may have to be referred.[15] Assuming a finite population of 40,000 patients with diabetes, a simple random sample of 377 patients will be needed. However, we expect large design effects due to clustering patients within FHCs. There is not enough data to calculate the value of this design effect and therefore we assume it to be 3 to give a final sample size of 1,131, which is equivalent to assuming a within-FHC intracluster correlation coefficient of approximately 0.03 (n=16 FHCs) with negligible residual clustering by the intermediate cluster level of ASHA worker, where mean cluster size is smaller.

As described by Becker et al in mammography screening[16], we expect sources of bias in the implementation programme that will likely influence the sample size calculation.

For peer review only

Unscreened DR cases that already exist in the community may contribute to an over-estimation of the effect of screening. We expect lead-time bias because of our short implementation period and so some DR in the community may be missed. A comparison of numbers with DR in FHC with longer implementation period with those of shorter period may adjust for this bias. We intend to provide a descriptive analysis as well as a temporal comparison using time series analysis.

Statistical Analyses

We will create a complete case dataset for use in the analyses of effectiveness and assess the potential impact of missing data using sensitivity analysis incorporating a range of optimistic and pessimistic scenarios for the impact of missing data. We will model two outcomes that we hypothesised would be influenced by the intervention: (1) uptake of DR screening (primary outcome) and (2) numbers of patients referred for STDR as a result of screening. We will use bivariate logistic regression models to assess crude associations between sociodemographic factors and other clinical data with DR and STDR and use fractional polynomials to account for the form of continuous covariates to predict uptake of DR screening. Factors associated with a $P<0.25$ will be candidates for inclusion in the multivariable logistic regression models. We will test interactions amongst identified main effects to capture improvement in model prediction assessed by reduced residual variance-based statistics. In our final adjusted model, we will consider associations with a $P<.05$ as statistically significant. Model discrimination and calibration will be assessed in order to improve transparency of the validity of the interpretation of results.

Data collection

Quantitative data

Effectiveness outcomes will originate from the study data collected during the study period and the NCD register.

Qualitative data

These will include data from survey for health professionals, observational data and key

For peer review only - http://bmjopen.bmj.com/site/about/guidelines.xhtml

informant interviews and focus groups and patient interviews. In addition, to the voice-recording of the interviews and focus groups, interviewers will write field notes to describe the interview situation. Field notes and the interview and focus group content will provide the basis for the data analysis, which will be based on a descriptive phenomenological approach without data or opinion interpretation and will include transcription, condensation, coding and categorisation using qualitative analysis tools.

Ethics and dissemination

The study complies with the guidelines of the Declaration of Helsinki. Results of this research are expected to be disseminated through stakeholder reports and via scientific forums, specifically peer-reviewed publications and conference presentations.

Discussion

At the conclusion of this study, we hope to assess the feasibility, effectiveness and implementation challenges of a complex DR care pathway integrating care at primary, secondary and tertiary care, covering a proportion of the diabetic population in Thiruvananthapuram. This pilot data will be useful for Kerala and perhaps India to plan to achieve the SDGs. The study will set the scene for a policy for State-wide Screening and Treatment Pathway for Diabetes Complications with clinical impact on the patient, impact on the community, impact on staff workload and economic impact on family and public finances and integrating it to primary care practice.[17] Early identification of STDR and other complications due to this holistic approach and timely treatment, will have positive impact on rates of blindness, chronic kidney disease, cardiovascular complications and thereby improve health, reduce multi-morbidity and mortality.

This protocol outlines both effectiveness and implementation components[18, 19] to evaluate whether a DR care pathway that spans primary, secondary and tertiary care can be implemented in everyday practice in the public health system. This work is timely given the increasing numbers of people with diabetes and pressure on finances available for healthcare necessitating, task shifting will enable better coverage of the population. Although it is a DR care pathway, we will collect data on other complications of diabetes to address the wider needs of this group of patients. The study will also

For peer review only - http://bmjopen.bmj.com/site/about/guidelines.xhtml

examine the organisation functions such as structure, resources and processes that would contribute to better outcomes. We will also examine whether this additional task is feasible given the current skill levels and workload of FHCs and secondary hospitals. The study will reveal whether current health seeking behaviour of patients will support screening, especially when an invasive procedure is requested when there is no obvious impact on quality of life of the patient. By addressing a key gap in knowledge due to lack of research in this area, we will be able to decipher the barriers and facilitators that influence the successful implementation of such a programme in the public system in India. The results of this study will inform the adoption of this pathway in other areas in India and globally.

There are limitations, however, to the study design. We do not have baseline data on DR screening in the study location as these screening programmes are non-existent. For this reason, we are examining the effectiveness of the pathway in terms of presenting visual acuity for referable cases as 'proximal' outcomes. In addition, when there is no concurrent control group, causal inferences are difficult to make and may lead to both measured and unmeasured biases. When examining implementation, logistical issues, variations in health care facilities, socio-economic variation, and quality of health care personnel may likely affect our study. However, the study design avoids the ethical challenges of having a control group with no DR screening. Therefore, the study design is by necessity non-randomised and observational and will rely on newly trained staff members to collect data, which is likely to differ in completeness between the 16 FHCs. We expect an increase in referral rates after implementation of the intervention due to better public awareness, increasing knowledge of ASHA and improved case ascertainment, at least in some FHCs. We will also examine the impact of cataract in the community and this has not been studied before in any other DR pathway. We have tried to minimise these risks by the use of robust statistical techniques and the triangulation of various data sources to elicit a greater understanding of how the programme will lead to better health outcomes that will include identification of cataract.

For peer review only - http://bmjopen.bmj.com/site/about/guidelines.xhtml

This study has many strengths: Our study has quantitative data backed by qualitative data to strengthen our findings and enable generalisation of our findings. Secondly, we will use logic models and process mining to illustrate the implementation pathway through implementation theory. Third, we will use robust statistical methods to reduce bias including selection bias and other confounders. Finally, the access to and collaboration with the UK is a key strength of the study, as it facilitates the co-development of the interventions from the outset. We expect the findings of this evaluation to be of interest to a wide audience given the growing need to demonstrate effectiveness of complex interventions in low resource settings. Findings on the mechanisms and contexts that optimise the implementation of this complex multi-faceted intervention will be useful to those developing and implementing these programmes in other health systems. The health economic model may highlight the health expenditure required at individual, family and Kerala State level for forecasting and planning health budgets.

Perhaps the most important aspect of the chosen evaluation is that it is built within a simultaneous developing public health strategy on population-based screening of diabetes and hypertension and a recently introduced EHealth. Conducting research within this system strengthens data systems and quality for non-research purposes.

Despite the limitations, this study holds promise for providing high-quality data and detailed implementation information on a complex intervention in a resources-limited setting. We hope to contribute to the literature on the implementation and effectiveness of DR screening and treatment in the public sector in LMICs.

References

1. International Diabetes Federation. *IDF Diabetes Atlas*. 9th edition 2019 https://www.diabetesatlas.org/en/

2. Yau JW, Rogers SL, Kawasaki R, et al. Meta-Analysis for Eye Disease (META-EYE) Study Group. Global prevalence and risk factors of diabetic retinopathy. *Diabetes Care* 2012; 35:556–64.

3. Scanlon PH. The English National Screening Programme for diabetic retinopathy 2003-2016. *Acta Diabetol* 2017; 54(6):515-25

For peer review only - http://bmjopen.bmj.com/site/about/guidelines.xhtml

4. Cavan D, Makaroff L, da Rocha Fernandes J, et al. The Diabetic Retinopathy Barometer Study: Global perspectives on access to and experiences of diabetic retinopathy screening and treatment. *Diabetes Res Clin Pract* 2017; 129:16-24.

5. Menon VU, Kumar KV, Gilchrist A, et al. Prevalence of known and undetected diabetes and associated risk factors in central Kerala--ADEPS. *Diabetes Res Clin Pract* 2006; 74:289–94.

6. Sustainable Development Goals. United Nations. [Last accessed on 2020 May12]. Available from: http://www.un.org/sustainabledevelopment/sustainable-developmentgoals/

7. The WHO STEPwise approach to Surveillance of noncommunicable diseases (STEPS). Noncommunicable Diseases and Mental Health. World Health Organization. 20 Avenue Appia, 1211 Geneva 27, Switzerland.

8. Scanlon PH, Aldington SJ, Leal J, et al. Development of a cost-effectiveness model for optimisation of the screening interval in diabetic retinopathy screening. *Health Technol Assess* 2015;19(74):1-116.

9. Nguyen HV, Tan GS, Tapp RJ, et al. Cost-effectiveness of a National Telemedicine Diabetic Retinopathy Screening Program in Singapore. *Ophthalmology* 2016;123(12):2571-80.

10. Leese GP, Morris AD, Olson J. A national retinal screening programme for diabetes in Scotland. *Diabet Med* 2003;20(12):962-4.

11. Lund SH, Aspelund T, Kirby P, Russell G, Einarsson S, Palsson O, Stefánsson E. Individualised risk assessment for diabetic retinopathy and optimisation of screening intervals: a scientific approach to reducing healthcare costs. Br J Ophthalmol. 2016 May;100(5):683-7.

12. Polack S, Alavi Y, Rachapalle Reddi S, Kulothungan V, Kuper H. Utility values associated with diabetic retinopathy in Chennai, India. Ophthalmic Epidemiol. 2015; 22:1, 20-27.

13. Moore GF, Audrey S, Barker M,et al. Process evaluation of complex interventions: Medical Research Council guidance. *BMJ* 2015;350:h1258.

14. Hasson H. Systematic evaluation of implementation fidelity of complex interventions in health and social care. *Implement Sci* 2010; 5:67. doi: 10.1186/1748-5908-5-67.

15. Rajalakshmi R, Subashini R, Anjana RM, et al. Automated diabetic retinopathy detection in smartphone-based fundus photography using artificial intelligence. *Eye* (Lond) 2018;32(6):1138-1144

16. Becker N, Hakama M, Nyström L. Evaluation of effectiveness of quality-assured mammography screening in Germany: sample size considerations and design options. *Eur J Cancer Prev* 2007;16(3):225-31.

17. Grol R, Grimshaw J. From best evidence to best practice: effective implementation of change in patients' care. *Lancet* 2003;362(9391):1225–30.

18. Boeckmann M, Nohavova I, Dogar O, er al. TB & Tobacco Project Consortium. Protocol for the mixed-methods process and context evaluation of the TB & Tobacco randomised controlled trial in Bangladesh and Pakistan: a hybrid effectiveness-implementation study. *BMJ Open* 2018 ;8(3): e019878.

For peer review only

19. Maru S, Nirola I, Thapa A, et al. An integrated community health worker intervention in rural Nepal: a type 2 hybrid effectiveness-implementation study protocol. *Implement Sci* 2018;13(1):53.

Legend: Figure 1: Proposed Diabetic Retinopathy Care Pathway

Nayanamritham Project Collaborators.

Rajeevan Palpoo
Chitra Raghavan
Alwin Aniyankunju
Anju V.Molly

Acknowledgements: The authors would like to thank all the Study collaborators including field workers, Director of Health Services; Director of Medical Education; Doctors and Nurses at Family Health Centres, Secondary Care and Tertiary Care Centres and Study Participants.
Patient and Public Involvement: Patients or the public will be involved in the conduct of the study, reporting and dissemination plans of our research
Funding statement: This work was supported by Global Challenges Research Fund and UK Research and Innovation through the Medical Research Council grant number MR/P027881/1
Data Sharing Agreement: The technical appendix, statistical code and dataset will be made available on request after obtaining permission from the Government of Kerala.
Competing interests statement: None

Author Contributorship

Sobha Sivaprasad (S.S), Gopalakrishnan Netuveli (G.N), Raphael Wittenberg (R.W), Rajan Khobragade (R.K), Rajeev Sadanandan (R.S), Bipin Gopal (B.G), Lakshmi Premnazir (L.P), Dolores Conroy (D.C), Jyotsna Srinath (J.S), Radha Ramakrishnan (R.R), Simon George (S.G), Vasudeva Iyer Sahasranamam (V.S)

Conceptualisation, S.S, G.N, R.W, R.S, B.G, S.G, V.S; methodology, S.S, G.N, R.R, R.S, B.G, V.S, S.G, D.C, R.R, R.K; acquisition of data, S.S, G.N, R.W, D.C, R.R, J.S, L.P, B.G; writing—original draft preparation, S.S, D.C, G.N, R.W, L.P, R.R; writing—review and editing, S.S, G.N, R.R, R.S, B.G, V.S, S.G, D.C, R.R, R.K, J.S, L.P; funding acquisition, S.S, G.N, R.W, R.S, on behalf of the Collaborators.

For peer review only - http://bmjopen.bmj.com/site/about/guidelines.xhtml

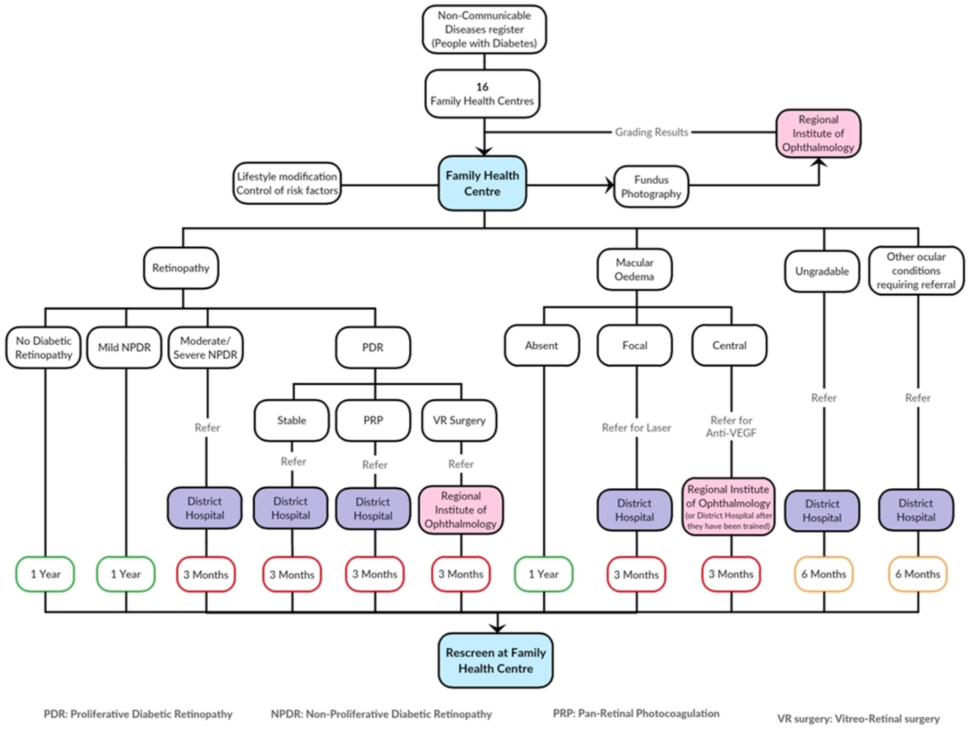

Figure 1: Flow Diagram

186x138mm (300 x 300 DPI)

BMJ Open

Complex interventions to implement a diabetic retinopathy care pathway in the public health system in Kerala: an effectiveness-implementation protocol of the Nayanamritham project.

Journal:	*BMJ Open*
Manuscript ID	bmjopen-2020-040577.R1
Article Type:	Protocol
Date Submitted by the Author:	05-Dec-2020
Complete List of Authors:	Sivaprasad, Sobha; Consultant Ophthalmologist, NIHR Moorfields Biomedical Research<br>Netuveli, Gopalakrishnan; University of East London<br>Wittenberg, Raphael; University of Oxford<br>Khobragade, Rajan; Government of Kerala<br>Sadanandan, Rajeev; Government of Kerala<br>Gopal, Bipin; Directorate of Health Services<br>Premnazir, Lakshmi; Directorate of Health Services<br>Conroy, Dolores; UCL Institute of Ophthalmology<br>Srinath, Jyotsna; University of East London<br>Ramakrishnan, Radha; UCL Institute of Ophthalmology<br>George, Simon; Regional Institute of Ophthalmology<br>Sahasranamam, Vasudeva Iyer ; Regional Institute of Ophthalmology
**Primary Subject Heading**:	Ophthalmology
Secondary Subject Heading:	Public health, Diabetes and endocrinology
Keywords:	Diabetic retinopathy < DIABETES & ENDOCRINOLOGY, General diabetes < DIABETES & ENDOCRINOLOGY, Protocols & guidelines < HEALTH SERVICES ADMINISTRATION & MANAGEMENT

SCHOLARONE™
Manuscripts

I, the Submitting Author has the right to grant and does grant on behalf of all authors of the Work (as defined in the below author licence), an exclusive licence and/or a non-exclusive licence for contributions from authors who are: i) UK Crown employees; ii) where BMJ has agreed a CC-BY licence shall apply, and/or iii) in accordance with the terms applicable for US Federal Government officers or employees acting as part of their official duties; on a worldwide, perpetual, irrevocable, royalty-free basis to BMJ Publishing Group Ltd ("BMJ") its licensees and where the relevant Journal is co-owned by BMJ to the co-owners of the Journal, to publish the Work in this journal and any other BMJ products and to exploit all rights, as set out in our licence.

The Submitting Author accepts and understands that any supply made under these terms is made by BMJ to the Submitting Author unless you are acting as an employee on behalf of your employer or a postgraduate student of an affiliated institution which is paying any applicable article publishing charge ("APC") for Open Access articles. Where the Submitting Author wishes to make the Work available on an Open Access basis (and intends to pay the relevant APC), the terms of reuse of such Open Access shall be governed by a Creative Commons licence – details of these licences and which Creative Commons licence will apply to this Work are set out in our licence referred to above.

Other than as permitted in any relevant BMJ Author's Self Archiving Policies, I confirm this Work has not been accepted for publication elsewhere, is not being considered for publication elsewhere and does not duplicate material already published. I confirm all authors consent to publication of this Work and authorise the granting of this licence.

For peer review only

Complex interventions to implement a diabetic retinopathy care pathway in the public health system in Kerala: an effectiveness-implementation protocol of the Nayanamritham project.

Sobha Sivaprasad[1, 2], Gopalakrishnan Netuveli[3], Raphael Wittenberg4, Rajan Khobragade[5], Rajeev Sadanandan[5], Bipin Gopal[6], Lakshmi Premnazir[6], Dolores Conroy[2], Jyotsna Srinath[3], Radha Ramakrishnan[2], Simon George[7], Vasudeva Iyer Sahasranamam[7] for the Nayanamritham Project Collaborators.

1. NIHR Biomedical Research Centre, Moorfields Eye Hospital NHS Foundation Trust, London, UK
2. UCL Institute of Ophthalmology, London, UK
3. University of East London, UK
4. University of Oxford, Oxford, UK
5. Government of Kerala, India
6. Directorate of Health Services, Kerala, India
7. Regional Institute of Ophthalmology, Thiruvananthapuram, Kerala, India

For peer review only

Word count: 3696

Figures: 1

Table: 1

References: 30

Abstract: 416

Corresponding author

Professor Sobha Sivaprasad
NIHR Moorfields Biomedical Research Centre
Moorfields Eye Hospital NHS Foundation Trust,
162 City Road,
London EC1V 2PD, UK
Tel: 020 7253 3411
Email: senswathi@aol.com

Keywords: diabetic retinopathy, implementation study, Kerala, laser photocoagulation, diabetes, screening, visual impairment, cost-effectiveness.

Trial Registration Number: ISRCTN28942696

Protocol Version: 1.0 dated 13-03-2019

For peer review only - http://bmjopen.bmj.com/site/about/guidelines.xhtml

Abstract

Introduction: This type-2 hybrid effectiveness-implementation study, a United Kingdom (UK) – Government of Kerala collaboration, aims to pilot a new diabetic retinopathy (DR) pathway in the public system in Thiruvananthapuram district in Kerala to understand how it can be scaled up and sustained to the whole state.

Methods and analysis: Currently, there is no systematic DR screening programme in Kerala. Our intervention is a teleophthalmology pathway for people with diabetes registered within non-communicable disease (NCDs) registers in 16 family health centres (FHCs). In this mixed methods study, we will utilise quantitative data from a cross-sectional study and qualitative data from all stakeholders obtained from structured interviews, surveys and focus groups to report the prevalence and risk factors of DR, perform a process evaluation using the RE-AIM framework and test the implementation strategy of this DR pathway. The planned implementation strategy of the pathway will include integrating DR screening as an episode in the electronic health records system (ehealth) in the FHCs; multi-level training of doctors, nurses, optometrists, data operators and community health workers; increasing public awareness through health workers; development and refinement of protocols for the DR care pathway and establishing a teleophthalmology retinal image grading centre at a tertiary institute. Clinical and cost-effectiveness outcomes will be analysed based on prevalence of DR and cost of implementation. Comparisons on prevalence of newly identified DR in a house to house survey completed in a neighbouring district in a parallel study conducted by this group. The outcomes of process evaluation and tests of the implementation strategy will include acceptability, adoption, appropriateness, feasibility, fidelity, coverage, costs and sustainability.

Ethics and Dissemination: The study was approved by Indian Medical Research Council (2018-0551) dated 13/03/2019. Study findings will be disseminated through scientific publications and the report will inform future Kerala state-wide adoption of the pathway.

ARTICLE SUMMARY

- The protocol will test the implementation strategy of a diabetic retinopathy pathway within a resource constrained environment.
- The strength of the study protocol is that it is a type 2- hybrid effectiveness-implementation study using a mixed-methods approach.
- The protocol is designed to do a process evaluation using RE-AIM framework.

For peer review only - http://bmjopen.bmj.com/site/about/guidelines.xhtml

- The protocol also describes the economic evaluation of integrating diabetic retinopathy care pathway within primary care.
- The protocol is limited by the absence of a comparator due to absence of previous data on the prevalence of DR in the public system.

For peer review only

INTRODUCTION

Background

This triad of diabetes, blindness and poverty is an urgent problem that needs an agile response in the Development Assistance Committee-listed countries as these countries are home to 80% of people with diabetes.[1] Diabetic retinopathy (DR) is one of the most common complications of diabetes.[2] Sight-threatening diabetic retinopathy (STDR) is a common cause of blindness in working-age people and unless this condition is managed early, it has considerable impact on the quality of life and productivity of the person and their family, as well as substantial financial costs to health systems.[3] In its early stages, STDR may be asymptomatic. Therefore, DR screening programmes are essential to identify STDR to enable timely treatment.[4] In DR

For peer review only - http://bmjopen.bmj.com/site/about/guidelines.xhtml

screening programmes in high income countries, people with diabetes are systematically screened using office-based retinal cameras and the retinal images are graded according to severity of DR by dedicated accredited screeners and graders. Patients with STDR are identified and referred to ophthalmologists for timely treatment with laser and/or intravitreal injections of anti-vascular endothelial growth factor.[4] In order to successfully reduce the risk of blindness due to DR in low-middle income countries (LMICs), without a well-developed primary care infrastructure, the pathway needs to begin with systematically screening for diabetes, educating the public and healthcare professionals on early detection and timely treatment of STDR and the need to optimally treat the risk factors for DR such as hyperglycaemia, hypertension and hyperlipidemia.[5] Moreover, DR screening and treatment are challenging due to the required technology and technical expertise needed to grade retinal images and deliver costly treatment options, adding to the complexity of the required interventions.

Diabetes in Kerala

Kerala is the most advanced state in India in terms of literacy, health, social uplift and demographic transition. This economic success is marred by the fact that diabetes is highly prevalent in Kerala. Recent report from Kerala suggest that one in five of the Kerala adult population may have diabetes.[6] The Government of Kerala launched the Aardram Mission in 2017 to transform and gear up the State's public health care system to achieve the Sustainable Development Goals (SDGs) in phases with short term goals on building infrastructure and quality care services. The overarching objectives of the Aardram Mission included providing equitable, affordable and quality care to citizens from all socio-economic strata; strengthening the public care system by decentralising healthcare from the secondary and tertiary level to primary care-led services and initiating preventive medicine to address the impact of NCDs, especially hypertension and diabetes. Primary care centres have been converted to family health centres (FHCs) with three doctors and four nurses in each FHC assigned to provide individual care plans to the allocated population and records tracked through a recently established Electronic Health Record (eHealth). These recent developments, delivered through the Aardram Mission, are timely contributions that will aid the implementation of a DR care pathway.

The rationale for a complex DR pathway

The Government of Kerala is pressing forward to achieve universal health coverage and address the SDG goals on poverty (SDG 1), health access (SDG 3), education (SDG 4) and

For peer review only - http://bmjopen.bmj.com/site/about/guidelines.xhtml

gender equality (SDG 5).[7, 8] Therefore, there is an urgent need to tackle the complications of diabetes. Systematic DR screening has been shown to be effective in reducing blindness.[3] However, in high income countries, DR screening is achieved by dedicated services due to the technicalities and expertise required in DR screening and evaluation.[9-12] Introducing an isolated DR programme will not be sufficient to address the challenge in LMICs as these countries need to initiate holistic screening service for diabetes and all its complications simultaneously and embed DR screening within the primary care.

Rationale for an Implementation Strategy

Each aspect of the DR screening and care pathway has to be adapted to local needs and resources, requiring an implementation strategy. For example, mydriasis (pupil dilatation) is compulsory in some screening programmes but not in others and this process requires local approval by stakeholders.[9-12] In Kerala, mydriasis needs to be approved by the health department. Other issues faced by LMIC are that the number of undiagnosed diabetes cases is high,[13] the non-availability of a state-wide diabetes register with recall facilities, the lack of resources for standardised non-portable retinal cameras, the staff in the primary care are not trained to screen for DR, and the number of people with diabetes far outnumbers the capacity of the services provided by the secondary care hospitals.[14-16] Research capacity and capability are in their infancy in LMICs and implementing change in a busy environment when demands on staff are great and resources are limited is challenging.[15, 16] In addition, as cataract is more prevalent in LMIC,[17] the proportion of ungradable retinal images is higher compared to developed countries.[17-19] Therefore, we need to consider a process evaluation of the pathway[20] and test the implementation strategy using both qualitative and quantitative evaluation.[21-22]

Aims and Objectives

This study is facilitated by a UK-Kerala partnership funded by Global Challenges Research Fund (GCRF), UK Research and Innovation and the Government of Kerala. We aim to introduce a DR care pathway that spans primary, secondary and tertiary care in the public health system in Kerala in a pilot study in Thiruvananthapuram. The proposed study will examine (1) the clinical and cost effectiveness of the DR care pathway and (2) the process evaluation of the pathway and (3) test the implementation strategy of the pathway.

METHODS

For peer review only - http://bmjopen.bmj.com/site/about/guidelines.xhtml

Design: We chose a type 2- hybrid effectiveness-implementation study using a mixed-methods approach to evaluate the effectiveness of the clinical interventions, conduct a process evaluation and test the implementation strategies.[23] The study protocol was developed by the Kerala Health Secretary, NCD lead, Health and Medical Education service providers technical and eHealth teams, local authorities and the GCRF- funded co-applicants with a planned set-up, tailoring and training phase for 12 months from 15-03-2018 and a further 12-15 months for recruiting consenting patients to a cross-sectional survey to gather quantitative data. Qualitative data collection from interviews of key informants and focus groups at baseline and end of study and all field notes gathered during the study will be utilised to inform process evaluation using RE-AIM framework and outcomes of testing of the implementation strategies. [20-22]

Target Population

People with diabetes registered in the NCD register at 16 FHCs in Thiruvananthapuram district will be invited to be screened for complications of diabetes including screening for DR. As the NCD register is likely to show an increase in newly diagnosed diabetes as a result of training the accredited social health activists (ASHAs), in diabetes and DR, we will evaluate the effectiveness of the complex interventions of patients recruited at each of the 16 FHCs for this study. The implementation of the pathway will take into account all patients in the NCD register that require DR screening on a regular basis.

Setting:

Target sites for implementation of DR screening and treatment in Thiruvananthapuram will include 16 FHCs for DR screening which represents the primary care where patients are screened for all complications of diabetes. The referred patients will be managed in three secondary care hospitals (district hospitals) and the tertiary care centre, the Regional Institute of Ophthalmology (RIO) for specialist management of DR.

Description of the standard of care

Currently, patients with diabetes are not systematically screened for DR in the public health system. Most patients present to the tertiary centre voluntarily either because of increased awareness of complications of diabetes or due to visual impairment. Therefore, the current standard of care will be captured as the number of patients presenting to the tertiary centre for an eye consultation for DR over a period of time as there is no baseline data in the primary and secondary care. This cross-sectional survey of the patients presenting at the FHCs may also

For peer review only - http://bmjopen.bmj.com/site/about/guidelines.xhtml

provide information on the prevalence of current DR screening in people registered in the NCD register.

Evidence-based clinical intervention:

The new DR care pathway is the intervention and is shown in figure 1. The pathway will span primary, secondary and tertiary care. The components of the pathway are:

1. DR screening of patients with diabetes registered in the NCD register at FHCs (primary care).
2. Prompt referral for timely treatment of STDR to secondary care and tertiary centres depending on the severity of the DR.
3. Treatment of patients with sight threatening DR (at secondary and tertiary care).

Implementation strategies:

The implementation strategies that will be used and tested to support this new DR pathway include:

1. Increasing numbers of people with diabetes are being registered in the NCD registers at the FHCs: The public health NCD programme has already started a campaign to raise public health awareness of diabetes and hypertension, the two major risk factors for DR. The ASHAs, will link communities with primary care to increase registration in the NCD registers.

2. The ASHAs will be trained to increase their knowledge of diabetes and DR as a complication of diabetes and this increased knowledge may in turn be reflected in more people being encouraged to visit the FHCs for screening for diabetes. Currently, there are 51,000 people registered in the NCD register across 16 FHCs in Thiruvananthapuram.

3. Nurses and doctors in the FHCs will receive training on DR, pupil dilatation and capture of retinal images using low cost smartphone retinal cameras.[24, 25] Recent reports from India suggest good diagnostic accuracy of images captured using these retinal cameras.[24] Multiple training sessions are planned at each FHC. The camera manufacturers will be invited to provide hands-on training on capturing good quality retinal images.

4. The retinal images will then be linked to the patient's electronic health record (eHealth) and transferred through a secure cloud to RIO, the tertiary centre, where the images will be graded for their DR status and the results fed back to the FHCs.

For peer review only - http://bmjopen.bmj.com/site/about/guidelines.xhtml

5. This telemedicine approach has been validated in various private or public-private ventures in India and other countries with encouraging results.[26, 27] A validation test of this telemedicine approach will be done as a secondary analysis of the acquired data. It is anticipated that these cameras will be further developed to incorporate AI-based automatic retinal image grading.

6. Capacity and capability will also need to be built at the tertiary care centre to ensure a quality assured reporting system is developed and image grading by trained optometrists and ophthalmologists. If the retinal images are not gradable or if the patient has STDR, the patient will be referred to a district hospital (DH) for dilated pupil examination and/or laser treatment for STDR. If DR requires any other interventions, the patients will be referred to RIO.

7. Ophthalmologists in DH will be trained to perform laser treatment.

8. Data entry operators will be trained to collect study-specific research data.

9. A project manager will oversee the whole project.

10. A continuous monitoring of uptake per FHCs, audit of retinal image quality, readiness of staff at each centre will be done to cyclically change the pathway according to local facilitators and barriers.

Outcomes

Pre-specified outcomes are tabulated in table 1.

Table 1: Summary of study outcomes

	**Outcome**	**Test**
Effectiveness	Uptake of DR care pathway at FHCs, secondary and tertiary care	Strategic output: Adoption of DR screening using the strategy as part of the package of services at FHC by the Government of Kerala based on the clinical and cost-effectiveness data from this study.<br>Quantitative data:<br>– Numbers of people screened for DR in each FHC

For peer review only - http://bmjopen.bmj.com/site/about/guidelines.xhtml

		– Prevalence of DR, STDR and risk factors in people screened.<br>– Proportion of ungradable retinal images and impact of cataract<br>– Risk and complication burden in people screened for DR<br>– Presenting vision of those referred compared to those who self-referred.<br>– Generalisability of the prevalence data compared to house to house survey in neighbouring district.
**Implementation Strategy**[21, 22]		
Training of ASHAs	Increase in NCD registration following start of programme key informant interviews	Quantitative data:<br>– Number of ASHAs trained<br>– Number of training sessions<br>– Attendance rates<br>– Increase in NCD registration following start of programme<br>– Qualitative data from key informant interviews and field notes on how to increase uptake at FHCs and treatment in secondary and tertiary care.
Training of primary care nurses and doctors	Improve numbers of people screened	– Number of doctors and nurses trained<br>– Modules developed<br>– Number of training sessions<br>– Attendance rates<br>– Numbers of certifications of completion of training<br>– Qualitative data from interviews, field notes, meetings
Training of secondary care doctors for laser treatment	Increase in numbers of patients treated for STDR following training and reduce direct referrals to tertiary care	– Number of doctors trained on laser delivery and numbers of laser devices purchased<br>– Number of training sessions<br>– Attendance rates<br>– Understanding solutions for barriers by group meetings and interviews.

For peer review only - http://bmjopen.bmj.com/site/about/guidelines.xhtml

Training and quality assurance of grading of images	Improvement in quality of grading by graders compared to slit-lamp outcome at secondary/ tertiary care (secondary validation test).	– Number of graders trained<br>– Number of training sessions<br>– Attendance rates<br>– Feedback on quality of training manual<br>– Key informant interviews
Data quality of data operators	Quality of data accrued during the study	– Numbers of data operators trained,<br>– Monitoring of data quality by project manager and GCRF co-applicants
Assess readiness of staff	Adaptation of DR pathways	– Evaluate barriers and facilitators at each FHC<br>– Explore solutions through interviews and surveys.
Acceptability of stakeholders	Acceptability will be measured by patient satisfaction, acceptability by clinicians, health professionals, health department and policy makers	– A questionnaire survey for referred patients<br>– Structured interviews of at least 5 nurses and 5 primary care doctors from 16 FHCs, 3-5 ophthalmologists from secondary and tertiary care, 2 data operators and 5 ASHAs and 1 health service administrator to understand challenges for acceptance.
Appropriateness	Increase in service utilisation by patients and provision by staff	Increase in uptake of screening and treatment per month
Adherence	Capture efficiencies in structures, resources and processes and share knowledge amongst stakeholders across the DR pathway.	– Numbers not attending for treatment<br>– interviews of staff and patient survey to understand reason for non-attendance
Sustainability	Policy makers prepare policy on up-scale and sustainability plan for DR pathway, acceptance of guidelines and training material Development of DR integration in eHealth in other districts	Willingness to upscale to other districts in Kerala
Fidelity Evaluation	Degree of the DR pathway implementation fidelity for each activity overall scores by clinic	Quantitative: fidelity evaluation in 16 pilot FHC clinics using a path-process analysis of the screening to identify the common screening pathway used and post screening, and

		post screening, we will do a telephonic follow-up of patients who were referred. The path-process analysis will be based on detailed time-event logs of 70 patients from the 16 FHCs.<br>Data sources: key informant interviews, structured observations and review of facility records.
Assess Context of intervention delivery	Contextual factors that influenced outcomes	Semi-structured interview with participants (patients and health workers); policy makers, diabetes state lead; Diabetologists and Ophthalmologists.<br><br>Site observations
**Economic Evaluation:** Estimate the costs, cost consequences and cost-effectiveness of the new DR care pathway.	The key outcome is the number of cases of severe visual impairment or blindness due to STDR averted as a result of the new care pathway<br>Estimated QALY gains arising from cases averted.<br>Costs of the screening programme in total and per person that will include the costs of staff training and cameras (suitably annuitized), staff costs and travel costs of patients.	Data collected in the study, EQ5D utility value findings from past studies, in particular Polack et al 2015.[28] We will model the comparator as no screening at primary and secondary care and assume that the effect of no screening is as reported in historic studies that reported the visual outcome of DR if left untreated.<br>Data sources: budgets, key informant interviews, direct observations and literature search.<br>Annualise capital costs<br>Adjust all costs for inflation and discount
**Process Evaluation (RE-AIM framework)[20]**	This mixed methods process evaluation will be completed at the beginning and end of the project. We will study the five domains within the Medical Research Council guidance of process evaluation of complex interventions, that is, reach, efficacy, adoption, implementation, and maintenance (RE-AIM	Documentary analysis<br>Qualitative observation from focus group -1<br>Routine monitoring data<br>Interview of at least 5 nurses and 5 primary care doctors from 16 FHCs, 3-5 ophthalmologists from secondary and tertiary care, 2 data operators and 5 ASHAs and 1 health service administrator.

For peer review only - http://bmjopen.bmj.com/site/about/guidelines.xhtml

	framework) at individual and organisational levels.[13, 14]	
Reach	Rate of participation at each FHC and disease burden and its risk factors	Proportion of patients offered screening per FHC. The collected data will also be used to test the performance of the five domains. For example, socio-demographic analysis of the patients screened will inform us about Reach.
Effectiveness	Numbers of patients referred for treatment of STDR or cataract	Numbers referred for further evaluation. Efficiency is defined as optimal use of the service. For this purpose, we will first estimate the time required to process one patient from the path-process analysis. This estimate will be used to determine the number of patients who can be seen on a day. We will then be calculate (1-number of patients screened)/number of patients that can be screened). proportion can be used in a p-type control chart.
Implementation	Intervention adaptations required based on barriers and facilitators	Patients and staff interviews on barriers and facilitators of the programme at FHC, secondary and tertiary level
Adoption and Maintenance	Willingness to initiate the pathway in primary, secondary and tertiary care Sustained effect over time	– Policy to scale-up<br>– Dissemination of findings to stakeholders in other districts<br>– Government investment in the scale-up

Sample size

We have chosen the proportion of patients in the NCD register with diabetes screened for DR as our primary outcome variable.

Justification of the choice of primary outcome: This variable captures the effectiveness of our intervention as well as the fidelity of implementation. Other facts that informed our choice of the primary outcome is that we expect a short implementation time of 7 to 9 months during which

For peer review only - http://bmjopen.bmj.com/site/about/guidelines.xhtml

other outcomes such as number of patients treated may not be a feasible option. Therefore, we have estimated the sample size based on this primary outcome.

Our calculations are complicated by the paucity of information about the size of NCD register and the baseline proportion of patients that attend the tertiary centre for screening. The prevalence of diabetes is between 10-16% in Thiruvananthapuram of which 8% are estimated to have DR and 3% to have STDR but about 20% may have to be referred. Assuming a finite population of 40,000 patients with diabetes, a simple random sample of 377 patients will be needed. However, we expect large design effects due to clustering patients within FHCs. There is not enough data to calculate the value of this design effect and therefore we assume it to be 3 to give a final sample size of 1,131, which is equivalent to assuming a within-FHC intracluster correlation coefficient of approximately 0.03 (n=16 FHCs) with negligible residual clustering by the intermediate cluster level of ASHAs, where mean cluster size is smaller.

As described by Becker et al in mammography screening,[29] we expect sources of bias in the implementation programme that will likely influence the sample size calculation. Unscreened DR cases that already exist in the community may contribute to an over-estimation of the effect of screening. We expect lead-time bias because of our short implementation period and so some DR in the community may be missed. A comparison of numbers with DR in FHC with longer implementation period with those of shorter period may adjust for this bias. We intend to provide a descriptive analysis as well as a temporal comparison using time series analysis.

Statistical Analyses

We will create a complete case dataset of the cross-sectional survey for use in the analyses of effectiveness and assess the potential impact of missing data using sensitivity analysis incorporating a range of optimistic and pessimistic scenarios for the impact of missing data. We will model two outcomes that we hypothesised would be influenced by the intervention: (1) uptake of DR screening (primary outcome) and (2) numbers of patients referred for STDR as a result of screening. We will use bivariate logistic regression models to assess crude associations between sociodemographic factors and other clinical data with DR and STDR and use fractional polynomials to account for the form of continuous covariates to predict uptake of DR screening. Factors associated with a $P<0.25$ will be candidates for inclusion in the multivariable logistic regression models. We will test interactions amongst identified main effects to capture improvement in model prediction assessed by reduced residual variance-based statistics. In our final adjusted model, we will consider associations with a $P<.05$ as statistically

For peer review only - http://bmjopen.bmj.com/site/about/guidelines.xhtml

significant. Model discrimination and calibration will be assessed in order to improve transparency of the validity of the interpretation of results.

Data collection

Quantitative data

Effectiveness outcomes will originate from the study data collected during the study period and the NCD register. Data collected include age, gender, education, occupation and income categories; duration of diabetes, use of insulin, parental history of diabetes, other complications of diabetes including diabetic kidney disease, cardiovascular complications, and diabetic foot. Other study-specific tests done on the day of screening include random blood sugar estimation, urine dipstick test for albuminuria, blood pressure record, body mass index, waist circumference, completion of a lifestyle questionnaire on smoking, diet, physical activity, vision related quality of life and previous history of DR, cataract surgery or any other ocular history.

In the reading centre, the data collection will include the grade of retinopathy in both eyes, presence of cataract and gradability of the retinal images. Data collected on referred patients will include numbers with ungradable images due to cataract, treatment options offered for DR and review appointment.

Qualitative data collection and analysis

These will include data from a structured interview of at least 5 nurses and 5 primary care doctors from 16 FHCs, 3-5 ophthalmologists from secondary and tertiary care, 2 data entry operators and 5 ASHAs and 1 health service administrator. Consent will not be obtained from these health professionals. The interview will be conducted by an independent member from the GCRF team, who is not involved in this study, in the local language within the premise of the healthcare provider. The focus group will consist of groups of patients and staff within one FHC. In addition, to the voice-recording of the interviews and focus group, interviewers will write field notes to describe the interview situation. Field notes, the interviews and focus group content will provide the basis for the data analysis, which will be based on a descriptive phenomenological approach without data or opinion interpretation and will include transcription, condensation, coding and categorisation using qualitative analysis tools. A survey of all referred patients will be done using a structured questionnaire to evaluate their satisfaction and their perception of the barriers and facilitators.

For peer review only - http://bmjopen.bmj.com/site/about/guidelines.xhtml

Monitoring:

Data is coded before the clinical teams extract the data from eHealth for analysis. Data quality is checked by the data operators and monitored by the study project manager. Anonymised data is checked for range checks and data quality at University of East London by the study statistics team. The International Advisory Board has an overview of the conduct of the project and the Executive Group consisting of the grant holders monitor the study conduct.

Ethics and dissemination

The study was approved by Indian Medical Research Council (2018-0551).The study complies with the guidelines of the Declaration of Helsinki. Results of this research are expected to be disseminated through stakeholder reports and via scientific forums, specifically peer-reviewed publications and conference presentations.

Discussion

At the conclusion of this study, we hope to assess the feasibility, effectiveness and implementation challenges of a complex DR care pathway integrating care at primary, secondary and tertiary care, covering a proportion of the diabetic population in Thiruvananthapuram. This pilot data will be useful for Kerala and perhaps India to plan to achieve the SDGs. The study will set the scene for a policy for State-wide Screening and Treatment Pathway for Diabetes Complications with clinical impact on the patient, impact on the community, impact on staff workload and economic impact on family and public finances and integrating it to primary care practice.[30] Early identification of STDR and other complications due to this holistic approach and timely treatment, will have positive impact on rates of blindness, chronic kidney disease, cardiovascular complications and thereby improve health, reduce multi-morbidity and mortality.

This protocol outlines how the effectiveness of a DR pathway and its implementation will be evaluated. [21, 22] In addition, a process evaluation will provide information on how this pathway can be integrated into everyday practice in the public health system.[20] This work is timely given the increasing numbers of people with diabetes and pressure on finances available for healthcare, necessitating task shifting to enable better coverage of the population. Although it is a DR care pathway, we will collect data on other complications of diabetes to address the wider needs of this group of patients. The study will also examine the organisation functions such as

For peer review only - http://bmjopen.bmj.com/site/about/guidelines.xhtml

structure, resources and processes that would contribute to better outcomes. We will also examine whether this additional task is feasible given the current skill levels and workload of FHCs and secondary hospitals. The study will reveal whether current health seeking behaviour of patients will support screening, especially when an invasive procedure is requested when there is no obvious impact on quality of life of the patient. By addressing a key gap in knowledge due to lack of research in this area, we will be able to decipher the barriers and facilitators that influence the successful implementation of such a programme in the public system in India. The results of this study will inform the adoption of this pathway in other areas in India and globally.

There are limitations, however, to the study design. We do not have baseline data on DR screening in the study location as these screening programmes are non-existent. For this reason, we are examining the effectiveness of the pathway in terms of presenting visual acuity for referable cases as 'proximal' outcomes. In addition, when there is no concurrent control group, causal inferences are difficult to make and may lead to both measured and unmeasured biases. When examining implementation, logistical issues, variations in health care facilities, socio-economic variation, and quality of health care personnel may likely affect our study. However, the study design avoids the ethical challenges of having a control group with no DR screening. Therefore, the study design is by necessity non-randomised and observational and will rely on newly trained staff members to collect data, which is likely to differ in completeness between the 16 FHCs. We expect an increase in referral rates after implementation of the intervention due to better public awareness, increasing knowledge of ASHAs and improved case ascertainment, at least in some FHCs. We will also examine the impact of cataract in the community and this has not been studied before in any other DR pathway. We have tried to minimise these risks by the use of robust statistical techniques and the triangulation of various data sources to elicit a greater understanding of how the programme will lead to better health outcomes that will include identification of cataract.

This study has many strengths: Our study has quantitative data backed by qualitative data to strengthen our findings and enable generalisation of our findings. Secondly, we will use robust statistical methods to reduce bias including selection bias and other confounders. Finally, the access to and collaboration with the UK is a key strength of the study, as it facilitates the co-development of the interventions from the outset. We expect the findings of this evaluation to be of interest to a wide audience given the growing need to demonstrate effectiveness of complex interventions in low resource settings. Findings on the mechanisms and contexts that optimise the implementation of this complex multi-faceted intervention will be useful to those developing

For peer review only - http://bmjopen.bmj.com/site/about/guidelines.xhtml

and implementing these programmes in other health systems. The health economic model may highlight the health expenditure required at individual, family and Kerala State level for forecasting and planning health budgets.

Perhaps the most important aspect of the chosen evaluation is that it is built within a simultaneous developing public health strategy on population-based screening of diabetes and hypertension and a recently introduced eHealth. Conducting research within this system strengthens data systems and quality for non-research purposes.

Despite the limitations, this study holds promise for providing high-quality data and detailed implementation information on a complex intervention in a resources-limited setting. We hope to contribute to the literature on the implementation and effectiveness of DR screening and treatment in the public sector in LMICs.

References

1. International Diabetes Federation. *IDF Diabetes Atlas*. 9th edition 2019 https://www.diabetesatlas.org/en/
2. Yau JW, Rogers SL, Kawasaki R, et al. Meta-Analysis for Eye Disease (META-EYE) Study Group. Global prevalence and risk factors of diabetic retinopathy. *Diabetes Care* 2012; 35:556–64.
3. IDF Diabetes Atlas: Global estimates of diabetes prevalence for 2017 and projections for 2045. Cho NH, Shaw JE, Karuranga S, Huang Y, da Rocha Fernandes JD, Ohlrogge AW, Malanda B Diabetes Res Clin Pract. 2018; 138():271-281.
4. Scanlon PH. The English National Screening Programme for diabetic retinopathy 2003-2016. *Acta Diabetol* 2017; 54(6):515-25
5. Cavan D, Makaroff L, da Rocha Fernandes J, et al. The Diabetic Retinopathy Barometer Study: Global perspectives on access to and experiences of diabetic retinopathy screening and treatment. *Diabetes Res Clin Pract* 2017; 129:16-24.
6. Menon VU, Kumar KV, Gilchrist A, et al. Prevalence of known and undetected diabetes and associated risk factors in central Kerala--ADEPS. *Diabetes Res Clin Pract* 2006; 74:289–94.
7. Sustainable Development Goals. United Nations. [Last accessed on 2020 May12]. Available from: http://www.un.org/sustainabledevelopment/sustainable-developmentgoals/
8. The WHO STEPwise approach to Surveillance of noncommunicable diseases (STEPS). Noncommunicable Diseases and Mental Health. World Health Organization. 20 Avenue Appia, 1211 Geneva 27, Switzerland.
9. Scanlon PH, Aldington SJ, Leal J, et al. Development of a cost-effectiveness model for optimisation of the screening interval in diabetic retinopathy screening. *Health Technol Assess* 2015;19(74):1-116.

For peer review only - http://bmjopen.bmj.com/site/about/guidelines.xhtml

10. Nguyen HV, Tan GS, Tapp RJ, et al. Cost-effectiveness of a National Telemedicine Diabetic Retinopathy Screening Program in Singapore. *Ophthalmology* 2016;123(12):2571-80.

11. Leese GP, Morris AD, Olson J. A national retinal screening programme for diabetes in Scotland. *Diabet Med* 2003;20(12):962-4.

12. Lund SH, Aspelund T, Kirby P et al. Individualised risk assessment for diabetic retinopathy and optimisation of screening intervals: a scientific approach to reducing healthcare costs. Br J Ophthalmol. 2016;100(5):683-7.

13. Manne-Goehler J, Geldsetzer P, Agoudavi K et al. Health system performance for people with diabetes in 28 low- and middle-income countries: A cross-sectional study of nationally representative surveys. PLoS Med. 2019;16(3):e1002751.

14. Ting DS, Cheung GC, Wong TY. Diabetic retinopathy: global prevalence, major risk factors, screening practices and public health challenges: a review. Clin Exp Ophthalmol. 201;44(4):260-77.

15. Hazin R, Barazi MK, Summerfield M. Challenges to establishing nationwide diabetic retinopathy screening programs. Curr Opin Ophthalmol. 2011;22(3):174-9.

16. Burgess PI, Msukwa G, Beare NA. Diabetic retinopathy in sub-Saharan Africa: meeting the challenges of an emerging epidemic. BMC Med. 2013;11:157.

17. Ramke J, Petkovic J, Welch V et al. Interventions to improve access to cataract surgical services and their impact on equity in low- and middle-income countries. Cochrane Database Syst Rev. 2017;11(11):CD011307.

18. Rani PK, Bhattarai Y, Sheeladevi S et al. Analysis of yield of retinal imaging in a rural diabetes eye care model. Indian J Ophthalmol. 2018 Feb;66(2):233-237.

19. Scanlon PH, Foy C, Malhotra R, Aldington SJ. The influence of age, duration of diabetes, cataract, and pupil size on image quality in digital photographic retinal screening. Diabetes Care. 2005;28(10):2448-53.

20. Moore GF, Audrey S, Barker M,et al. Process evaluation of complex interventions: Medical Research Council guidance. *BMJ* 2015;350:h1258.

21. Hasson H. Systematic evaluation of implementation fidelity of complex interventions in health and social care. *Implement Sci* 2010; 5:67.

22. Powell BJ, Waltz TJ, Chinman MJ et al. A refined compilation of implementation strategies: results from the Expert Recommendations for Implementing Change (ERIC) project. Implement Sci. 2015;10:21.

23. Landes SJ, McBain SA, Curran GM. Reprint of: An introduction to effectiveness-implementation hybrid designs. Psychiatry Res. 2020 Jan;283:112630.

24. Rajalakshmi R, Subashini R, Anjana RM, et al. Automated diabetic retinopathy detection in smartphone-based fundus photography using artificial intelligence.

For peer review only - http://bmjopen.bmj.com/site/about/guidelines.xhtml

Eye (Lond) 2018;32(6):1138-1144

25. Piyasena MMPN, Murthy GVS, Yip JLY et al. Systematic review and meta-analysis of diagnostic accuracy of detection of any level of diabetic retinopathy using digital retinal imaging. Syst Rev. 2018 Nov 7;7(1):182. doi: 10.1186/s13643-018-0846-y. Erratum in: Syst Rev. 2019 Apr 30;8(1):106.

26. Nanji K, Kherani IN, Damji KF et al. The Muranga Teleophthalmology Study: A Comparison of Virtual (Teleretina) Assessment with in-person Clinical Examination to Diagnose Diabetic Retinopathy and Age-related Macular Degeneration in Kenya. Middle East Afr J Ophthalmol. 2020;27(2):91-99.

27. Boucher MC, Nguyen MTD, Qian J. Assessment of Training Outcomes of Nurse Readers for Diabetic Retinopathy Telescreening: Validation Study. JMIR Diabetes. 2020;5(2):e17309.

28. Polack S, Alavi Y, Rachapalle Reddi S et al. Utility values associated with diabetic retinopathy in Chennai, India. Ophthalmic Epidemiol. 2015; 22:1, 20-27.

29. Becker N, Hakama M, Nyström L. Evaluation of effectiveness of quality-assured mammography screening in Germany: sample size considerations and design options. *Eur J Cancer Prev* 2007;16(3):225-31.

30. Grol R, Grimshaw J. From best evidence to best practice: effective implementation of change in patients' care. *Lancet* 2003;362(9391):1225–30.

Legend: Figure 1: Proposed Diabetic Retinopathy Care Pathway

Nayanamritham Project Collaborators.
Dr Rajeevan Palpoo and Dr Chitra Raghavan who managed the referred patients in Regional Institute of Ophthalmology; Alwin Aniyankunju and Anju V.Molly were the retinal graders for the project.
Co-ordinating Centre: Regional Institute of Ophthalmology
Steering Committee: Kerala Members of the International Advisory Board

Acknowledgements: The authors would like to thank all the Study collaborators including field workers, Director of Health Services; Director of Medical Education; Doctors and Nurses at Family Health Centres, Secondary Care and Tertiary Care Centres and Study Participants.
Patient and Public Involvement: Patients or the public will be involved in the conduct of the study, reporting and dissemination plans of our research
Funding statement: This work was supported by Global Challenges Research Fund and UK Research and Innovation through the Medical Research Council grant number MR/P027881/1
Data Sharing Agreement: The technical appendix, statistical code and dataset will be made available on request after obtaining permission from the Government of Kerala.
Competing interests statement: None

Sponsor: Regional Institute of Ophthalmology, Thiruvananthapuram, Kerala

For peer review only - http://bmjopen.bmj.com/site/about/guidelines.xhtml

Author Contributorship

Sobha Sivaprasad (S.S), Gopalakrishnan Netuveli (G.N), Raphael Wittenberg (R.W), Rajan Khobragade (R.K), Rajeev Sadanandan (R.S), Bipin Gopal (B.G), Lakshmi Premnazir (L.P), Dolores Conroy (D.C), Jyotsna Srinath (J.S), Radha Ramakrishnan (R.R), Simon George (S.G), Vasudeva Iyer Sahasranamam (V.S)

Conceptualisation, S.S, G.N, R.W, R.S, B.G, S.G, V.S; methodology, S.S, G.N, R.R, R.S, B.G, V.S, S.G, D.C, R.R, R.K; acquisition of data, S.S, G.N, R.W, D.C, R.R, J.S, L.P, B.G; writing—original draft preparation, S.S, D.C, G.N, R.W, L.P, R.R; writing—review and editing, S.S, G.N, R.R, R.S, B.G, V.S, S.G, D.C, R.R, R.K, J.S, L.P; funding acquisition, S.S, G.N, R.W, R.S, on behalf of the Collaborators.

Role of study sponsor and funders, if any, in study design; collection, management, analysis, and interpretation of data; writing of the report; and the decision to submit the report for publication, including whether they will have ultimate authority over any of these activities; None

For peer review only

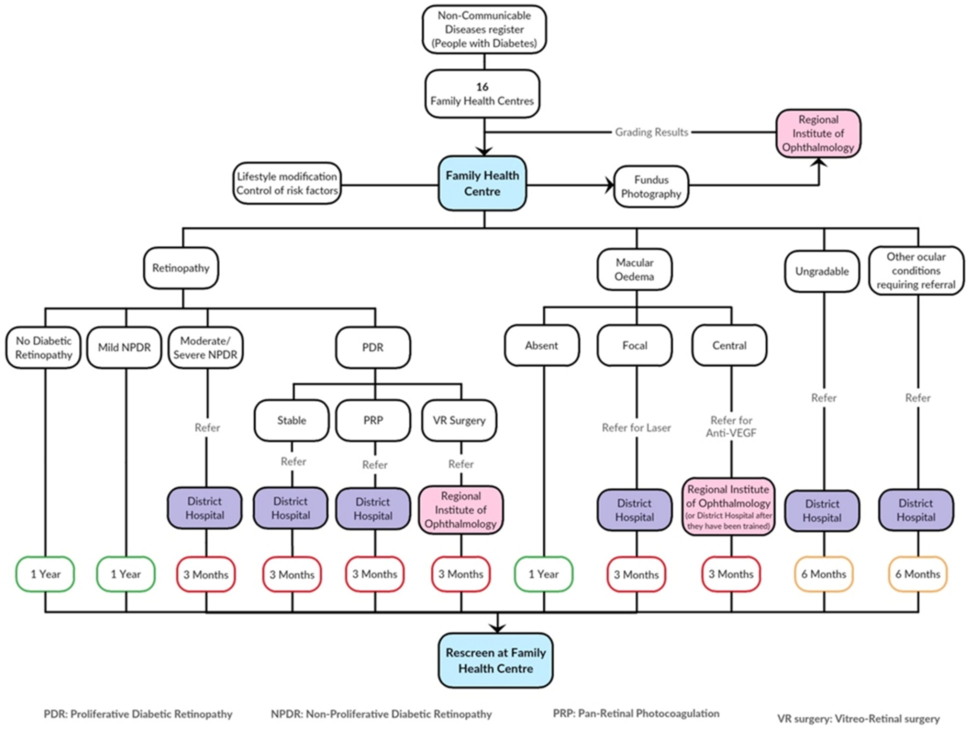

Figure 1: Flow Diagram

186x138mm (300 x 300 DPI)

SPIRIT
STANDARD PROTOCOL ITEMS: RECOMMENDATIONS FOR INTERVENTIONAL TRIALS

SPIRIT 2013 Checklist: Recommended items to address in a clinical trial protocol and related documents*

Section/item	Item No	Description	Addressed on page number
**Administrative information**			
Title	1	Descriptive title identifying the study design, population, interventions, and, if applicable, trial acronym	_1____________
Trial registration	2a	Trial identifier and registry name. If not yet registered, name of intended registry	__1___________
	2b	All items from the World Health Organization Trial Registration Data Set	__NA__________
Protocol version	3	Date and version identifier	_1____________
Funding	4	Sources and types of financial, material, and other support	__19_______
Roles and responsibilities	5a	Names, affiliations, and roles of protocol contributors	__19__________
	5b	Name and contact information for the trial sponsor	__19__________
	5c	Role of study sponsor and funders, if any, in study design; collection, management, analysis, and interpretation of data; writing of the report; and the decision to submit the report for publication, including whether they will have ultimate authority over any of these activities	__20__________
	5d	Composition, roles, and responsibilities of the coordinating centre, steering committee, endpoint adjudication committee, data management team, and other individuals or groups overseeing the trial, if applicable (see Item 21a for data monitoring committee)	__19_________

For peer review only

Introduction

Background and rationale	6a	Description of research question and justification for undertaking the trial, including summary of relevant studies (published and unpublished) examining benefits and harms for each intervention	__5___________
	6b	Explanation for choice of comparators	__7___________
Objectives	7	Specific objectives or hypotheses	__6__________
Trial design	8	Description of trial design including type of trial (eg, parallel group, crossover, factorial, single group), allocation ratio, and framework (eg, superiority, equivalence, noninferiority, exploratory)	___6__________

Methods: Participants, interventions, and outcomes

Study setting	9	Description of study settings (eg, community clinic, academic hospital) and list of countries where data will be collected. Reference to where list of study sites can be obtained	___6__________
Eligibility criteria	10	Inclusion and exclusion criteria for participants. If applicable, eligibility criteria for study centres and individuals who will perform the interventions (eg, surgeons, psychotherapists)	____6_________
Interventions	11a	Interventions for each group with sufficient detail to allow replication, including how and when they will be administered	___7__________
	11b	Criteria for discontinuing or modifying allocated interventions for a given trial participant (eg, drug dose change in response to harms, participant request, or improving/worsening disease)	____7-8_______
	11c	Strategies to improve adherence to intervention protocols, and any procedures for monitoring adherence (eg, drug tablet return, laboratory tests)	_____8________
	11d	Relevant concomitant care and interventions that are permitted or prohibited during the trial	_____NA_______ _
Outcomes	12	Primary, secondary, and other outcomes, including the specific measurement variable (eg, systolic blood pressure), analysis metric (eg, change from baseline, final value, time to event), method of aggregation (eg, median, proportion), and time point for each outcome. Explanation of the clinical relevance of chosen efficacy and harm outcomes is strongly recommended	___8-12__

For peer review only - http://bmjopen.bmj.com/site/about/guidelines.xhtml

Participant timeline	13	Time schedule of enrolment, interventions (including any run-ins and washouts), assessments, and visits for participants. A schematic diagram is highly recommended (see Figure)	_____Figure 1
Sample size	14	Estimated number of participants needed to achieve study objectives and how it was determined, including clinical and statistical assumptions supporting any sample size calculations	_12____________
Recruitment	15	Strategies for achieving adequate participant enrolment to reach target sample size	__8___________
**Methods: Assignment of interventions (for controlled trials)**			
Allocation:			
Sequence generation	16a	Method of generating the allocation sequence (eg, computer-generated random numbers), and list of any factors for stratification. To reduce predictability of a random sequence, details of any planned restriction (eg, blocking) should be provided in a separate document that is unavailable to those who enrol participants or assign interventions	_____________
Allocation concealment mechanism	16b	Mechanism of implementing the allocation sequence (eg, central telephone; sequentially numbered, opaque, sealed envelopes), describing any steps to conceal the sequence until interventions are assigned	_____________
Implementation	16c	Who will generate the allocation sequence, who will enrol participants, and who will assign participants to interventions	_____________
Blinding (masking)	17a	Who will be blinded after assignment to interventions (eg, trial participants, care providers, outcome assessors, data analysts), and how	_____________
	17b	If blinded, circumstances under which unblinding is permissible, and procedure for revealing a participant's allocated intervention during the trial	_____________
**Methods: Data collection, management, and analysis**			
Data collection methods	18a	Plans for assessment and collection of outcome, baseline, and other trial data, including any related processes to promote data quality (eg, duplicate measurements, training of assessors) and a description of study instruments (eg, questionnaires, laboratory tests) along with their reliability and validity, if known. Reference to where data collection forms can be found, if not in the protocol	_____14________

	18b	Plans to promote participant retention and complete follow-up, including list of any outcome data to be collected for participants who discontinue or deviate from intervention protocols	____8-12______
Data management	19	Plans for data entry, coding, security, and storage, including any related processes to promote data quality (eg, double data entry; range checks for data values). Reference to where details of data management procedures can be found, if not in the protocol	_______14______
Statistical methods	20a	Statistical methods for analysing primary and secondary outcomes. Reference to where other details of the statistical analysis plan can be found, if not in the protocol	____13_________
	20b	Methods for any additional analyses (eg, subgroup and adjusted analyses)	______13_______
	20c	Definition of analysis population relating to protocol non-adherence (eg, as randomised analysis), and any statistical methods to handle missing data (eg, multiple imputation)	____NA________ _
**Methods: Monitoring**			
Data monitoring	21a	Composition of data monitoring committee (DMC); summary of its role and reporting structure; statement of whether it is independent from the sponsor and competing interests; and reference to where further details about its charter can be found, if not in the protocol. Alternatively, an explanation of why a DMC is not needed	____15________
	21b	Description of any interim analyses and stopping guidelines, including who will have access to these interim results and make the final decision to terminate the trial	____NA________ _
Harms	22	Plans for collecting, assessing, reporting, and managing solicited and spontaneously reported adverse events and other unintended effects of trial interventions or trial conduct	_____NA_______ _
Auditing	23	Frequency and procedures for auditing trial conduct, if any, and whether the process will be independent from investigators and the sponsor	____NA________ _
**Ethics and dissemination**			
Research ethics approval	24	Plans for seeking research ethics committee/institutional review board (REC/IRB) approval	______15_______

Protocol amendments	25	Plans for communicating important protocol modifications (eg, changes to eligibility criteria, outcomes, analyses) to relevant parties (eg, investigators, REC/IRBs, trial participants, trial registries, journals, regulators)	___1__________
Consent or assent	26a	Who will obtain informed consent or assent from potential trial participants or authorised surrogates, and how (see Item 32)	____6__________
	26b	Additional consent provisions for collection and use of participant data and biological specimens in ancillary studies, if applicable	____NA_________ _
Confidentiality	27	How personal information about potential and enrolled participants will be collected, shared, and maintained in order to protect confidentiality before, during, and after the trial	_____15_________
Declaration of interests	28	Financial and other competing interests for principal investigators for the overall trial and each study site	___20___________
Access to data	29	Statement of who will have access to the final trial dataset, and disclosure of contractual agreements that limit such access for investigators	____20__________
Ancillary and post-trial care	30	Provisions, if any, for ancillary and post-trial care, and for compensation to those who suffer harm from trial participation	____NA_________ _
Dissemination policy	31a	Plans for investigators and sponsor to communicate trial results to participants, healthcare professionals, the public, and other relevant groups (eg, via publication, reporting in results databases, or other data sharing arrangements), including any publication restrictions	____15__________
	31b	Authorship eligibility guidelines and any intended use of professional writers	____20__________
	31c	Plans, if any, for granting public access to the full protocol, participant-level dataset, and statistical code	___20___________
**Appendices**			
Informed consent materials	32	Model consent form and other related documentation given to participants and authorised surrogates	_______NA_____ _
Biological specimens	33	Plans for collection, laboratory evaluation, and storage of biological specimens for genetic or molecular analysis in the current trial and for future use in ancillary studies, if applicable	________NA____ _

For peer review only - http://bmjopen.bmj.com/site/about/guidelines.xhtml

*It is strongly recommended that this checklist be read in conjunction with the SPIRIT 2013 Explanation & Elaboration for important clarification on the items. Amendments to the protocol should be tracked and dated. The SPIRIT checklist is copyrighted by the SPIRIT Group under the Creative Commons "Attribution-NonCommercial-NoDerivs 3.0 Unported" license.

For peer review only

BMJ Open

Complex interventions to implement a diabetic retinopathy care pathway in the public health system in Kerala: an effectiveness-implementation protocol of the Nayanamritham project.

Journal:	*BMJ Open*
Manuscript ID	bmjopen-2020-040577.R2
Article Type:	Protocol
Date Submitted by the Author:	19-Jan-2021
Complete List of Authors:	Sivaprasad, Sobha; UCL, NIHR Moorfields Biomedical Research; Moorfields Eye Hospital NHS Foundation Trust<br>Netuveli, Gopalakrishnan; University of East London - Duncan House Campus, Institute of Connected Communities<br>Wittenberg, Raphael; Oxford University, Nuffield Department of Primary Health Care Sciences<br>Khobragade, Rajan; Government of Kerala<br>Sadanandan, Rajeev; Government of Kerala<br>Gopal, Bipin; Government Medical College Thiruvananthapuram, Directorate of Health Services<br>Premnazir, Lakshmi; Government Medical College Thiruvananthapuram, Directorate of Health Services<br>Conroy, Dolores; UCL, Institute of Ophthalmology<br>Srinath, Jyotsna; University of East London - Duncan House Campus, Department of connected communities<br>Ramakrishnan, Radha; UCL, Institute of Ophthalmology<br>George, Simon; Government Medical College Thiruvananthapuram, Regional Institute of Ophthalmology<br>Sahasranamam, Vasudeva Iyer ; Government Medical College Thiruvananthapuram, Regional Institute of Ophthalmology
**Primary Subject Heading**:	Ophthalmology
Secondary Subject Heading:	Public health, Diabetes and endocrinology
Keywords:	Diabetic retinopathy < DIABETES & ENDOCRINOLOGY, General diabetes < DIABETES & ENDOCRINOLOGY, Protocols & guidelines < HEALTH SERVICES ADMINISTRATION & MANAGEMENT

SCHOLARONE™
Manuscripts

I, the Submitting Author has the right to grant and does grant on behalf of all authors of the Work (as defined in the below author licence), an exclusive licence and/or a non-exclusive licence for contributions from authors who are: i) UK Crown employees; ii) where BMJ has agreed a CC-BY licence shall apply, and/or iii) in accordance with the terms applicable for US Federal Government officers or employees acting as part of their official duties; on a worldwide, perpetual, irrevocable, royalty-free basis to BMJ Publishing Group Ltd ("BMJ") its licensees and where the relevant Journal is co-owned by BMJ to the co-owners of the Journal, to publish the Work in this journal and any other BMJ products and to exploit all rights, as set out in our licence.

The Submitting Author accepts and understands that any supply made under these terms is made by BMJ to the Submitting Author unless you are acting as an employee on behalf of your employer or a postgraduate student of an affiliated institution which is paying any applicable article publishing charge ("APC") for Open Access articles. Where the Submitting Author wishes to make the Work available on an Open Access basis (and intends to pay the relevant APC), the terms of reuse of such Open Access shall be governed by a Creative Commons licence – details of these licences and which Creative Commons licence will apply to this Work are set out in our licence referred to above.

Other than as permitted in any relevant BMJ Author's Self Archiving Policies, I confirm this Work has not been accepted for publication elsewhere, is not being considered for publication elsewhere and does not duplicate material already published. I confirm all authors consent to publication of this Work and authorise the granting of this licence.

For peer review only

Complex interventions to implement a diabetic retinopathy care pathway in the public health system in Kerala: an effectiveness-implementation protocol of the Nayanamritham project.

Sobha Sivaprasad[1, 2], Gopalakrishnan Netuveli[3], Raphael Wittenberg[4], Rajan Khobragade[5], Rajeev Sadanandan[5], Bipin Gopal[6], Lakshmi Premnazir[6], Dolores Conroy[2], Jyotsna Srinath[3], Radha Ramakrishnan[2], Simon George[7], Vasudeva Iyer Sahasranamam[7] for the Nayanamritham Project Collaborators.

1. Medical Retina Department, NIHR Biomedical Research Centre, Moorfields Eye Hospital NHS Foundation Trust and University College London, London, UK
2. Department of Vision Sciences, UCL Institute of Ophthalmology, London, UK
3. Institute of Connected Communities, University of East London, UK
4. Nuffield Department of Primary Health Care Sciences, University of Oxford, Oxford, UK
5. Directorate of Health, Government of Kerala, India
6. NCD department, Directorate of Health Services, Kerala, India
7. Ophthalmology department, Regional Institute of Ophthalmology, Thiruvananthapuram, Kerala, India

Word count: 3696

Figures: 2

Table: 3

References: 30

Abstract: 294

Corresponding author

Professor Sobha Sivaprasad
NIHR Moorfields Biomedical Research Centre
Moorfields Eye Hospital NHS Foundation Trust,
162 City Road,
London EC1V 2PD, UK
Tel: 020 7253 3411
Email: senswathi@aol.com

Keywords: diabetic retinopathy, implementation study, Kerala, laser photocoagulation, diabetes, screening, visual impairment, cost-effectiveness.

Trial Registration Number: ISRCTN28942696

Protocol Version: 1.0 dated 13-03-2019

For peer review only - http://bmjopen.bmj.com/site/about/guidelines.xhtml

Abstract

Introduction: This type-2 hybrid effectiveness-implementation study, a United Kingdom (UK) – Government of Kerala collaboration, aims to pilot a new diabetic retinopathy (DR) pathway in the public health system in Thiruvananthapuram district in Kerala to understand how it can be scaled up to and sustained in the whole state.

Methods and analysis: Currently, there is no systematic DR screening programme in Kerala. Our intervention is a teleophthalmology pathway for people with diabetes in the non-communicable disease (NCDs) registers in 16 family health centres (FHCs). The planned implementation strategy of the pathway will be developed based on the discrete Expert Recommendations for Implementing Change (ERIC) taxonomy. In this mixed methods study, we will utilise quantitative data from a cross-sectional study and qualitative data from all stakeholders obtained from structured interviews, surveys and group discussions to report the effectiveness of the DR pathway and evaluation of the implementation strategy.

The primary effectiveness outcome is the proportion of patients in the NCD register with diabetes screened for DR by 12 months. Other outcomes include safety, efficiency, patient satisfaction, timeliness and equity. The key cost-effectiveness outcome will be the number of cases of severe visual impairment or blindness due to STDR averted as a result of the new DR screening programme and care pathway. The comparator is the data on prevalence of newly identified DR in a house-to-house survey completed in a neighbouring district in a parallel study conducted by this group. The outcomes of evaluation of the implementation strategy include acceptability, adoption, appropriateness, feasibility, fidelity, penetration, costs and sustainability.

Ethics and Dissemination: The study was approved by Indian Medical Research Council (2018-0551) dated 13/03/2019. Study findings will be disseminated through scientific publications and the report will inform adoption of the DR care pathway by Kerala state in future.

For peer review only - http://bmjopen.bmj.com/site/about/guidelines.xhtml

ARTICLE SUMMARY

- This protocol describes effectiveness of a new diabetic retinopathy pathway within a resource constrained environment and the evaluation of the implementation strategy.
- The strength of the study is that a type 2- hybrid effectiveness-implementation will be tested using a mixed-methods approach.
- The specific actions in the implementation strategy are based on the Expert Recommendations for Implementing Change (ERIC) taxonomy
- The protocol outlines the economic evaluation of the costs of the screening programme and its outcomes.
- The protocol is limited by the absence of a comparator due to lack of previous data on the prevalence of DR in the public health system.

For peer review only

INTRODUCTION

Background

The triad of diabetes, blindness and poverty is an urgent problem that needs an effective response in the Development Assistance Committee-listed countries as these countries are home to 80% of people with diabetes. [1] Diabetic retinopathy (DR) is one of the most common complications of diabetes. [2] Sight-threatening diabetic retinopathy (STDR) is a common cause of blindness in working-age people and unless this condition is managed early, it has considerable impact on the quality of life and productivity of the person and their family, as well as substantial financial costs to health systems. [3] In its early stages, STDR may be asymptomatic. Therefore, DR screening programmes are essential to identify STDR to enable timely treatment. [4] In DR screening programmes in high income countries, people with diabetes are systematically screened using office-based retinal cameras and the retinal images are graded according to severity of DR by dedicated accredited screeners and graders. Patients with STDR are identified and referred to ophthalmologists for timely treatment with laser and/or intravitreal injections of anti-vascular endothelial growth factor. [4] In order to successfully reduce the risk of blindness due to DR in low-middle income countries (LMICs), without a well-developed primary care infrastructure, the pathway needs to begin with systematically screening for diabetes, educating the public and healthcare professionals on early detection[5] and timely treatment of STDR and the need to optimally treat the risk factors for DR such as hyperglycaemia, hypertension and hyperlipidemia. [2] Moreover, DR screening and treatment are challenging due to the required technology and technical expertise needed to grade retinal images and deliver costly treatment options, adding to the cost and complexity of the required interventions.

Diabetes in Kerala

Kerala is the most advanced state in India in terms of literacy, health, social uplift and demographic transition.[6] This has been accompanied by high prevalence of diabetes. A recent report from Kerala suggests that one in five of the Kerala adult population may have diabetes.[7] The Government of Kerala launched the Aardram Mission in 2017 to transform and gear up the State's public health care system to achieve the Sustainable Development Goals (SDGs) in phases with short term goals on building infrastructure and quality care services. The overarching objectives of the Aardram Mission included providing equitable, affordable and quality care to citizens from all socio-economic strata; strengthening the public care system by

For peer review only

decentralising healthcare from the secondary and tertiary level to primary care-led services and initiating preventive medicine to address the impact of NCDs, especially hypertension and diabetes. Primary care centres have been converted to family health centres (FHCs) with three doctors and four nurses in each FHC assigned to provide individual care plans to the allocated population whose records are tracked through a recently established Electronic Health Record (eHealth). The transformation of primary with a focus on NCDs provided the backdrop to the implementation of a DR care pathway.

The rationale for a complex DR pathway

The Government of Kerala is pressing forward to achieve universal health coverage and address the SDG goals on poverty (SDG 1), health access (SDG 3), education (SDG 4) and gender equality (SDG 5). [8, 9] Therefore, there is an urgent need to tackle the complications of diabetes. Systematic DR screening has been shown to be effective in reducing blindness. [3] However, in high income countries, DR screening is achieved by dedicated services due to the technicalities and expertise required in DR screening and evaluation. [10-12] Introducing an isolated DR programme will not be sufficient to address the challenge in LMICs as these countries need to initiate holistic screening service for diabetes and all its complications simultaneously and embed DR screening within the primary care.

Rationale for an Implementation Strategy

Each aspect of the DR screening and care pathway has to be adapted to local needs and resources, requiring a locally appropriate implementation strategy. For example, mydriasis (pupil dilatation) is compulsory in some screening programmes but not in others and this process requires local approval by stakeholders. [10-12] In Kerala, mydriasis needs to be approved by the health department. Other issues faced by LMIC are that the number of undiagnosed diabetes cases is high, [13] the non-availability of a state-wide diabetes register with recall facilities, the lack of resources for standardised non-portable retinal cameras, limited capacity of staff in primary care to screen for DR, and that the number of people with diabetes is larger than the capacity of the services provided by the secondary care hospitals. [14-16] Research capacity and capability are in their infancy in LMICs and implementing change in a busy environment when demands on staff are great and resources are limited is challenging. [15, 16] In addition, as cataract is more prevalent in LMIC, [17] the proportion of ungradable retinal images is higher compared to developed countries. [17-19] Therefore, we need to evaluate the implementation strategy using both qualitative and quantitative evaluation. [20-22]

For peer review only - http://bmjopen.bmj.com/site/about/guidelines.xhtml

Aims and Objectives

The Nayanamritham project is facilitated by a UK-Government of Kerala partnership as a part of the ORNATE-India project funded by Global Challenges Research Fund (GCRF) and UK Research and Innovation (UKRI). We aim to introduce a DR care pathway that spans primary, secondary and tertiary care in the public health system in Kerala in a pilot study in Thiruvananthapuram. The proposed study will (1) examine the clinical and cost effectiveness of the DR care pathway and (2) evaluate the implementation strategy of the pathway.

METHODS

Design: We chose a type 2- hybrid effectiveness-implementation study using a mixed-methods approach to evaluate the effectiveness of the clinical interventions and the implementation strategies. [20-23] The DR pathway and the study protocol were developed by the Kerala Health Secretary, NCD lead, Health and Medical Education service providers ,technical and eHealth teams, local authorities and the GCRF/UKRI- funded co-applicants from the UK. The DR care pathway will be set-up in a staggered approach with 5 FHCs initiated in the first three months and the remaining 11 FHCs will be added based on acceptance of all stakeholders and adapting and training phase for 12 months from 15-03-2018. A further 12-15 months will be allocated to recruiting consenting patients to a cross-sectional survey to gather and enter quantitative data in a study database. A minimal dataset will also be entered into eHeath to enable future screening for the consented patients. Qualitative data collection from interviews of staff, patients and focus groups at baseline and end of study and all field notes gathered during the study will be utilised to inform evaluation of the implementation strategy. [20-22]

Target Population

People with diabetes registered in the NCD register at 16 FHCs in Thiruvananthapuram district will be invited to be screened for complications of diabetes including screening for DR. As the NCD register is likely to show an increase in newly diagnosed diabetes as a result of training the accredited social health activists (ASHAs), in diabetes and DR, we will evaluate the effectiveness of the complex interventions delivered at each of the 16 FHCs for this study. The implementation of the pathway will take into account all patients in the NCD register at the start of the cross-sectional study.

For peer review only - http://bmjopen.bmj.com/site/about/guidelines.xhtml

Setting:

Target sites for implementation of DR screening and treatment in Thiruvananthapuram will include 16 FHCs for DR screening which represents the primary care centres where patients are screened for all complications of diabetes. The retinal images, captured by the trained, resident nursing staff will be sent to a newly developed reading centre at the Regional Institute of Ophthalmology (RIO), a tertiary care centre, where newly accredited graders will grade the images. Patients with screen-positive images will be referred to three secondary care hospitals (district hospitals). Severe cases that require complex interventions will be referred to the tertiary care centre, the RIO for specialist management of DR

Description of the standard of care

Currently, patients with diabetes are not systematically screened for DR in the public health system. Most patients present to the tertiary centre voluntarily either because of increased awareness of complications of diabetes or due to visual impairment. Therefore, the current standard of care will be captured as the number of patients presenting to the tertiary centre for an eye consultation for DR over a period of time as there is no baseline data in the primary and secondary care. This cross-sectional survey of the patients presenting at the FHCs will provide information on the uptake of screening of people registered in the NCD register at the start of the study. The prevalence of DR and STDR of the screened population will be estimated from the numbers screened in all the NCD registers during this period.

Evidence-based clinical intervention:

The new DR care pathway is the intervention and is shown in Figure 1. The pathway will span primary, secondary and tertiary care. The components of the pathway are:

1. DR screening of patients with diabetes registered in the NCD register at FHCs (primary care). The retinal images will be graded remotely at the reading centre at RIO.
2. Prompt referral for timely treatment of STDR to secondary care and tertiary centres depending on the severity of the DR.
3. Treatment of patients with sight threatening DR (at secondary and tertiary care).

Implementation strategies

For peer review only - http://bmjopen.bmj.com/site/about/guidelines.xhtml

244 The implementation strategies of new DR pathway are categorised as shown in Table 1 into plan, finance, education, , infrastructure, quality improvement and policy context. These categories are developed based on the discrete Expert Recommendations for Implementing Change (ERIC) taxonomy.[20, 21] The logic model is shown in Figure 2.

249 **Table 1: Implementation strategies**

Categories of Implementation Strategies[21]	Discrete Implementation Strategy[20]	Specific actions
Plan	Conduct local needs assessment	The study team will conduct a situational analysis of current pathway and collect and analyse data related to the need for a systematic DR pathway
	Assess for readiness and identify barriers and facilitators	Assess barriers that may impede implementation of the DR care pathway, and strengths that can be used in the implementation effort by interviewing key stakeholders i.e. FHC staff, ophthalmologists in secondary and tertiary care, health service employees and patients.
	Develop a formal implementation blueprint	The blueprint will be used to guide the implementation of the whole DR pathway and will be updated with time.
	Tailor strategies	Regular collaborative meetings will be held to tailor the implementation strategies to address barriers and leverage facilitators that are identified during various steps in the implementation.
	Stage implementation scale up and integration	The implementation will be phased with five FHCs starting as pilots to enable small changes before gradually moving to other 11 FHCs. Ensure integration of the DR pathway into the eHealth programme before roll-out to the whole state.
	Build a coalition	Cultivate relationships with staff at FHCs and secondary care centres, data entry operators, eHealth team, project manager, and Government of Kerala health service departmental staff in the implementation effort and create a learning system to incorporate practitioner feedback.
	Develop academic partnerships	Partner with UK academic institutions for the purposes of shared training and bringing research skills to the implementation of the pathway

For peer review only - http://bmjopen.bmj.com/site/about/guidelines.xhtml

	Recruit, designate, and train for leadership	A project manager will be recruited to manage the programme under the supervision of the NCD lead. Health Secretary will conduct periodic meetings with the project team to assess performance.
	Obtain formal commitments	A collaborative agreement is in place between Government of Kerala and Moorfields Eye Hospital, London ,UK outlining their roles and responsibilities in the implementation of the DR pathway.
	Prepare patients to be active participants	Community level health workers will prepare patients to be active in their care, to ask questions, and specifically to inquire about care guidelines, the evidence behind clinical decisions, or about available evidence-supported treatments. Each patient will provide written consent to be screened for DR in this project as well as participate in interviews after verbal consent
	Inform local opinion leaders	Health Minister of the state and members of the local governments will be briefed on the project and encouraged to be champions of the project to enable scale up to all FHCs in the state.
Finance	Access new funding	Use GCRF/UKRI funding to purchase smartphone retinal cameras and retinal lasers, develop eHealth, training staff at FHC and secondary care, employ project manager and data entry operators and personnel to conduct interviews, create study database linked to hand-held applications for data entry operators. State and local government funds will be sourced for scale up.
	Incentivisation	ASHAs will be incentivised to accompany referred patients to secondary care.
Education	Develop educational materials	Develop DR training modules and retinal image capture modules for FHC staff Develop laser training material to train ophthalmologists.
	Distribute educational materials	Distribute educational materials (including guidelines, manuals, and toolkits) in person, by mail, and/or electronically. Multiple training sessions are planned at each FHC. The camera manufacturers will be invited to provide hands-on training on capturing good quality retinal images. Training on laser surgery will also be provided to the ophthalmologists at secondary care.

For peer review only - http://bmjopen.bmj.com/site/about/guidelines.xhtml

	Conduct educational meetings	Hold meetings targeting different stakeholder groups (ophthalmologists, all FHC staff, ASHAs, patients in FHCs). Three educational meetings planned by the UK team to meet with the providers in the FHC settings to share knowledge and educate providers about the DR pathway and its integration to their clinical practice
	Achieve accreditation for graders of retinal images	Train and certify the optometrists in the Reading Centre in RIO to obtain accreditation as graders using the online international Test and Training for retinal graders.
	Conduct ongoing training	Nurses and doctors in the FHCs will receive on-going training on DR, pupil dilatation and capture of retinal images using low-cost smartphone retinal cameras.[24, 25]
	Create a learning collaborative	The Health Secretary of State will foster a collaborative learning environment to improve implementation of the DR care pathway
	Use train-the-trainer strategies	The UK team will first train a batch of FHC nurses to capture retinal images and then some will be trained to train the rest of the nurses. The same cascading pathway will apply to laser training.
	Work with educational institutions	Moorfields Eye Hospital staff will provide the necessary guidance and share knowledge on UK diabetic retinopathy screening pathway.
	Make training dynamic	All staff involved will receive continual online or face to face training.
	Increase demand	The ASHAs will be trained to increase public awareness of this pathway in their house-to-house health visits.
	Provide ongoing consultation	The UK team from Moorfields Eye Hospital will provide ongoing consultation to support implementation of DR care pathway.
Infrastructure	Centralize technical assistance	The project manager and the eHealth team will provide central assistance focused on implementation issues. The telemedicine project will link the patient's retinal images to the eHealth and transferred through a secure cloud to RIO, the tertiary centre, where the images will be graded for their DR status in a newly developed reading centre, with the results fed back to the FHCs.[26]
	Change physical structure and equipment	Evaluate current configuration of FHC consulting rooms to allow suitable furniture

For peer review only - http://bmjopen.bmj.com/site/about/guidelines.xhtml

		for cameras and adapt illumination to capture retinal photographs
	Change record systems	Change records systems to allow better assessment of implementation or clinical outcomes by having data entry operators interview patients and input full data into eHealth within the busy FHC clinics
	Change service sites	Change the timetable for clinics in secondary care to allow a patients referred from primary care to access treatment.
	Revise professional roles	The NCD lead will be delegated to take on the responsibility of implementing this pathway in Thiruvanthapuram with aim for Kerala state-wide roll out.
Quality Improvement	Develop and implement tools for quality monitoring	Develop, test, and introduce into quality-monitoring systems the right input—the appropriate language for the consent form, protocols on DR screening, grading retinal images, and measures of processes, patient outcomes, and implementation outcomes.
	Develop and organize quality monitoring systems	Develop and organise systems and procedures that monitor clinical processes and/or outcomes for the purpose of quality assurance and improvement
	Audit and provide feedback	Collect and summarise clinical performance data every month and feedback to FHCs and secondary care staff to monitor, evaluate, and modify pathway for acceptability to patients and staff. A continuous monitoring of retinal image quality and gradability, and the referral pathway will be done to cyclically input change into the DR pathway to improve the quality and integration
	Conduct cyclical small tests of change	Staggered set up of FHCs to allow implementation of changes in a cyclical fashion using small tests of change before taking changes system-wide. Tests of change benefit will be measured continually by increase uptake of training, screening, referral and treatment.
	Use data experts	The eHealth team will develop an application for the data entry operators to collect data. The UK collaborators will collaborate in the analysis of the data.
	Use data warehousing techniques	Integrate clinical records from this research project into eHealth on an on-going basis.
	Capture and share local knowledge	Capture local knowledge from implementation sites on how staff and doctors make something work in their FHCs and then share it with other FHCs.

For peer review only - http://bmjopen.bmj.com/site/about/guidelines.xhtml

	Obtain and use patients feedback	The participating patients will be asked to provide feedback on the implementation effort.
	Promote adaptability	Identify the ways the pathway can be tailored to meet local needs and clarify which elements of the DR pathway must be maintained to preserve fidelity. As an example, should patients be screened daily or should they be allocated to a single weekly clinic to improve efficiency?
	Provide clinical supervision	The Director of the RIO will provide the overall clinical supervision of the whole DR pathway. He will also delegate ophthalmologists to provide ongoing supervision for the optometrists involved in grading retinal images.
	Provide local technical assistance	Develop and use a system to deliver technical assistance focused on implementation issues using relevant service providers.
	Purposely re-examine the implementation	Monthly monitoring of screened patients at each FHCs, barriers observed and small changes that are required will be implemented to continuously improve the pathway.
	Remind clinicians	Primary care doctors in FHCs will receive monthly newsletter on the numbers recruited across all FHCs to highlighting successes and providing potential solutions to issues encountered.
Policy contexts	Issue Government of Kerala approval to enable dilatation of pupils of patients in FHC under supervision of FHC doctors	Ensure doctors at FHCs receive approval for mydriasis in order to deliver the diabetic retinopathy pathway.
	Mandate change	The Health Secretary of State will provide leadership to ensure the prioritisation of the implementation of the DR care pathway

FHCs -family health centres; DR-diabetic retinopathy; NCD-non-communicable diseases; ASHAs -accredited social health activists; GCRF/UKRI -Global Challenge Research Fund/UK Research and Innovation; RIO –Regional Institute of Ophthalmology

Outcomes

Pre-specified outcomes of the effectiveness of the DR pathway and the evaluation of the implementation strategy are tabulated in table 2 and table 3, respectively.

For peer review only

Table 2: DR pathway- (intervention) related outcomes

Outcomes	Source of data	Indicators
Clinical Effectiveness	Data from monthly eHealth entry, study database.	– Proportion of people screened for DR in each FHC over 12 months.<br>– Proportion of referred patients with STDR.<br>– Proportion of ungradable retinal images and percentage of cataract identified from referring these patients.<br>– Risk and complication burden in people screened for DR<br>– Presenting visual acuity of those referred from the DR pathway compared to those who self-referred to secondary care.<br>– Generalisability of the prevalence data compared to house-to-house survey in a neighbouring district.
Safety	eHealth and study database	Data on complications of the DR pathway
Cost-effectiveness	Data collected in the study baseline, data from the literature and expert opinion where data could not be collected.	The key outcome is the number of cases of severe visual impairment or blindness due to STDR averted as a result of the new DR care pathway and resultant QALY gain.<br>Estimated QALY gain from laser treatment of STDR and from cataract surgery, using utility values from Rachapelle et al 2013.[27]<br>Assumption that the effect of no screening is as in historic studies that reported the rate at which STDR leads to blindness if left untreated.
Efficiency	Capture efficiencies in structures, resources and processes across the DR pathway from staff interviews.<br><br>Path process mining of patients screened in FHCs analysis will be based on detailed time-event logs of 70 patients from the 16 FHCs	Efficiency is defined as optimal use of the service. For this purpose, we will first estimate the time required to process one patient from the path-process analysis. This estimate will be used to determine the number of patients who can be seen on a day. We will then calculate (1-number of patients screened)/number of patients that can be screened). proportion can be used in a p-type control chart.[28]

For peer review only

For peer review only - http://bmjopen.bmj.com/site/about/guidelines.xhtml

Patient satisfaction	Patient interviews and telephone survey of referred patients.	Patients willingness to be screened and their satisfaction with the information provided to them and the care they received
Timeliness	Path process mapping at FHCs to understand delays in screening pathway<br><br>Patient survey and secondary care records	Path process mapping at FHCs to understand delays in pathway<br>Reasons for non-attendance for those referred to secondary care for treatment of STDR
Equity	Data from eHealth and study database.	Age and gender-based prevalence of DR and STDR will be reported on consented patients from all FHCs. Reasons of non-referral of patients that require referrals will be recorded. Numbers denied screening despite willingness to be screened will also be reported.

FHCs -family health centres; DR -diabetic retinopathy; STDR-sight threatening diabetic retinopathy; QALY- quality-adjusted life year

Table 3: Outcomes of the evaluation of the implementation strategy

Outcomes	Indicators	Source of data and assessment
Acceptability	Willingness of each FHC to be involved in the DR pathway.	- Readiness of implementation of DR screening in the 16 FHCs and reasons for delay.<br>Data source: Clinical performance from FHCs
	Staff willingness to train	– Proportions of trained ASHAs ,<br>nurses and doctors per FHC, increase in laser trained doctors in secondary care, proportions willing to be included in train the trainer programme. The denominator will be total numbers of each personnel available to be trained on the date of implementation.<br>Data sources: Accreditation and certification records; Structured interviews of at least 5 nurses and 5 primary care doctors from 16 FHCs, 3-5 ophthalmologists from secondary and tertiary care, 2 data entry operators and 5 ASHAs and 1 health service administrator to understand challenges for acceptance.
	Patients acceptability of the pathway	-Proportions consenting to be screened per month; review feedback from interviews.<br><br>-Screening attendances per FHC over 12 months.

For peer review only - http://bmjopen.bmj.com/site/about/guidelines.xhtml

		Data sources: Clinical performance data and a telephone questionnaire survey for screened and referred patients to evaluate reasons for non-attendance.
Adoption	Uptake of DR care pathway at FHCs, secondary and tertiary care<br><br>Uptake of training by staff<br><br>Uptake of screening programme by patients<br><br>Adoption of DR screening pathway using the strategy as part of the package of services at FHCs by the Government of Kerala based on the clinical and cost-effectiveness data from this study.	-Proportion of staff willing to integrate DR pathway in their workload.<br>Data source: FHC data and staff interviews<br><br>– Demand for number of training sessions<br>– Attendance rates at training sessions<br>Data source: number of training sessions and attendance registers at training sessions.<br><br>– Increase in proportions of patient screened<br>Data source: eHealth and study database.<br><br>Data source: Policy paper to scale-up to other districts
Appropriateness	Appropriateness to locate the DR screening in FHCs<br><br>Appropriateness of the complex pathway across primary and secondary care<br><br>Appropriateness of referrals to secondary care	Data sources: interview of staff at FHCs and secondary care<br>Patient telephone survey on pathway from FHCs to secondary care<br><br>– False positive referrals from data collection at secondary care.<br>Data source: study database; data from patient telephone survey
Cost	Costs to the Government of Kerala and societal costs of the DR screening programme and subsequent treatment	Costs of the screening programme and treatment costs in total and per person, including the costs of staff training and cameras (suitably annuitized), staff costs and travel costs of patients.<br>Data sources: Data collected in the study baseline and data from other services in India and from the literature.

For peer review only - http://bmjopen.bmj.com/site/about/guidelines.xhtml

Feasibility	Screening rate per FHC<br>Barriers to monthly screening uptake	Data source:<br>Interview on facilitators and barriers on each part of the pathway<br>Data on monthly recruitment per FHC.
Fidelity	Degree of the DR care pathway implementation	Fidelity evaluation in 16 FHC clinics using a path-process analysis of the screening to identify the common screening pathway across FHCs.<br>Data source: The path-process analysis will be based on detailed time-event logs of 70 patients from the 16 FHCs; a telephone follow-up of referred patients to evaluate uptake of referrals.
	Validation of the DR grading within the telemedicine pathway	Validation of the DR grading will be assessed by the agreement of the DR grade reported by the optometrists at the reading centre in RIO and those reported by the ophthalmologists in the secondary care centres. Data source: DR grading of referred patients obtained from the reading centre versus secondary care.
	Delivery of training and the impact on the DR care pathway	Data sources: Staff interviews, structured observations and review of facility records. The proportions of doctors trained on laser delivery; data quality on the database; Increase in NCD registration following start of programme due to training of ASHA workers on diabetes and DR; Increase in numbers of patients treated for STDR following training.
Penetration	Increase in awareness and uptake of the DR screening pathway in patients with diabetes	Data from NCD register and eHealth
Sustainability	Policy makers to prepare policy on up-scale and sustainability plan for DR pathway, acceptance of guidelines and training material. Development of DR integration in eHealth in other districts	Evidence includes:<br>Policy paper<br>Scale up plan for other districts<br>Integration of DR pathway in eHealth NCD register

For peer review only - http://bmjopen.bmj.com/site/about/guidelines.xhtml

FHC -family health centres; DR-diabetic retinopathy; NCD-non-communicable diseases; ASHA - accredited social health activists

Data collection

Quantitative data

Effectiveness outcomes will originate from the data sources shown in table 2. Data collected include age, gender, education, occupation and income categories; duration of diabetes, use of insulin, parental history of diabetes, other complications of diabetes including diabetic kidney disease, cardiovascular complications, and diabetic foot. Other study-specific tests done on the day of screening include random blood sugar estimation, urine dipstick test for albuminuria, blood pressure record, body mass index, waist circumference, completion of a lifestyle questionnaire on smoking, diet, physical activity, vision related quality of life and previous history of DR, cataract surgery or any other ocular history.

In the reading centre, the data collection will include the grade of retinopathy in both eyes, presence of cataract and gradability of the retinal images. Data collected on referred patients will include numbers with ungradable images due to cataract, treatment options offered for DR and review appointment.

Qualitative data collection and analysis

Data sources used for evaluation of the implementation strategy are shown in table 3. These will include data from a structured interview of a maximum variable sample that will reflect the context (primary, secondary and tertiary care), and the functions and skill levels of the staff (e.g. nurses, doctors etc.). Based on pragmatic considerations, at least 5 nurses and 5 primary care doctors from 16 FHCs, 3-5 ophthalmologists from secondary and tertiary care, 2 data entry operators and 5 ASHAs and 1 health service administrator should be included to get the maximum variability. Verbal consent will be obtained from these health professionals. The interviews will be conducted by an independent member from the GCRF/UKRI-funded team, who is not involved in this study, in the local language within the premises of the healthcare provider. The focus group will consist of groups of patients and staff within one FHC. In addition, to the voice-recording of the interviews and focus group, interviewers will write field notes to

For peer review only - http://bmjopen.bmj.com/site/about/guidelines.xhtml

describe the interview situation. Field notes, the interviews and focus group content will provide the basis for the data analysis, which will be based on a descriptive phenomenological approach without data or opinion interpretation and will include transcription, condensation, coding and categorisation using qualitative analysis tools. A survey of all referred patients will be done using a structured questionnaire to evaluate their satisfaction and their perception of the barriers and facilitators. All qualitative data will be coded using NVivo and analysed using thematic analysis following the strategy.[29]

Monitoring:

Data will be coded before the clinical teams enter them into the study database. Only anonymised extracted data from eHealth will be used for analysis. Data quality will be checked by the data entry operators and monitored by the study project manager. Anonymised data will be checked for range checks and data quality at University of East London by the study statistics team. The ORNATE India International Advisory Board will have an overview of the conduct of the project and the Executive Group consisting of the UK-India collaborators will monitor the conduct of the study.

Sample size

We have chosen the proportion of patients in the NCD register with diabetes screened for DR as our primary outcome variable.

Justification of the choice of primary outcome: This variable captures the effectiveness of our intervention as well as the fidelity of implementation. Other facts that informed our choice of the primary outcome is that we expect a short implementation time of 7 to 9 months during which other outcomes such as number of patients treated may not be a feasible option. Therefore, we have estimated the sample size based on this primary outcome.

Our calculations are complicated by the expected increase in number of people with diabetes in the NCD register as public awareness of diabetes and DR increases so the denominator of numbers screened will be the number of people registered in the NCD in each FHC at the start of the cross-sectional study. There is no data on the baseline proportion of patients that attend the tertiary centre for screening. The prevalence of diabetes is between 10-16% in Thiruvananthapuram of which 8% are estimated to have DR and 3% to have STDR but about 20% may have to be referred. Assuming a finite population of 40,000 patients with diabetes, a simple random sample of 377 patients will be needed. However, we expect large design effects due to clustering patients within FHCs. There is not enough data to calculate the value of this design effect and therefore we assume it to be 3 to give a final sample size of 1,131, which is

For peer review only

equivalent to assuming a within-FHC intracluster correlation coefficient of approximately 0.03 (n=16 FHCs) with negligible residual clustering by the intermediate cluster level of ASHAs, where mean cluster size is smaller.

As described by Becker et al in mammography screening,[30] we expect sources of bias in the implementation programme that will likely influence the sample size calculation. Unscreened DR cases that already exist in the community may contribute to an over-estimation of the effect of screening. We expect lead-time bias because of our short implementation period and so some DR in the community may be missed. A comparison of numbers with DR in FHC with longer implementation period with those of shorter period may adjust for this bias. We intend to provide a descriptive analysis as well as a temporal comparison using time series analysis.

Statistical Analyses

We will create a complete case dataset of the cross-sectional survey for use in the analyses of effectiveness and assess the potential impact of missing data using sensitivity analysis incorporating a range of optimistic and pessimistic scenarios for the impact of missing data. We will model two outcomes that we hypothesised would be influenced by the intervention: (1) uptake of DR screening (primary outcome) and (2) numbers of patients referred for STDR as a result of screening. We will use bivariate logistic regression models to assess crude associations between sociodemographic factors such as age, gender, education, income, and living arrangements, and other clinical data with DR and STDR and use fractional polynomials to account for the form of continuous covariates to predict uptake of DR screening. Factors associated with a $P<0.25$ will be candidates for inclusion in the multivariable logistic regression models. We will test interactions amongst identified main effects to capture improvement in model prediction assessed by reduced residual variance-based statistics. In our final adjusted model, we will consider associations with a $P<.05$ as statistically significant. Adequacy of model discrimination and calibration will be assessed using Receiver Operating Characteristic (ROC) curves. The validation of the telemedicine will be reported as the agreement (kappa statistics) between screen positive patients graded by the graders at the reading centre versus the DR grade as recorded by the ophthalmologists in secondary care centres.

Patient and public involvement (PPI)

For peer review only - http://bmjopen.bmj.com/site/about/guidelines.xhtml

A patient and public group will be involved in plans to disseminate the study results and provide their input on the scale-up of this DR pathway, should the implementation in Thiruvananthapuram be deemed successful by the Government of Kerala.

Ethics and dissemination

The study was approved by Indian Medical Research Council (2018-0551).The study complies with the guidelines of the Declaration of Helsinki. Results of this research are expected to be disseminated through stakeholder reports and via scientific forums, specifically peer-reviewed publications and conference presentations. All participants will give written informed consent prior to entry to the study by the FHC nurses and will be made aware that participation is strictly voluntary.

Discussion

At the conclusion of this study, we hope to assess the feasibility, effectiveness and implementation challenges of a complex DR care pathway integrating care at primary, secondary and tertiary care, covering a proportion of the diabetic population in Thiruvananthapuram. This pilot data will be useful for Kerala and perhaps India to plan to achieve the SDGs. The study will set the scene for a policy for State-wide Screening and Treatment Pathway for Diabetes Complications with clinical impact on the patient, impact on the community, impact on staff workload and economic impact on family and public finances and integrating it to primary care practice. Early identification of STDR and other complications due to this holistic approach and timely treatment, will have positive impact on rates of blindness, chronic kidney disease, cardiovascular complications and thereby improve health, reduce multi-morbidity and mortality. A DR pathway that straddles primary, secondary and tertiay levels of care, leveraging technology will have advantages of cost effectiveness and ease of implication in LMICs as against the current practice of detection and management of self reported cases in tertiary centres.

This protocol outlines how the effectiveness of a DR pathway and its implementation will be evaluated. [21, 22] This work is timely given the increasing numbers of people with diabetes and pressure on finances available for healthcare, necessitating task shifting to enable better coverage of the population. Although it is a DR care pathway, we will collect data on other complications of diabetes to address the wider needs of this group of patients. The study will also examine the organisation functions such as structure, resources and processes that would

For peer review only - http://bmjopen.bmj.com/site/about/guidelines.xhtml

contribute to better outcomes. We will also examine whether this additional task is feasible given the current skill levels and workload of FHCs and secondary hospitals. The study will reveal whether current health seeking behaviour of patients will support screening, especially when an invasive procedure is requested when there is no obvious impact on quality of life of the patient. By addressing a key gap in knowledge due to lack of research in this area, we will be able to decipher the barriers and facilitators that influence the successful implementation of such a programme in the public system in India. The results of this study will inform the adoption of this pathway in other areas in India and globally.

There are limitations, however, to the study design. We do not have baseline data on DR screening in the study location as these screening programmes are non-existent. For this reason, we are examining the effectiveness of the pathway in terms of presenting visual acuity for referable cases as 'proximal' outcomes. In addition, when there is no concurrent control group, causal inferences are difficult to make and may lead to both measured and unmeasured biases. When examining implementation, logistical issues, variations in health care facilities, socio-economic variation, and quality of health care personnel may likely affect our study. However, the study design avoids the ethical challenges of having a control group with no DR screening. Therefore, the study design is by necessity non-randomised and observational and will rely on newly trained staff members to collect data, which is likely to differ in completeness between the 16 FHCs. We expect an increase in referral rates after implementation of the intervention due to better public awareness, increasing knowledge of ASHAs and improved case ascertainment, at least in some FHCs. We will also examine the impact of cataract in the community and this has not been studied before in any other DR pathway. We have tried to minimise these risks by the use of robust statistical techniques and the use of various data sources to elicit a greater understanding of how the programme will lead to better health outcomes that will include identification of cataract.

This study has many strengths: Our study has quantitative data backed by qualitative data to strengthen our findings and enable generalisation of our findings. Secondly, we will use robust statistical methods to reduce bias including selection bias and other confounders. Finally, the access to and collaboration with the UK is a key strength of the study, as it facilitates the co-development of the interventions from the outset. Active involvement of policy makers engaged in transformation of primary care to screen for and address complications of NCDs who value research to generate evidence for policy making and who are prepared to learn from, and adapt the DR based on, implementation experience is a unique feature of this study. We expect the

For peer review only - http://bmjopen.bmj.com/site/about/guidelines.xhtml

findings of this evaluation to be of interest to a wide audience given the growing need to demonstrate effectiveness of complex interventions in low resource settings. Findings on the mechanisms and contexts that optimise the implementation of this complex multi-faceted intervention using the ERIC taxonomy will be useful to those developing and implementing these programmes in other health systems. The health economic model may highlight the health expenditure required at individual, family and Kerala State level for forecasting and planning health budgets.

Perhaps the most important aspect of the chosen evaluation is that it is built within a simultaneous developing public health strategy on population-based screening of diabetes and hypertension and a recently introduced eHealth. Conducting research in such an environment is a good example of health policy and systems research.

Despite the limitations, this study holds promise for providing high-quality data and detailed implementation information on a complex intervention in a resources-limited setting. We hope to contribute to the literature on the implementation and effectiveness of DR screening and treatment in the public health sector in LMICs.

Author Contributorship

Sobha Sivaprasad (S.S), Gopalakrishnan Netuveli (G.N), Raphael Wittenberg (R.W), Rajan Khobragade (R.K), Rajeev Sadanandan (R.S), Bipin Gopal (B.G), Lakshmi Premnazir (L.P), Dolores Conroy (D.C), Jyotsna Srinath (J.S), Radha Ramakrishnan (R.R), Simon George (S.G), Vasudeva Iyer Sahasranamam (V.S)

Conceptualisation, S.S, G.N, R.W, R.S, B.G, S.G, V.S; methodology, S.S, G.N, R.R, R.S, B.G, V.S, S.G, D.C, R.R, R.K; acquisition of data, S.S, G.N, R.W, D.C, R.R, J.S, L.P, B.G; writing—original draft preparation, S.S, D.C, G.N, R.W, L.P, R.R; writing—review and editing, S.S, G.N, R.R, R.S, B.G, V.S, S.G, D.C, R.R, R.K, J.S, L.P; funding acquisition, S.S, G.N, R.W, R.S, on behalf of the Collaborators.

Role of study sponsor and funders, if any, in study design; collection, management, analysis, and interpretation of data; writing of the report; and the decision to submit the report for publication, including whether they will have ultimate authority over any of these activities; None

Role of sponsor: The Director of Regional Institute of Ophthalmology contributed to study design; management, writing of the report; and the decision to submit the report for publication, but have no authority over any of these activities.

For peer review only

Authorship eligibility guidelines will be based on ICJME and there is no intention to use professional writers.

Acknowledgements:
Nayanamritham Project Collaborators: Dr Rajeevan Palpoo and Dr Chitra Raghavan will manage the referred patients in Regional Institute of Ophthalmology; Alwin Aniyankunju and Anju V.Molly will be responsible for grading the retinal images.
Steering Committee: Members of the International Advisory Board from Kerala

Funding statement: This work is part of the ORNATE India project funded by Global Challenges Research Fund and UK Research and Innovation through the Medical Research Council grant number MR/P027881/1

Data Sharing Agreement: The technical appendix, statistical code and dataset will be made available on request after obtaining permission from the Government of Kerala.

Competing interests statement: None

Sponsor: Dr V Sahasranamam, Director, Regional Institute of Ophthalmology, Thiruvananthapuram, Kerala

References

1. International Diabetes Federation. *IDF Diabetes Atlas*. 9th edition 2019 https://www.diabetesatlas.org/en/
2. Yau JW, Rogers SL, Kawasaki R, et al. Meta-Analysis for Eye Disease (META-EYE) Study Group. Global prevalence and risk factors of diabetic retinopathy. *Diabetes Care* 2012; 35:556–64.
3. IDF Diabetes Atlas: Global estimates of diabetes prevalence for 2017 and projections for 2045. Cho NH, Shaw JE, Karuranga S, Huang Y, da Rocha Fernandes JD, Ohlrogge AW, Malanda B Diabetes Res Clin Pract. 2018; 138():271-281.
4. Scanlon PH. The English National Screening Programme for diabetic retinopathy 2003-2016. *Acta Diabetol* 2017; 54(6):515-25
5. Cavan D, Makaroff L, da Rocha Fernandes J, et al. The Diabetic Retinopathy Barometer Study: Global perspectives on access to and experiences of diabetic retinopathy screening and treatment. *Diabetes Res Clin Pract* 2017; 129:16-24.
6. NITI Ayog (2020), SDG India Index, Baseline Report, 2018, New Delhi [Last accessed on 2021 Jan 17]. Available from: www.niti.gove.in
7. Menon VU, Kumar KV, Gilchrist A, et al. Prevalence of known and undetected diabetes and associated risk factors in central Kerala--ADEPS. *Diabetes Res Clin Pract* 2006; 74:289–94.

For peer review only - http://bmjopen.bmj.com/site/about/guidelines.xhtml

8. Sustainable Development Goals. United Nations. [Last accessed on 2020 May12]. Available from: http://www.un.org/sustainabledevelopment/sustainable-developmentgoals/

9. The WHO STEPwise approach to Surveillance of noncommunicable diseases (STEPS). Noncommunicable Diseases and Mental Health. World Health Organization. 20 Avenue Appia, 1211 Geneva 27, Switzerland.

10. Scanlon PH, Aldington SJ, Leal J, et al. Development of a cost-effectiveness model for optimisation of the screening interval in diabetic retinopathy screening. *Health Technol Assess* 2015;19(74):1-116.

11. Leese GP, Morris AD, Olson J. A national retinal screening programme for diabetes in Scotland. *Diabet Med* 2003;20(12):962-4.

12. Lund SH, Aspelund T, Kirby P et al. Individualised risk assessment for diabetic retinopathy and optimisation of screening intervals: a scientific approach to reducing healthcare costs. *Br J Ophthalmol*. 2016;100(5):683-7.

13. Manne-Goehler J, Geldsetzer P, Agoudavi K et al. Health system performance for people with diabetes in 28 low- and middle-income countries: A cross-sectional study of nationally representative surveys. *PLoS Med*. 2019;16(3):e1002751.

14. Ting DS, Cheung GC, Wong TY. Diabetic retinopathy: global prevalence, major risk factors, screening practices and public health challenges: a review. *Clin Exp Ophthalmol.* 201;44(4):260-77.

15. Hazin R, Barazi MK, Summerfield M. Challenges to establishing nationwide diabetic retinopathy screening programs. *Curr Opin Ophthalmol*. 2011;22(3):174-9.

16. Burgess PI, Msukwa G, Beare NA. Diabetic retinopathy in sub-Saharan Africa: meeting the challenges of an emerging epidemic. *BMC Med*. 2013;11:157.

17. Ramke J, Petkovic J, Welch V et al. Interventions to improve access to cataract surgical services and their impact on equity in low- and middle-income countries. *Cochrane Database Syst Rev*. 2017;11(11):CD011307.

18. Rani PK, Bhattarai Y, Sheeladevi S et al. Analysis of yield of retinal imaging in a rural diabetes eye care model. *Indian J Ophthalmol*. 2018 Feb;66(2):233-237.

19. Scanlon PH, Foy C, Malhotra R, Aldington SJ. The influence of age, duration of diabetes, cataract, and pupil size on image quality in digital photographic retinal screening. *Diabetes Care*. 2005;28(10):2448-53.

20. Powell BJ, Waltz TJ, Chinman MJ et al. A refined compilation of implementation strategies: results from the Expert Recommendations for Implementing Change (ERIC) project. *Implement Sci*. 2015;10:21.

21. Powell BJ, McMillen JC, Proctor EK, et al. A compilation of strategies for implementing clinical innovations in health and mental health. *Med Care Res Rev*. 2012;69(2):123-157.

For peer review only - http://bmjopen.bmj.com/site/about/guidelines.xhtml

22. Smith JD, Li DH, Rafferty MR. The Implementation Research Logic Model: a method for planning, executing, reporting, and synthesizing implementation projects. *Implement Sci*. 2020 Sep 25;15(1):84.

23. Landes SJ, McBain SA, Curran GM. Reprint of: An introduction to effectiveness-implementation hybrid designs. *Psychiatry Res*. 2020 Jan;283:112630.

24. Rajalakshmi R, Subashini R, Anjana RM, et al. Automated diabetic retinopathy detection in smartphone-based fundus photography using artificial intelligence. *Eye* (Lond) 2018;32(6):1138-1144

25. Piyasena MMPN, Murthy GVS, Yip JLY et al. Systematic review and meta-analysis of diagnostic accuracy of detection of any level of diabetic retinopathy using digital retinal imaging. *Syst Rev*. 2018 Nov 7;7(1):182. doi: 10.1186/s13643-018-0846-y. Erratum in: Syst Rev. 2019 Apr 30;8(1):106.

26. Boucher MC, Nguyen MTD, Qian J. Assessment of Training Outcomes of Nurse Readers for Diabetic Retinopathy Telescreening: Validation Study. *JMIR Diabetes*. 2020;5(2):e17309.

27. Rachapelle S, Legood R, Alavi Y, Lindfield R, Sharma T, Kuper H, Polack S. The cost-utility of telemedicine to screen for diabetic retinopathy in India. *Ophthalmology*. 2013 Mar;120(3):566-573.

28. NIST/SEMATECH e-Handbook of Statistical Methods. Proportion Control Chart https://www.itl.nist.gov/div898/handbook/pmc/section3/pmc332.htm, Accessed 5/1/2021

29. Braun V, Clarke V. What can "thematic analysis" offer health and wellbeing researchers? *Int J Qual Stud Health Well-being*. 2014 Oct 16;9:26152.

30. Becker N, Hakama M, Nyström L. Evaluation of effectiveness of quality-assured mammography screening in Germany: sample size considerations and design options. *Eur J Cancer Prev* 2007;16(3):225-31.

Legend: Figure 1: Proposed Diabetic Retinopathy Care Pathway

Figure 2: The Implementation Research Logic Model

For peer review only - http://bmjopen.bmj.com/site/about/guidelines.xhtml

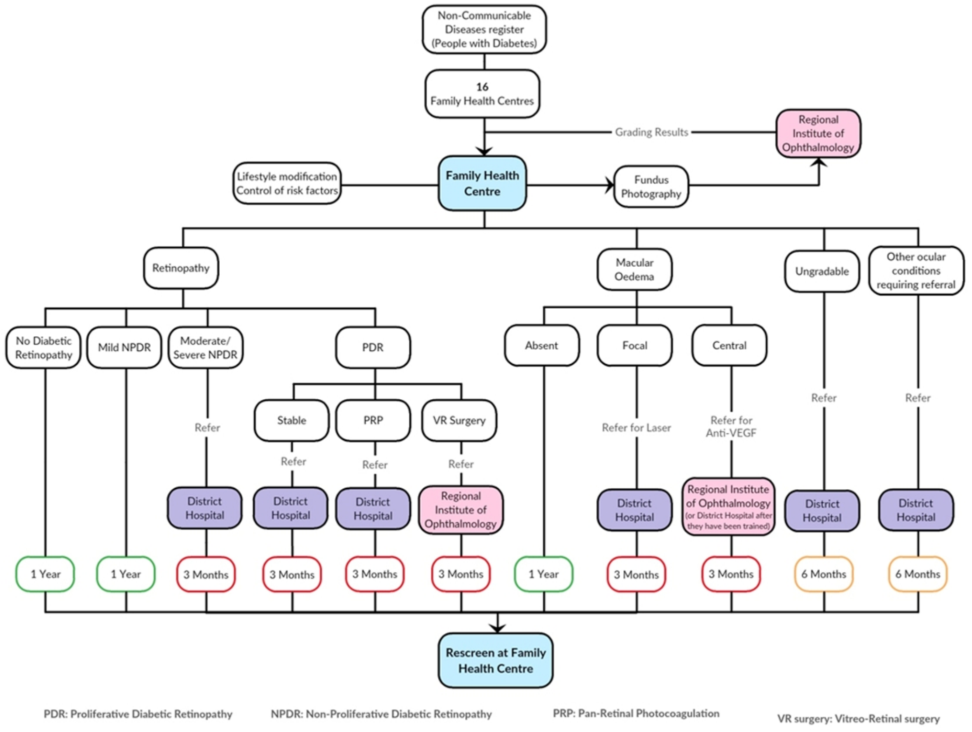

Figure !

186x138mm (300 x 300 DPI)

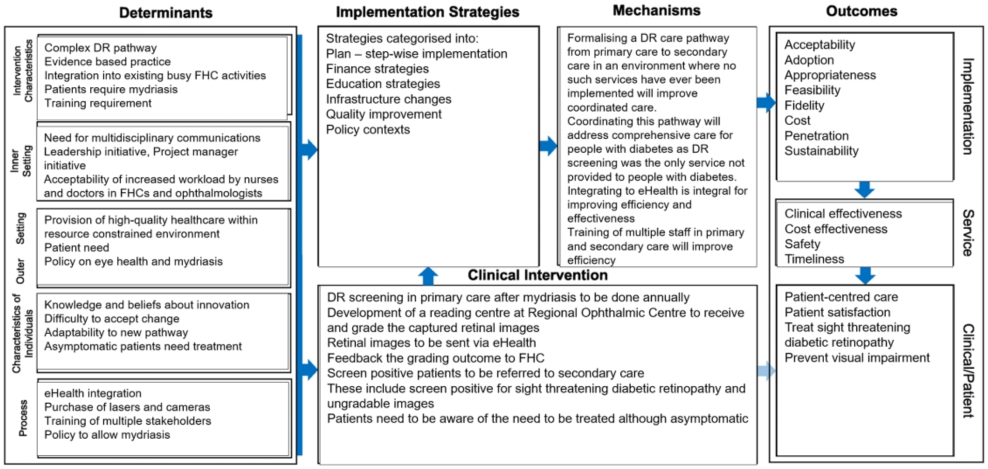

Figure 2 The Implementation Research Logic Model

188x90mm (300 x 300 DPI)

STANDARD PROTOCOL ITEMS: RECOMMENDATIONS FOR INTERVENTIONAL TRIALS

SPIRIT 2013 Checklist: Recommended items to address in a clinical trial protocol and related documents*

Section/item	Item No	Description	Addressed on page number
**Administrative information**			
Title	1	Descriptive title identifying the study design, population, interventions, and, if applicable, trial acronym	_1____________
Trial registration	2a	Trial identifier and registry name. If not yet registered, name of intended registry	__1___________
	2b	All items from the World Health Organization Trial Registration Data Set	__NA__________
Protocol version	3	Date and version identifier	_1____________
Funding	4	Sources and types of financial, material, and other support	__24______
Roles and responsibilities	5a	Names, affiliations, and roles of protocol contributors	__25__________
	5b	Name and contact information for the trial sponsor	__25__________
	5c	Role of study sponsor and funders, if any, in study design; collection, management, analysis, and interpretation of data; writing of the report; and the decision to submit the report for publication, including whether they will have ultimate authority over any of these activities	__25__________
	5d	Composition, roles, and responsibilities of the coordinating centre, steering committee, endpoint adjudication committee, data management team, and other individuals or groups overseeing the trial, if applicable (see Item 21a for data monitoring committee)	__18_________

For peer review only - http://bmjopen.bmj.com/site/about/guidelines.xhtml

Introduction

Background and rationale	6a	Description of research question and justification for undertaking the trial, including summary of relevant studies (published and unpublished) examining benefits and harms for each intervention	__4___________
	6b	Explanation for choice of comparators	__6___________
Objectives	7	Specific objectives or hypotheses	__6__________
Trial design	8	Description of trial design including type of trial (eg, parallel group, crossover, factorial, single group), allocation ratio, and framework (eg, superiority, equivalence, noninferiority, exploratory)	___6__________

Methods: Participants, interventions, and outcomes

Study setting	9	Description of study settings (eg, community clinic, academic hospital) and list of countries where data will be collected. Reference to where list of study sites can be obtained	___7__________
Eligibility criteria	10	Inclusion and exclusion criteria for participants. If applicable, eligibility criteria for study centres and individuals who will perform the interventions (eg, surgeons, psychotherapists)	____6________
Interventions	11a	Interventions for each group with sufficient detail to allow replication, including how and when they will be administered	___7__________
	11b	Criteria for discontinuing or modifying allocated interventions for a given trial participant (eg, drug dose change in response to harms, participant request, or improving/worsening disease)	____7-8_______
	11c	Strategies to improve adherence to intervention protocols, and any procedures for monitoring adherence (eg, drug tablet return, laboratory tests)	_____18________
	11d	Relevant concomitant care and interventions that are permitted or prohibited during the trial	_____NA_______ _
Outcomes	12	Primary, secondary, and other outcomes, including the specific measurement variable (eg, systolic blood pressure), analysis metric (eg, change from baseline, final value, time to event), method of aggregation (eg, median, proportion), and time point for each outcome. Explanation of the clinical relevance of chosen efficacy and harm outcomes is strongly recommended	___table 2 and 3__

For peer review only - http://bmjopen.bmj.com/site/about/guidelines.xhtml

Participant timeline	13	Time schedule of enrolment, interventions (including any run-ins and washouts), assessments, and visits for participants. A schematic diagram is highly recommended (see Figure)	_____Figure 1
Sample size	14	Estimated number of participants needed to achieve study objectives and how it was determined, including clinical and statistical assumptions supporting any sample size calculations	_18____________
Recruitment	15	Strategies for achieving adequate participant enrolment to reach target sample size	__table 1___

Methods: Assignment of interventions (for controlled trials)

Allocation:

Sequence generation	16a	Method of generating the allocation sequence (eg, computer-generated random numbers), and list of any factors for stratification. To reduce predictability of a random sequence, details of any planned restriction (eg, blocking) should be provided in a separate document that is unavailable to those who enrol participants or assign interventions	_____________
Allocation concealment mechanism	16b	Mechanism of implementing the allocation sequence (eg, central telephone; sequentially numbered, opaque, sealed envelopes), describing any steps to conceal the sequence until interventions are assigned	_____________
Implementation	16c	Who will generate the allocation sequence, who will enrol participants, and who will assign participants to interventions	_____________
Blinding (masking)	17a	Who will be blinded after assignment to interventions (eg, trial participants, care providers, outcome assessors, data analysts), and how	_____________
	17b	If blinded, circumstances under which unblinding is permissible, and procedure for revealing a participant's allocated intervention during the trial	_____________

Methods: Data collection, management, and analysis

Data collection methods	18a	Plans for assessment and collection of outcome, baseline, and other trial data, including any related processes to promote data quality (eg, duplicate measurements, training of assessors) and a description of study instruments (eg, questionnaires, laboratory tests) along with their reliability and validity, if known. Reference to where data collection forms can be found, if not in the protocol	_____17________

	18b	Plans to promote participant retention and complete follow-up, including list of any outcome data to be collected for participants who discontinue or deviate from intervention protocols	____8-12______
Data management	19	Plans for data entry, coding, security, and storage, including any related processes to promote data quality (eg, double data entry; range checks for data values). Reference to where details of data management procedures can be found, if not in the protocol	_______17-18__
Statistical methods	20a	Statistical methods for analysing primary and secondary outcomes. Reference to where other details of the statistical analysis plan can be found, if not in the protocol	____19_________
	20b	Methods for any additional analyses (eg, subgroup and adjusted analyses)	______19_______
	20c	Definition of analysis population relating to protocol non-adherence (eg, as randomised analysis), and any statistical methods to handle missing data (eg, multiple imputation)	____NA________ _
**Methods: Monitoring**			
Data monitoring	21a	Composition of data monitoring committee (DMC); summary of its role and reporting structure; statement of whether it is independent from the sponsor and competing interests; and reference to where further details about its charter can be found, if not in the protocol. Alternatively, an explanation of why a DMC is not needed	____18________
	21b	Description of any interim analyses and stopping guidelines, including who will have access to these interim results and make the final decision to terminate the trial	____NA________ _
Harms	22	Plans for collecting, assessing, reporting, and managing solicited and spontaneously reported adverse events and other unintended effects of trial interventions or trial conduct	_____NA_______ _
Auditing	23	Frequency and procedures for auditing trial conduct, if any, and whether the process will be independent from investigators and the sponsor	____NA________ _
**Ethics and dissemination**			
Research ethics approval	24	Plans for seeking research ethics committee/institutional review board (REC/IRB) approval	______20______

Protocol amendments	25	Plans for communicating important protocol modifications (eg, changes to eligibility criteria, outcomes, analyses) to relevant parties (eg, investigators, REC/IRBs, trial participants, trial registries, journals, regulators)	___1__________
Consent or assent	26a	Who will obtain informed consent or assent from potential trial participants or authorised surrogates, and how (see Item 32)	____20_________
	26b	Additional consent provisions for collection and use of participant data and biological specimens in ancillary studies, if applicable	____NA________ _
Confidentiality	27	How personal information about potential and enrolled participants will be collected, shared, and maintained in order to protect confidentiality before, during, and after the trial	_____18________
Declaration of interests	28	Financial and other competing interests for principal investigators for the overall trial and each study site	___25__________
Access to data	29	Statement of who will have access to the final trial dataset, and disclosure of contractual agreements that limit such access for investigators	____25_________
Ancillary and post-trial care	30	Provisions, if any, for ancillary and post-trial care, and for compensation to those who suffer harm from trial participation	____NA________ _
Dissemination policy	31a	Plans for investigators and sponsor to communicate trial results to participants, healthcare professionals, the public, and other relevant groups (eg, via publication, reporting in results databases, or other data sharing arrangements), including any publication restrictions	____20_________
	31b	Authorship eligibility guidelines and any intended use of professional writers	____25_________
	31c	Plans, if any, for granting public access to the full protocol, participant-level dataset, and statistical code	___25_________
**Appendices**			
Informed consent materials	32	Model consent form and other related documentation given to participants and authorised surrogates	_______suppleme ntary document_
Biological specimens	33	Plans for collection, laboratory evaluation, and storage of biological specimens for genetic or molecular analysis in the current trial and for future use in ancillary studies, if applicable	________NA____ _

For peer review only

*It is strongly recommended that this checklist be read in conjunction with the SPIRIT 2013 Explanation & Elaboration for important clarification on the items. Amendments to the protocol should be tracked and dated. The SPIRIT checklist is copyrighted by the SPIRIT Group under the Creative Commons "Attribution-NonCommercial-NoDerivs 3.0 Unported" license.

For peer review only

BMJ Open

Complex interventions to implement a diabetic retinopathy care pathway in the public health system in Kerala: the Nayanamritham study protocol.

Journal:	*BMJ Open*
Manuscript ID	bmjopen-2020-040577.R3
Article Type:	Protocol
Date Submitted by the Author:	03-Mar-2021
Complete List of Authors:	Sivaprasad, Sobha; UCL, NIHR Moorfields Biomedical Research; Moorfields Eye Hospital NHS Foundation Trust<br>Netuveli, Gopalakrishnan; University of East London - Duncan House Campus, Institute of Connected Communities<br>Wittenberg, Raphael; Oxford University, Nuffield Department of Primary Health Care Sciences<br>Khobragade, Rajan; Government of Kerala<br>Sadanandan, Rajeev; Government of Kerala<br>Gopal, Bipin; Government Medical College Thiruvananthapuram, Directorate of Health Services<br>Premnazir, Lakshmi; Government Medical College Thiruvananthapuram, Directorate of Health Services<br>Conroy, Dolores; UCL, Institute of Ophthalmology<br>Srinath, Jyotsna; University of East London - Duncan House Campus, Department of connected communities<br>Ramakrishnan, Radha; UCL, Institute of Ophthalmology<br>George, Simon; Government Medical College Thiruvananthapuram, Regional Institute of Ophthalmology<br>Sahasranamam, Vasudeva Iyer ; Government Medical College Thiruvananthapuram, Regional Institute of Ophthalmology
**Primary Subject Heading**:	Ophthalmology
Secondary Subject Heading:	Public health, Diabetes and endocrinology
Keywords:	Diabetic retinopathy < DIABETES & ENDOCRINOLOGY, General diabetes < DIABETES & ENDOCRINOLOGY, Protocols & guidelines < HEALTH SERVICES ADMINISTRATION & MANAGEMENT

SCHOLARONE™
Manuscripts

I, the Submitting Author has the right to grant and does grant on behalf of all authors of the Work (as defined in the below author licence), an exclusive licence and/or a non-exclusive licence for contributions from authors who are: i) UK Crown employees; ii) where BMJ has agreed a CC-BY licence shall apply, and/or iii) in accordance with the terms applicable for US Federal Government officers or employees acting as part of their official duties; on a worldwide, perpetual, irrevocable, royalty-free basis to BMJ Publishing Group Ltd ("BMJ") its licensees and where the relevant Journal is co-owned by BMJ to the co-owners of the Journal, to publish the Work in this journal and any other BMJ products and to exploit all rights, as set out in our licence.

The Submitting Author accepts and understands that any supply made under these terms is made by BMJ to the Submitting Author unless you are acting as an employee on behalf of your employer or a postgraduate student of an affiliated institution which is paying any applicable article publishing charge ("APC") for Open Access articles. Where the Submitting Author wishes to make the Work available on an Open Access basis (and intends to pay the relevant APC), the terms of reuse of such Open Access shall be governed by a Creative Commons licence – details of these licences and which Creative Commons licence will apply to this Work are set out in our licence referred to above.

Other than as permitted in any relevant BMJ Author's Self Archiving Policies, I confirm this Work has not been accepted for publication elsewhere, is not being considered for publication elsewhere and does not duplicate material already published. I confirm all authors consent to publication of this Work and authorise the granting of this licence.

For peer review only

Complex interventions to implement a diabetic retinopathy care pathway in the public health system in Kerala: the Nayanamritham study protocol.

Sobha Sivaprasad[1, 2], Gopalakrishnan Netuveli[3], Raphael Wittenberg[4], Rajan Khobragade[5], Rajeev Sadanandan[5], Bipin Gopal[6], Lakshmi Premnazir[6], Dolores Conroy[2], Jyotsna Srinath[3], Radha Ramakrishnan[2], Simon George[7], Vasudeva Iyer Sahasranamam[7] for the Nayanamritham Project Collaborators.

1. Medical Retina Department, NIHR Biomedical Research Centre, Moorfields Eye Hospital NHS Foundation Trust and University College London, London, UK
2. Department of Vision Sciences, UCL Institute of Ophthalmology, London, UK
3. Institute of Connected Communities, University of East London, UK
4. Nuffield Department of Primary Health Care Sciences, University of Oxford, Oxford, UK
5. Directorate of Health, Government of Kerala, India
6. Non Communicable Diseases Department, Directorate of Health Services, Kerala, India
7. Ophthalmology Department, Regional Institute of Ophthalmology, Thiruvananthapuram, Kerala, India

Word count: 3696

Figures: 2

Table: 3

References: 31

Abstract: 295

Corresponding author

Professor Sobha Sivaprasad
NIHR Moorfields Biomedical Research Centre
Moorfields Eye Hospital NHS Foundation Trust,
162 City Road,
London EC1V 2PD, UK
Tel: 020 7253 3411
Email: senswathi@aol.com

Keywords: diabetic retinopathy, implementation study, Kerala, laser photocoagulation, diabetes, screening, visual impairment, cost-effectiveness.

Trial Registration Number: ISRCTN28942696

Protocol Version: 1.0 dated 13-03-2019

For peer review only

Abstract

Introduction: This mixed methods study aims to pilot a diabetic retinopathy (DR) care pathway in the public health system in Thiruvananthapuram district in Kerala to understand how it can be scaled up to and sustained in the whole state.

Methods and analysis: Currently, there is no systematic DR screening programme in Kerala. Our intervention is a teleophthalmology pathway for people with diabetes in the non-communicable disease (NCDs) registers in 16 family health centres (FHCs). The planned implementation strategy of the pathway will be developed based on the discrete Expert Recommendations for Implementing Change (ERIC) taxonomy. We will utilise both quantitative data from a cross-sectional study and qualitative data obtained from structured interviews, surveys and group discussions with stakeholders to report the effectiveness of the DR care pathway and evaluation of the implementation strategy.

The primary effectiveness outcome is the proportion of patients in the NCD register with diabetes screened for DR by 12 months. Other outcomes include safety, efficiency, patient satisfaction, timeliness and equity. The key cost-effectiveness outcome will be the number of cases of severe visual impairment or blindness due to sight threatening DR averted as a result of the DR care pathway. The outcomes of evaluation of the implementation strategies include acceptability, feasibility, adoption, appropriateness, fidelity, penetration, costs and sustainability. Addition of more FHCs during the staggered initial phase of the programme will be considered as a sign of acceptability and feasibility. In the long term, the state-wide adoption of the DR care pathway will be considered as a successful outcome of the Nayanamritham study.

Ethics and Dissemination: The study was approved by Indian Medical Research Council (2018-0551) dated 13/03/2019. Study findings will be disseminated through scientific publications and the report will inform adoption of the DR care pathway by Kerala state in future.

For peer review only - http://bmjopen.bmj.com/site/about/guidelines.xhtml

ARTICLE SUMMARY

- This study will examine the clinical and cost effectiveness of a new diabetic retinopathy care pathway at the patient, clinician and service levels and evaluate the implementation strategy within a resource constrained environment.
- The study will use a mixed-methods approach.
- The specific actions in the implementation strategy are based on the Expert Recommendations for Implementing Change (ERIC) taxonomy
- The study outlines the economic evaluation of the costs of the diabetic retinopathy care pathway**.**
- The study is limited by the absence of a comparator due to lack of previous data on the prevalence of diabetic retinopathy in the public health system.

For peer review only

INTRODUCTION

Background

The triad of diabetes, blindness and poverty is an urgent problem that needs an effective response in the Development Assistance Committee-listed countries as these countries are home to 80% of people with diabetes. [1] Diabetic retinopathy (DR) is one of the most common complications of diabetes. [2] Sight-threatening diabetic retinopathy (STDR) is a common cause of blindness in working-age people and unless this condition is managed early, it has considerable impact on the quality of life and productivity of the person and their family, as well as substantial financial costs to health systems. [3] In its early stages, STDR may be asymptomatic. Therefore, DR screening programmes are essential to identify STDR to enable timely treatment. [4] In DR screening programmes in high income countries, people with diabetes are systematically screened using office-based retinal cameras and the retinal images are graded according to severity of DR by dedicated accredited screeners and graders. Patients with STDR are identified and referred to ophthalmologists for timely treatment with laser and/or intravitreal injections of anti-vascular endothelial growth factor. [4] In order to successfully reduce the risk of blindness due to DR in low-middle income countries (LMICs), without a well-developed primary care infrastructure, the pathway needs to begin with systematically screening for diabetes, educating the public and healthcare professionals on early detection[5] and timely treatment of STDR and the need to optimally treat the risk factors for DR such as hyperglycaemia, hypertension and hyperlipidemia. [2] Moreover, DR screening and treatment are challenging due to the required technology and technical expertise needed to grade retinal images and deliver costly treatment options, adding to the cost and complexity of the required interventions.

Diabetes in Kerala

Kerala is the most advanced state in India in terms of literacy, health, social uplift and demographic transition.[6] This has been accompanied by high prevalence of diabetes. A recent report from Kerala suggests that one in five of the Kerala adult population may have diabetes.[7] The Government of Kerala launched the Aardram Mission in 2017 to transform and gear up the State's public health care system to achieve the Sustainable Development Goals (SDGs) in phases with short term goals on building infrastructure and quality care services. The overarching objectives of the Aardram Mission included providing equitable, affordable and quality care to citizens from all socio-economic strata; strengthening the public care system by decentralising healthcare from the secondary and tertiary level to primary care-led services and

For peer review only - http://bmjopen.bmj.com/site/about/guidelines.xhtml

For peer review only

initiating preventive medicine to address the impact of NCDs, especially hypertension and diabetes. Primary care centres have been converted to family health centres (FHCs) with three doctors and four nurses in each FHC assigned to provide individual care plans to the allocated population whose records are tracked through a recently established Electronic Health Records (EHR) called eHealth. The transformation of primary care with a focus on NCDs provided the backdrop to the implementation of a DR care pathway.

The rationale for a complex DR care pathway

The Government of Kerala is pressing forward to achieve universal health coverage and address the SDG goals on poverty (SDG 1), health access (SDG 3), education (SDG 4) and gender equality (SDG 5). [8, 9] Therefore, there is an urgent need to tackle the complications of diabetes. Systematic DR screening has been shown to be effective in reducing blindness. [3] However, in high income countries, DR screening is achieved by dedicated services due to the technicalities and expertise required in DR screening and evaluation. [10-12] Introducing an isolated DR care pathway will not be sufficient to address the challenge in LMICs as these countries need to initiate holistic screening service for diabetes and all its complications simultaneously and embed DR screening within the primary care.

Rationale for an Implementation Strategy

Each aspect of the DR screening and care pathway has to be adapted to local needs and resources, requiring a locally appropriate implementation strategy. For example, mydriasis (pupil dilatation) is compulsory in some screening programmes but not in others and this process requires local approval by stakeholders. [10-12] In Kerala, mydriasis needs to be approved by the health department. Other issues faced by LMIC are that the number of undiagnosed diabetes cases is high, [13] the non-availability of a state-wide diabetes register with recall facilities, the lack of resources for standardised non-portable retinal cameras, limited capacity of staff in primary care to screen for DR, and that the number of people with diabetes is larger than the capacity of the services provided by the secondary care hospitals. [14-16] Research capacity and capability are in their infancy in LMICs and implementing change in a busy environment when demands on staff are great and resources are limited is challenging. [15, 16] In addition, as cataract is more prevalent in LMIC, [17] the proportion of ungradable retinal images is higher compared to developed countries. [17-19] Therefore, we need to evaluate the implementation strategy using both qualitative and quantitative methods. [20-22]

For peer review only - http://bmjopen.bmj.com/site/about/guidelines.xhtml

Aims and Objectives

The Nayanamritham study is facilitated by a UK-Government of Kerala partnership as a part of the ORNATE-India project funded by Global Challenges Research Fund (GCRF) and UK Research and Innovation (UKRI). We aim to introduce a DR care pathway that spans primary, secondary and tertiary care in the public health system in Kerala in a pilot study in Thiruvananthapuram (Figure 1). The proposed study will (1) examine the clinical and cost effectiveness of the DR care pathway at the patient, clinician and service levels and (2) evaluate the implementation strategy of the pathway.

METHODS

Design: We chose a mixed-methods approach to evaluate the effectiveness of the clinical interventions and the implementation strategies. [20-23] The DR care pathway was developed by the Kerala Health Secretary, NCD lead, Health and Medical Education service providers, technical and EHR teams, local authorities and the GCRF/UKRI- funded co-applicants from the UK. The DR care pathway will be set-up in a staggered approach with 5 FHCs initiated in the first three months and the remaining 11 FHCs will be added based on acceptance of all stakeholders and adapting and training phase for 12 months from 15-03-2018. A further 12-15 months will be allocated to recruit consenting patients to a cross-sectional study to gather quantitative data for the effectiveness outcomes. A minimal dataset from this study will also be entered into EHR to enable future screening for the consented patients. Qualitative data collection from interviews of staff, patients and focus groups at baseline and end of study and all field notes gathered during the study will be utilised to inform evaluation of the implementation strategy. [20-22]

Target Population

People with diabetes registered in the NCD register at 16 FHCs in Thiruvananthapuram district will be invited to be screened for complications of diabetes including screening for DR. As the NCD register is likely to show an increase in newly diagnosed diabetes as a result of training the accredited social health activists (ASHAs), in diabetes and DR, we will evaluate the effectiveness of the complex interventions delivered at each of the 16 FHCs for this study. The implementation of the pathway will take into account all patients in the NCD register at the start of the cross-sectional study.

For peer review only - http://bmjopen.bmj.com/site/about/guidelines.xhtml

Setting

Target sites for implementation of DR screening and treatment in Thiruvananthapuram will include 16 FHCs for DR screening which represents the primary care centres where patients are screened for all complications of diabetes. The retinal images, captured by the trained, resident nursing staff will be sent to a newly developed reading centre at the Regional Institute of Ophthalmology (RIO), a tertiary care centre, where newly accredited graders will grade the images. Patients with screen-positive images will be referred to three secondary care hospitals (district hospitals). Severe cases that require complex interventions will be referred to the tertiary care centre, the RIO for specialist management of DR.

Description of the standard of care

Currently, patients with diabetes are not systematically screened for DR in the public health system. Most patients present to the tertiary centre voluntarily either because of increased awareness of complications of diabetes or due to visual impairment. Therefore, the current standard of care will be captured as the number of patients presenting to the tertiary centre for an eye consultation for DR over a period of time as there is no baseline data in the primary and secondary care. This cross-sectional survey of the patients presenting at the FHCs will provide information on the uptake of screening of people registered in the NCD register at the start of the study. The prevalence of DR and STDR of the screened population will be estimated from the numbers screened in all the NCD registers during this period.

Evidence-based clinical intervention:

The new DR care pathway is the intervention and is shown in Figure 1. The pathway will span primary, secondary and tertiary care. The components of the pathway are:

1. DR screening of patients with diabetes registered in the NCD register at FHCs (primary care). The retinal images will be graded remotely at the reading centre at RIO.
2. Prompt referral for timely treatment of STDR to secondary care and tertiary centres depending on the severity of the DR.
3. Treatment of patients with sight threatening DR (at secondary and tertiary care).

Implementation strategies

The implementation strategies of the new DR care pathway are categorised as shown in Table 1 into plan, finance, education, infrastructure, quality improvement and policy contexts. These

For peer review only - http://bmjopen.bmj.com/site/about/guidelines.xhtml

categories are developed based on the discrete Expert Recommendations for Implementing Change (ERIC) taxonomy.[20, 21] The logic model is shown in Figure 2.

Table 1: Implementation strategies

Categories of Implementation Strategies[21]	Discrete Implementation Strategy[20]	Specific actions
Plan	Conduct local needs assessment	The study team will conduct a situational analysis of data from self-referred patients. This will serve as the comparator for the effectiveness outcome.
	Assess for readiness and identify barriers and facilitators	Assess barriers that may impede implementation of the DR care pathway, and strengths that can be used in the implementation effort by interviewing key stakeholders i.e. FHC staff, ophthalmologists in secondary and tertiary care, health service employees and patients. This will contribute to the implementation outcome in terms of acceptability and appropriateness of the programme.
	Develop a formal implementation blueprint	The blueprint will be used to guide the implementation of the whole DR care pathway and will be updated with time. This will be utilised to evaluate the feasibility and fidelity of the programme
	Tailor strategies	Regular collaborative meetings will be held to tailor the implementation strategies to address barriers and leverage facilitators that are identified during various steps in the implementation. This will contribute to the feasibility and fidelity outcomes of the implementation strategy
	Stage implementation scale up and integration	The implementation will be staggered with five FHCs starting as pilots to enable small changes before gradually moving to other 11 FHCs. Addition of more FHCs during the staggered phase will be considered as sign of acceptability and feasibility.<br>Ensure integration of the DR care pathway into EHR. In the long-term, the adoption of the DR care pathway across the state of Kerala will be considered as success.
	Build a coalition	Cultivate relationships with staff at FHCs and secondary care centres, data entry operators, EHR team, project manager, and

For peer review only

		the Government of Kerala health service departmental staff in the implementation effort and create a learning system to incorporate practitioner feedback. This will be utilised to report the appropriateness and adoption outcomes.
	Develop academic partnerships	Partner with UK academic institutions for the purposes of shared training and bringing research skills to the implementation of the pathway. This will contribute to the acceptability and adoption outcomes
	Recruit, designate, and train for leadership	A project manager will be recruited to manage the programme under the supervision of the NCD lead. The Health Secretary will conduct periodic meetings with the project team to assess performance. This will contribute to the penetration outcome.
	Obtain formal commitments	A collaborative agreement is in place between Government of Kerala and Moorfields Eye Hospital, London, UK, outlining their roles and responsibilities in the implementation of the DR care pathway. This will contribute to acceptability, adoption and sustainability outcomes
	Prepare patients to be active participants	Community level health workers will prepare patients to be active in their care, to ask questions, and specifically to inquire about care guidelines, the evidence behind clinical decisions, or about available evidence-supported treatments. Each patient will provide written consent to be screened for DR in this project as well as participate in interviews after verbal consent. This will provide data for effectiveness and feasibility outcomes.
	Inform local opinion leaders	The Health Minister of the Kerala state and members of the local government will be briefed on the project and encouraged to be champions of the project to enable scale up to all FHCs in the state. This will contribute to the feasibility and sustainability outcomes.
Finance	Access new funding	Use GCRF/UKRI funding to purchase smartphone retinal cameras and retinal lasers, develop EHR, train staff at FHCs and secondary care, employ project manager, data entry operators and personnel to conduct interviews, create study database linked to hand-held applications for data entry operators. State and local government

For peer review only

		funds will be sourced for scale up. This will contribute to the economic evaluation.
	Incentivisation	ASHAs will be incentivised to accompany participants with diabetes who are referred to secondary care for treatment. This will contribute to the economic evaluation.
Education	Develop educational materials	Develop DR training modules and retinal image capture modules for FHC staff Develop laser training material to train ophthalmologists. This will contribute to the adoption and fidelity outcomes
	Distribute educational materials	Distribute educational materials (including guidelines, manuals, and toolkits) in person, by mail, and/or electronically. Multiple training sessions are planned at each FHC. The camera manufacturers will be invited to provide hands-on training on capturing good quality retinal images. Training on laser surgery will also be provided to the ophthalmologists at secondary care. This will contribute to the adoption and fidelity outcomes
	Conduct educational meetings	Hold meetings targeting different stakeholder groups (ophthalmologists, FHC staff, ASHAs, patients in FHCs). Three educational meetings are planned by the UK team to meet with the providers in the FHC settings to share knowledge and educate providers about the DR care pathway and its integration into their clinical practice. This will contribute to acceptability and adoption outcomes.
	Achieve accreditation for graders of retinal images	Train and certify the optometrists in the Reading Centre in RIO to obtain accreditation as graders using the online international Test and Training for retinal graders. This will contribute to acceptability adoption and fidelity outcomes.
	Conduct ongoing training	Nurses and doctors in the FHCs will receive on-going training on DR, pupil dilatation and capture of retinal images using low-cost smartphone retinal cameras.[24, 25] This will contribute to acceptability adoption and fidelity outcomes.
	Create a learning collaborative	The Health Secretary of State will foster a collaborative learning environment to improve implementation of the DR care pathway. This will contribute to the adoption, penetration and sustainability outcome.

For peer review only - http://bmjopen.bmj.com/site/about/guidelines.xhtml

	Use train-the-trainer strategies	The UK team will first train a batch of FHC nurses to capture retinal images and then some will be trained to train the rest of the nurses. The same cascading pathway will apply to laser training. This will be utilised to inform the adoption and fidelity outcomes.
	Work with educational institutions	Moorfields Eye Hospital staff will provide the necessary guidance and share knowledge on UK diabetic retinopathy screening pathway. This will be utilised to inform the adoption and fidelity outcomes.
	Make training dynamic	All staff involved will receive continual online or face to face training. This will be utilised to inform the acceptability, adoption, fidelity and penetration outcomes.
	Increase demand	The ASHAs will be trained to increase public awareness of this pathway in their house-to-house health visits. This will inform the appropriateness and acceptability outcomes of the programme
	Provide ongoing consultation	The UK team from Moorfields Eye Hospital will provide ongoing consultation to support implementation of DR care pathway. The data will be used to inform the appropriateness and fidelity outcomes.
Infrastructure	Centralize technical assistance	The project manager and the EHR team will provide central assistance focused on implementation issues. The telemedicine project will link the patient's retinal images to EHR and transfer through a secure cloud to RIO, the tertiary centre, where the images will be graded for their DR status in a newly developed reading centre, with the results fed back to the FHCs.[26] This will inform patient safety, efficiency, fidelity and feasibility outcomes.
	Change physical structure and equipment	Evaluate current configuration of FHC consulting rooms to allow suitable furniture for cameras and adapt illumination to capture retinal photographs. This will inform integration, adoption, feasibility and fidelity of the implementation strategy and efficiency outcomes.
	Change record systems	Change records systems to allow better assessment of implementation or clinical outcomes by having data entry operators interview patients and input full data into EHR within the busy FHC clinics. This will enable integration and contribute to the

For peer review only - http://bmjopen.bmj.com/site/about/guidelines.xhtml

		effectiveness, efficiency and fidelity outcomes.
	Change service sites	Change the timetable for clinics in secondary care to allow a patients referred from primary care to access treatment. This will contribute to the timeliness, fidelity and feasibility outcomes
	Revise professional roles	The NCD lead will be delegated to take on the responsibility of implementing this DR care pathway in Thiruvananthapuram aiming for a Kerala state-wide roll out. This will contribute to the penetration and sustainability outcomes
Quality Improvement	Develop and implement tools for quality monitoring	Develop, test, and introduce into quality-monitoring systems the right input—the appropriate language for the consent form, protocols on DR screening, grading retinal images, and measures of processes, patient outcomes, and implementation outcomes. This will contribute to both effectiveness and implementation outcomes.
	Develop and organize quality monitoring systems	Develop and organise systems and procedures that monitor clinical processes and/or outcomes for the purpose of quality assurance and improvement. In addition to integration, it will contribute to both implementation outcomes.
	Audit and provide feedback	Collect and summarise clinical performance data every month and feedback to FHCs and secondary care staff to monitor, evaluate, and modify pathway for acceptability to patients and staff. A continuous monitoring of retinal image quality and gradability, and the referral pathway will be done to cyclically input change into the DR pathway to improve the quality and integration. The data will contribute to both effectiveness and implementation outcomes.
	Conduct cyclical small tests of change	Staggered set up of FHCs to allow implementation of changes in a cyclical fashion using small tests of change before taking changes system-wide. Tests of change benefit will be measured continually by increase uptake of training, screening, referral and treatment. This will contribute to the efficiency, timeliness, feasibility and fidelity outcomes.
	Use data experts	The EHR team will develop an application for the data entry operators to collect data. The UK collaborators will collaborate in the

For peer review only - http://bmjopen.bmj.com/site/about/guidelines.xhtml

		analysis of the data. This will contribute to effectiveness, safety, adoption
	Use data warehousing techniques	Integrate clinical records from this research project into EHR on an on-going basis. This will provide data on integration, penetration, adoption and sustainability.
	Capture and share local knowledge	Capture local knowledge from implementation sites on how staff and doctors make something work in their FHCs and then share it with other FHCs. This will contribute to adoption, penetration, acceptability and feasibility.
	Obtain and use patients feedback	The participating patients will be asked to provide feedback on the implementation effort. This will provide data on patient satisfaction, equity, adoption and penetration.
	Promote adaptability	Identify way the DR care pathway can be tailored to meet local needs and clarify which elements of the pathway must be maintained to preserve fidelity. As an example, should patients be screened daily or should they be allocated to a single weekly clinic to improve efficiency? This will provide data for fidelity and feasibility outcomes.
	Provide clinical supervision	The Director of the RIO will provide the overall clinical supervision of the whole DR care pathway. He will also delegate ophthalmologists to provide ongoing supervision for the optometrists involved in grading retinal images. This will contribute to the effectiveness, timeliness, safety outcomes.
	Provide local technical assistance	Develop and use a system to deliver technical assistance focused on implementation issues using relevant service providers. This will provide data for adoption, feasibility and appropriateness outcomes.
	Purposely re-examine the implementation	Monthly monitoring of screened patients at each FHCs, barriers observed and small changes that are required will be implemented to continuously improve the pathway. This will contribute to the adoption, acceptability, feasibility and fidelity outcomes.
	Remind clinicians	Primary care doctors in FHCs will receive monthly newsletter on the numbers recruited across all FHCs to highlighting successes and providing potential solutions to issues

For peer review only - http://bmjopen.bmj.com/site/about/guidelines.xhtml

		encountered. This will contribute to the adoption and penetration outcomes.
Policy contexts	Issue Government of Kerala approval to enable dilatation of pupils of patients in FHC under supervision of FHC doctors	Ensure doctors at FHCs receive approval for mydriasis in order to deliver the DR care pathway. This will contribute to the feasibility, adoption and sustainability outcomes.
	Mandate change	The Health Secretary of State will provide leadership to ensure the prioritisation of the implementation of the DR care pathway. This will contribute to the adoption, penetration and sustainability outcomes.

FHCs -family health centres; DR-diabetic retinopathy; NCD-non-communicable diseases; ASHAs -accredited social health activists; GCRF/UKRI -Global Challenge Research Fund/UK Research and Innovation; RIO –Regional Institute of Ophthalmology, EHR –electronic health records.

Outcomes

Pre-specified outcomes of the effectiveness of the DR pathway and the evaluation of the implementation strategy are tabulated in table 2 and table 3, respectively.

Table 2: DR pathway- (intervention) related outcomes

Outcomes	Indicators	Source of data
Clinical Effectiveness	– Proportion of people screened for DR in each FHC over 12 months.<br>– Proportion of referred patients with STDR.<br>– Proportion of ungradable retinal images and percentage of cataract identified from referring these patients.<br>– Risk and complication burden in people screened for DR<br>– Presenting visual acuity of those referred from the DR pathway compared to those who self-referred to secondary care.<br>– Generalisability of the prevalence data compared to house-to-house survey in a neighbouring district.	Data from monthly entries into EHR and study database.

For peer review only

Safety	Data on complications of the DR pathway	EHR and study database
Cost-effectiveness	The key outcome is the number of cases of severe visual impairment or blindness due to STDR averted as a result of the new DR care pathway and resultant QALY gain.<br>Estimated QALY gain from laser treatment of STDR and from cataract surgery, using utility values from Rachapelle et al 2013.[27]<br>Assumption that the effect of no screening is as in historic studies that reported the rate at which STDR leads to blindness if left untreated.	Data collected in the study, data from the literature and expert opinion where data could not be collected.
Efficiency	Efficiency is defined as optimal use of the service. For this purpose, we will first estimate the time required to process one patient from the path-process analysis. This estimate will be used to determine the number of patients who can be seen on a day. We will then calculate (1-number of patients screened)/number of patients that can be screened). proportion can be used in a p-type control chart.[28]	Capture efficiencies in structures, resources and processes across the DR care pathway from staff interviews.<br><br>Path process mining of patients screened in FHCs analysis will be based on detailed time-event logs of approximately 70 patients from the 16 FHCs
Patient satisfaction	Patients willingness to be screened and their satisfaction with the information provided to them and the care they received	Patient interviews and telephone survey of patients referred to secondary care for treatment
Timeliness	Path process mapping at FHCs to understand delays in pathway<br>Reasons for non-attendance for those referred to secondary care for treatment of STDR	Path process mapping at FHCs to understand delays in DR care pathway<br><br>Patient survey and secondary care records

For peer review only - http://bmjopen.bmj.com/site/about/guidelines.xhtml

Equity	Age and gender-based prevalence of DR and STDR will be reported on consented patients from all FHCs. Reasons of non-referral of patients that require referrals will be recorded. Numbers denied screening despite willingness to be screened will also be reported.	Data from EHR and study database.

FHCs -family health centres; DR -diabetic retinopathy; STDR-sight threatening diabetic retinopathy; QALY- quality-adjusted life year, EHR-electronic health records

Table 3: Outcomes of the evaluation of the implementation strategy

Outcomes	Indicators	Assessment and data source
Acceptability	Willingness of each FHC to be involved in the DR care pathway.	**Assessment:** Readiness to implement the DR care pathway in the 16 FHCs and any reasons for delay.<br>*Data source:* Clinical performance from FHCs that will include monthly uptake of DR screening at each FHC and any reports on barriers causing delays in implementation provided by the project manager.
	Staff willingness to train	**Assessment:** Proportions of trained ASHAs, nurses and doctors per FHC, increase in doctors trained in laser surgery in secondary care, proportions willing to be included in train the trainer programme. The denominator will be total numbers of each personnel available to be trained on the date of implementation.<br>*Data sources:* Accreditation and certification records; Structured interviews of at least 5 nurses and 5 primary care doctors from 16 FHCs, 3-5 ophthalmologists from secondary and tertiary care, 2 data entry operators and 5 ASHAs and 1 health service administrator to understand challenges for acceptance.
	Patients acceptability of the pathway	**Assessment:** Screening attendances per FHC over 12 months.<br>*Data sources:* Clinical performance data and a telephone questionnaire survey for screened and referred patients to evaluate reasons for attendance and non-attendance.

For peer review only

Adoption	Uptake of DR care pathway at FHCs, secondary and tertiary care<br><br>Uptake of training by staff<br><br>Uptake of screening programme by patients<br><br>Adoption of DR care pathway by the Government of Kerala for state-wide implementation.	**Assessment:** Proportion of staff at primary, secondary and tertiary care willing to integrate DR care pathway in their workload.<br><br>*Data source:* FHC data and staff interviews<br><br>**Assessment:** Demand training sessions; Attendance rates at training sessions<br><br>*Data source:* number of training sessions and attendance registers at training sessions.<br><br>**Assessment:** Increase in proportions of patient screened over 12 months.<br><br>*Data source:* EHR and study database.<br><br>**Assessment:** A policy paper by the Government of Kerala for state-wide adoption of the DR care pathway as part of diabetes care for all patients.<br><br>*Data source:* A detailed scale-up plan will be developed for each district based on the clinical and cost-effectiveness data from this study to inform policy.
Appropriateness	Appropriateness of FHCs for DR screening<br><br>Appropriateness of the complex DR care pathway across primary and secondary care<br><br>Appropriateness of referrals to secondary care	**Assessment:** Qualitative data on barriers and facilitators of the delivery of screening and treatment in secondary care.<br><br>*Data sources:* interview of staff at FHCs and secondary care, patient telephone survey on pathway from FHCs to secondary care<br><br>**Assessment:** False positive referrals from data collection at secondary care.<br><br>*Data sources:* study database; data from patient telephone survey
Cost	Costs to the Government of Kerala and societal costs of the DR screening programme and subsequent treatment	**Assessment:** Costs of the DR screening and treatment costs in total and per person, including the costs of staff training and cameras (suitably annuitized), staff costs and travel costs of patients.<br><br>*Data sources:* Data collected in the study baseline and data from other services in India and from the literature.

For peer review only

Feasibility	Screening rate per FHC<br>Barriers to monthly screening uptake	**Assessment:** Monthly increase in numbers of FHCs added to the programme<br><br>*Data source:* Interview and monthly report from project manager on facilitators and barriers on each part of the pathway
Fidelity	Degree of the DR care pathway implementation	**Assessment:** Fidelity evaluation in 16 FHC clinics using a path-process analysis of the screening to identify the common screening pathway across FHCs.<br><br>*Data source:* The path-process analysis will be based on detailed time-event logs of 70 patients from the 16 FHCs; a telephone follow-up of referred patients to evaluate uptake of referrals.
	Validation of the DR grading within the telemedicine pathway	**Assessment:** Validation of the DR grading will be assessed by the agreement of the DR grade reported by the optometrists at the reading centre in RIO and those reported by the ophthalmologists in the secondary care centres.<br><br>*Data sources:* DR grading of referred patients obtained from the reading centre versus secondary care.
	Delivery of training and the impact on the DR care pathway	**Assessment:** Increase in numbers of trained staff per month.<br><br>*Data sources:* Staff interviews, structured observations and review of facility records. The proportions of doctors trained on laser delivery; data quality on the database; Increase in NCD registration following start of programme due to training of ASHA workers on diabetes and DR; Increase in numbers of patients treated for STDR following training.
Penetration	Increase patient awareness of diabetes and DR and uptake of the DR screening pathway in patients with diabetes	**Assessment**: Increase uptake of DR screening by patients registered in NCD registers.<br><br>*Data sources:* from NCD register and EHR
Sustainability	Policy makers to prepare policy on up-scale and sustainability plan for DR pathway	**Assessment:** Availability of a policy paper from Government of Kerala on state-wide scale up of the DR care pathway. This will be considered a success of the programme.<br><br>*Data sources:* Scale up plan for other districts including plan to purchase retinal cameras and

For peer review only - http://bmjopen.bmj.com/site/about/guidelines.xhtml

		acceptance of guidelines and training material for state-wide roll out.
	Integration of DR pathway in weekly activities in FHCs	**Assessment:** Integration of DR pathway in EHR<br><br>*Data source:* EHR records of DR screening as part of NCD care pathway.

FHC -family health centres; DR-diabetic retinopathy; NCD-non-communicable diseases; ASHA -accredited social health activists, EHR- electronic health records

Data collection

Quantitative data

Effectiveness outcomes will originate from the data sources shown in table 2. Data collected by nurses or data operators from the EHR include age, gender, duration of diabetes, use of insulin, parental history of diabetes, other complications of diabetes including diabetic kidney disease, cardiovascular complications, and diabetic foot, random blood sugar results, urine dipstick test for albuminuria and blood pressure record. Other study-specific data collected by nurses or data operators on the day of screening include education status, occupation and income categories, and previous history of DR, cataract surgery or any other ocular history. In addition, they will measure body mass index, waist circumference and complete a lifestyle questionnaire on smoking, diet, physical activity and EQ-5D-5L vision bolt-on, an instrument to measure health-related quality of life and vison[29] (see supplementary file 1). .

In the reading centre, the data collection will include the grade of retinopathy in both eyes, presence of cataract and gradability of the retinal images. Data collected on referred patients will include numbers with ungradable images due to cataract, treatment options offered for DR and review appointment.

Data Monitoring

Data will be coded before entry into the study database by the clinical teams. Only anonymised extracted data from EHR by the nurses or data entry operators will be used for analysis. Data quality will be monitored by the study project manager. Anonymised data will be checked for range checks and data quality at University of East London by the study statistics team. The ORNATE India International Advisory Board will have an overview of the conduct of the project

For peer review only

and the Executive Group consisting of the UK-India collaborators will monitor the conduct of the study.

Qualitative data collection and analysis

Data sources used for evaluation of the implementation strategy are shown in table 3. These will include data from a structured interview of a maximum variable sample that will reflect the context (primary, secondary and tertiary care), and the functions and skill levels of the staff (e.g. nurses, doctors etc.). Based on pragmatic considerations, at least 5 nurses and 5 primary care doctors from 16 FHCs, 3-5 ophthalmologists from secondary and tertiary care, 2 data entry operators and 5 ASHAs and 1 health service administrator should be included to get the maximum variability. Verbal consent will be obtained from these health professionals. The interviews will be conducted by an independent member from the GCRF/UKRI-funded team, who is not involved in this study, in the local language within the premises of the healthcare provider. The focus group will consist of groups of patients and staff within one FHC. In addition, to the voice-recording of the interviews and focus group, interviewers will write field notes to describe the interview situation. Field notes, the interviews and focus group content will provide the basis for the data analysis, which will be based on a descriptive phenomenological approach without data or opinion interpretation and will include transcription, condensation, coding and categorisation using qualitative analysis tools. A survey of all referred patients will be done using a structured questionnaire to evaluate their satisfaction and their perception of the barriers and facilitators. All qualitative data will be coded using NVivo and analysed using thematic analysis following the strategy.[30]

Sample size

We have chosen the proportion of patients in the NCD register with diabetes screened for DR as our primary outcome variable.

Justification of the choice of primary outcome: This variable captures the effectiveness of our intervention as well as the fidelity of implementation. Other facts that informed our choice of the primary outcome is that we expect a short implementation time of 7 to 9 months during which other outcomes such as number of patients treated may not be a feasible option. Therefore, we have estimated the sample size based on this primary outcome.

Our calculations are complicated by the expected increase in number of people with diabetes in the NCD register as public awareness of diabetes and DR increases so the denominator of numbers screened will be the number of people registered in the NCD in each FHC at the start of the cross-sectional study. There is no data on the baseline proportion of patients that attend

For peer review only - http://bmjopen.bmj.com/site/about/guidelines.xhtml

the tertiary centre for screening. The prevalence of diabetes is between 10-16% in Thiruvananthapuram of which 8% are estimated to have DR and 3% to have STDR but about 20% may have to be referred. Assuming a finite population of 40,000 patients with diabetes, a simple random sample of 377 patients will be needed. However, we expect large design effects due to clustering patients within FHCs. There is not enough data to calculate the value of this design effect and therefore we assume it to be 3 to give a final sample size of 1,131, which is equivalent to assuming a within-FHC intracluster correlation coefficient of approximately 0.03 (n=16 FHCs) with negligible residual clustering by the intermediate cluster level of ASHAs, where mean cluster size is smaller.

As described by Becker et al in mammography screening,[31] we expect sources of bias in the implementation programme that will likely influence the sample size calculation. Unscreened DR cases that already exist in the community may contribute to an over-estimation of the effect of screening. We expect lead-time bias because of our short implementation period and so some DR in the community may be missed. A comparison of numbers with DR in FHC with longer implementation period with those of shorter period may adjust for this bias. We intend to provide a descriptive analysis as well as a temporal comparison using time series analysis.

Statistical Analyses

We will create a complete case dataset of the cross-sectional survey for use in the analyses of effectiveness and assess the potential impact of missing data using sensitivity analysis incorporating a range of optimistic and pessimistic scenarios for the impact of missing data. We will model two outcomes that we hypothesised would be influenced by the intervention: (1) uptake of DR screening (primary outcome) and (2) numbers of patients referred for STDR as a result of screening. We will use bivariate logistic regression models to assess crude associations between sociodemographic factors such as age, gender, education, income, and living arrangements, and other clinical data with DR and STDR and use fractional polynomials to account for the form of continuous covariates to predict uptake of DR screening. Factors associated with a $P<0.25$ will be candidates for inclusion in the multivariable logistic regression models. We will test interactions amongst identified main effects to capture improvement in model prediction assessed by reduced residual variance-based statistics. To account for the staggered entry of the FHCs into the study, we will add a variable indicating the month of entry of each FHC into the programme (e.g. 1) if the FHC join in the first month of the programme and 2) if in the second month and so on). A non-significant coefficient for this variable will suggest the staggered approach had no effect and a significant coefficient will allow the effect to be

For peer review only

For peer review only - http://bmjopen.bmj.com/site/about/guidelines.xhtml

quantified for each FHC. In our final adjusted model, we will consider associations with a P<.05 as statistically significant. Adequacy of model discrimination and calibration will be assessed using Receiver Operating Characteristic (ROC) curves. The validation of the telemedicine will be reported as the agreement (kappa statistics) between screen positive patients graded by the graders at the reading centre versus the DR grade as recorded by the ophthalmologists in secondary care centres.

Patient and public involvement (PPI)

A patient and public group will be involved in plans to disseminate the study results and provide their input on the scale-up of this DR care pathway, should the implementation in Thiruvananthapuram be deemed successful by the Government of Kerala.

Ethics and dissemination

The study was approved by Indian Medical Research Council (2018-0551).The study complies with the guidelines of the Declaration of Helsinki. Results of this research are expected to be disseminated through stakeholder reports and via scientific forums, specifically peer-reviewed publications and conference presentations. All participants will give written informed consent prior to entry to the study by the FHC nurses and will be made aware that participation is strictly voluntary-see consent form in supplementary file 2.

Discussion

At the conclusion of this study, we hope to assess the effectiveness and implementation challenges of a complex DR care pathway integrating care at primary, secondary and tertiary care, covering a proportion of the diabetic population in Thiruvananthapuram. This pilot data will be useful for Kerala and perhaps India to plan to achieve the SDGs. The study will set the scene for a policy for State-wide Screening and Treatment Pathway for Diabetes Complications with clinical impact on the patient, impact on the community, impact on staff workload and economic impact on family and public finances and integrating it to primary care practice. Early identification of STDR and other complications due to this holistic approach and timely treatment, will have positive impact on rates of blindness, chronic kidney disease, cardiovascular complications and thereby improve health, reduce multi-morbidity and mortality. A DR pathway that straddles primary, secondary and tertiary levels of care, leveraging technology will have advantages of cost effectiveness and ease of implication in LMICs as

For peer review only - http://bmjopen.bmj.com/site/about/guidelines.xhtml

against the current practice of detection and management of self-reported cases in tertiary centres.

This study outlines how the effectiveness of a DR care pathway and its implementation will be evaluated. [21, 22] This study is timely given the increasing numbers of people with diabetes and pressure on finances available for healthcare, necessitating task shifting to enable better coverage of the population. Although it is a DR care pathway, we will collect data on other complications of diabetes to address the wider needs of this group of patients. The study will also examine the organisation functions such as structure, resources and processes that would contribute to better outcomes. We will also examine whether this additional task is feasible given the current skill levels and workload of FHCs and secondary hospitals. The study will reveal whether current health seeking behaviour of patients will support screening, especially when an invasive procedure is requested when there is no obvious impact on quality of life of the patient. By addressing a key gap in knowledge due to lack of research in this area, we will be able to decipher the barriers and facilitators that influence the successful implementation of such a programme in the public system in India. The results of this study may inform the adoption of this pathway in other areas in India and globally. However, the complexity and number of implementation strategies and lack of validated implementation outcomes may limit generalisability of the results and implementation of this pathway elsewhere.

There are limitations, however, to the study design. We do not have baseline data on DR screening in the study location as these screening programmes are non-existent. For this reason, we are examining the effectiveness of the pathway in terms of presenting visual acuity for referable cases as 'proximal' outcomes. In addition, when there is no concurrent control group, causal inferences are difficult to make and may lead to both measured and unmeasured biases. When examining implementation, logistical issues, variations in health care facilities, socio-economic variation, and quality of health care personnel may likely affect our study. However, the study design avoids the ethical challenges of having a control group with no DR screening. Therefore, the study design is by necessity non-randomised and observational and will rely on newly trained staff members to collect data, which is likely to differ in completeness between the 16 FHCs. We expect an increase in referral rates after implementation of the intervention due to better public awareness, increasing knowledge of ASHAs and improved case ascertainment, at least in some FHCs. We will also examine the impact of cataract in the community and this has not been studied before in any other DR pathway. We have tried to minimise these risks by the use of robust statistical techniques and the use of various data

For peer review only

For peer review only - http://bmjopen.bmj.com/site/about/guidelines.xhtml

422 sources to elicit a greater understanding of how the programme will lead to better health
423 outcomes that will include identification of cataract.

424 This study has many strengths: Our study has quantitative data backed by qualitative data to
425 strengthen our findings and enable generalisation of our findings. Secondly, we will use robust
426 statistical methods to reduce bias including selection bias and other confounders. Finally, the
427 access to and collaboration with the UK is a key strength of the study, as it facilitates the co-
428 development of the interventions from the outset. Active involvement of policy makers engaged
429 in transformation of primary care to screen for and address complications of NCDs who value
430 research to generate evidence for policy making and who are prepared to learn from, and adapt
431 the DR based on, implementation experience is a unique feature of this study. We expect the
432 findings of this evaluation to be of interest to a wide audience given the growing need to
433 demonstrate effectiveness of complex interventions in low resource settings. Findings on the
434 mechanisms and contexts that optimise the implementation of this complex multi-faceted
435 intervention using the ERIC taxonomy will be useful to those developing and implementing
436 these programmes in other health systems. The health economic model may highlight the
437 health expenditure required at individual, family and Kerala State level for forecasting and
438 planning health budgets.

439 Perhaps the most important aspect of the chosen evaluation is that it is built within a
440 simultaneous developing public health strategy on population-based screening of diabetes and
441 hypertension and a recently introduced EHR called eHealth. Conducting research in such an
442 environment is a good example of health policy and systems research.

443 Despite the limitations, this study holds promise for providing high-quality data and detailed
444 implementation information on a complex intervention in a resources-limited setting. We hope to
445 contribute to the literature on the implementation and effectiveness of DR screening and
446 treatment in the public health sector in LMICs.

447 **Author Contributorship**

448

449 Sobha Sivaprasad (S.S), Gopalakrishnan Netuveli (G.N), Raphael Wittenberg (R.W), Rajan
450 Khobragade (R.K), Rajeev Sadanandan (R.S), Bipin Gopal (B.G), Lakshmi Premnazir (L.P),
451 Dolores Conroy (D.C), Jyotsna Srinath (J.S), Radha Ramakrishnan (R.R), Simon George (S.G),
452 Vasudeva Iyer Sahasranamam (V.S)

For peer review only - http://bmjopen.bmj.com/site/about/guidelines.xhtml

Conceptualisation, S.S, G.N, R.W, R.S, B.G, S.G, V.S; methodology, S.S, G.N, R.R, R.S, B.G, V.S, S.G, D.C, R.R, R.K; acquisition of data, S.S, G.N, R.W, D.C, R.R, J.S, L.P, B.G; writing—original draft preparation, S.S, D.C, G.N, R.W, L.P, R.R; writing—review and editing, S.S, G.N, R.R, R.S, B.G, V.S, S.G, D.C, R.R, R.K, J.S, L.P; funding acquisition, S.S, G.N, R.W, R.S, on behalf of the Collaborators.

Role of study sponsor and funders, if any, in study design; collection, management, analysis, and interpretation of data; writing of the report; and the decision to submit the report for publication, including whether they will have ultimate authority over any of these activities; None

Role of sponsor: The Director of Regional Institute of Ophthalmology contributed to study design; management, writing of the report; and the decision to submit the report for publication, but have no authority over any of these activities.

Authorship eligibility guidelines will be based on ICJME and there is no intention to use professional writers.

Acknowledgements:
Nayanamritham Project Collaborators: Dr Rajeevan Palpoo and Dr Chitra Raghavan will manage the referred patients in Regional Institute of Ophthalmology; Alwin Aniyankunju and Anju V.Molly will be responsible for grading the retinal images.
Steering Committee: Members of the International Advisory Board from Kerala

Funding statement: This work is part of the ORNATE India project funded by Global Challenges Research Fund and UK Research and Innovation through the Medical Research Council grant number MR/P027881/1

Data Sharing Agreement: The technical appendix, statistical code and dataset will be made available on request after obtaining permission from the Government of Kerala.

Competing interests statement: None

Sponsor: Dr V Sahasranamam, Director, Regional Institute of Ophthalmology, Thiruvananthapuram, Kerala

References

1. International Diabetes Federation. *IDF Diabetes Atlas*. 9th edition 2019 https://www.diabetesatlas.org/en/

For peer review only - http://bmjopen.bmj.com/site/about/guidelines.xhtml

2. Yau JW, Rogers SL, Kawasaki R, et al. Meta-Analysis for Eye Disease (META-EYE) Study Group. Global prevalence and risk factors of diabetic retinopathy. *Diabetes Care* 2012; 35:556–64.

3. IDF Diabetes Atlas: Global estimates of diabetes prevalence for 2017 and projections for 2045. Cho NH, Shaw JE, Karuranga S, Huang Y, da Rocha Fernandes JD, Ohlrogge AW, Malanda B Diabetes Res Clin Pract. 2018; 138():271-281.

4. Scanlon PH. The English National Screening Programme for diabetic retinopathy 2003-2016. *Acta Diabetol* 2017; 54(6):515-25

5. Cavan D, Makaroff L, da Rocha Fernandes J, et al. The Diabetic Retinopathy Barometer Study: Global perspectives on access to and experiences of diabetic retinopathy screening and treatment. *Diabetes Res Clin Pract* 2017; 129:16-24.

6. NITI Ayog (2020), SDG India Index, Baseline Report, 2018, New Delhi [Last accessed on 2021 Jan 17]. Available from: www.niti.gove.in

7. Menon VU, Kumar KV, Gilchrist A, et al. Prevalence of known and undetected diabetes and associated risk factors in central Kerala--ADEPS. *Diabetes Res Clin Pract* 2006; 74:289–94.

8. Sustainable Development Goals. United Nations. [Last accessed on 2020 May12]. Available from: http://www.un.org/sustainabledevelopment/sustainable-developmentgoals/

9. The WHO STEPwise approach to Surveillance of noncommunicable diseases (STEPS). Noncommunicable Diseases and Mental Health. World Health Organization. 20 Avenue Appia, 1211 Geneva 27, Switzerland.

10. Scanlon PH, Aldington SJ, Leal J, et al. Development of a cost-effectiveness model for optimisation of the screening interval in diabetic retinopathy screening. *Health Technol Assess* 2015;19(74):1-116.

11. Leese GP, Morris AD, Olson J. A national retinal screening programme for diabetes in Scotland. *Diabet Med* 2003;20(12):962-4.

12. Lund SH, Aspelund T, Kirby P et al. Individualised risk assessment for diabetic retinopathy and optimisation of screening intervals: a scientific approach to reducing healthcare costs. *Br J Ophthalmol*. 2016;100(5):683-7.

13. Manne-Goehler J, Geldsetzer P, Agoudavi K et al. Health system performance for people with diabetes in 28 low- and middle-income countries: A cross-sectional study of nationally representative surveys. *PLoS Med*. 2019;16(3):e1002751.

14. Ting DS, Cheung GC, Wong TY. Diabetic retinopathy: global prevalence, major risk factors, screening practices and public health challenges: a review. *Clin Exp Ophthalmol.* 201;44(4):260-77.

15. Hazin R, Barazi MK, Summerfield M. Challenges to establishing nationwide diabetic retinopathy screening programs. *Curr Opin Ophthalmol*. 2011;22(3):174-9.

16. Burgess PI, Msukwa G, Beare NA. Diabetic retinopathy in sub-Saharan Africa: meeting the challenges of an emerging epidemic. *BMC Med*. 2013;11:157.

17. Ramke J, Petkovic J, Welch V et al. Interventions to improve access to cataract surgical

For peer review only

For peer review only - http://bmjopen.bmj.com/site/about/guidelines.xhtml

services and their impact on equity in low- and middle-income countries. *Cochrane Database Syst Rev*. 2017;11(11):CD011307.

18. Rani PK, Bhattarai Y, Sheeladevi S et al. Analysis of yield of retinal imaging in a rural diabetes eye care model. *Indian J Ophthalmol*. 2018 Feb;66(2):233-237.

19. Scanlon PH, Foy C, Malhotra R, Aldington SJ. The influence of age, duration of diabetes, cataract, and pupil size on image quality in digital photographic retinal screening. *Diabetes Care*. 2005;28(10):2448-53.

20. Powell BJ, Waltz TJ, Chinman MJ et al. A refined compilation of implementation strategies: results from the Expert Recommendations for Implementing Change (ERIC) project. *Implement Sci*. 2015;10:21.

21. Powell BJ, McMillen JC, Proctor EK, et al. A compilation of strategies for implementing clinical innovations in health and mental health. *Med Care Res Rev*. 2012;69(2):123-157.

22. Smith JD, Li DH, Rafferty MR. The Implementation Research Logic Model: a method for planning, executing, reporting, and synthesizing implementation projects. *Implement Sci*. 2020 Sep 25;15(1):84.

23. Landes SJ, McBain SA, Curran GM. Reprint of: An introduction to effectiveness-implementation hybrid designs. *Psychiatry Res*. 2020 Jan;283:112630.

24. Rajalakshmi R, Subashini R, Anjana RM, et al. Automated diabetic retinopathy detection in smartphone-based fundus photography using artificial intelligence. *Eye* (Lond) 2018;32(6):1138-1144

25. Piyasena MMPN, Murthy GVS, Yip JLY et al. Systematic review and meta-analysis of diagnostic accuracy of detection of any level of diabetic retinopathy using digital retinal imaging. *Syst Rev*. 2018 Nov 7;7(1):182. doi: 10.1186/s13643-018-0846-y. Erratum in: Syst Rev. 2019 Apr 30;8(1):106.

26. Boucher MC, Nguyen MTD, Qian J. Assessment of Training Outcomes of Nurse Readers for Diabetic Retinopathy Telescreening: Validation Study. *JMIR Diabetes*. 2020;5(2):e17309.

27. Rachapelle S, Legood R, Alavi Y, Lindfield R, Sharma T, Kuper H, Polack S. The cost-utility of telemedicine to screen for diabetic retinopathy in India. *Ophthalmology*. 2013 Mar;120(3):566-573.

28. NIST/SEMATECH e-Handbook of Statistical Methods. Proportion Control Chart https://www.itl.nist.gov/div898/handbook/pmc/section3/pmc332.htm, Accessed 5/1/2021

29. Longworth L, Yang Y, Young T et al. Use of generic and condition-specific measures of health-related quality of life in NICE decision-making: a systematic review, statistical modelling and survey. *Health Technol Assess*. 2014 Feb;18(9):1-224.

30. Braun V, Clarke V. What can "thematic analysis" offer health and wellbeing researchers? *Int J Qual Stud Health Well-being*. 2014 Oct 16;9:26152.

31. Becker N, Hakama M, Nyström L. Evaluation of effectiveness of quality-assured

For peer review only - http://bmjopen.bmj.com/site/about/guidelines.xhtml

mammography screening in Germany: sample size considerations and design options. *Eur J Cancer Prev* 2007;16(3):225-31.

Legend: Figure 1: Proposed Diabetic Retinopathy Care Pathway

Figure 2: The Implementation Research Logic Model

Supplementary files

1. EQ5D-5L-Vision Bolt on
2. Consent form for the Nayanamritham study

For peer review only

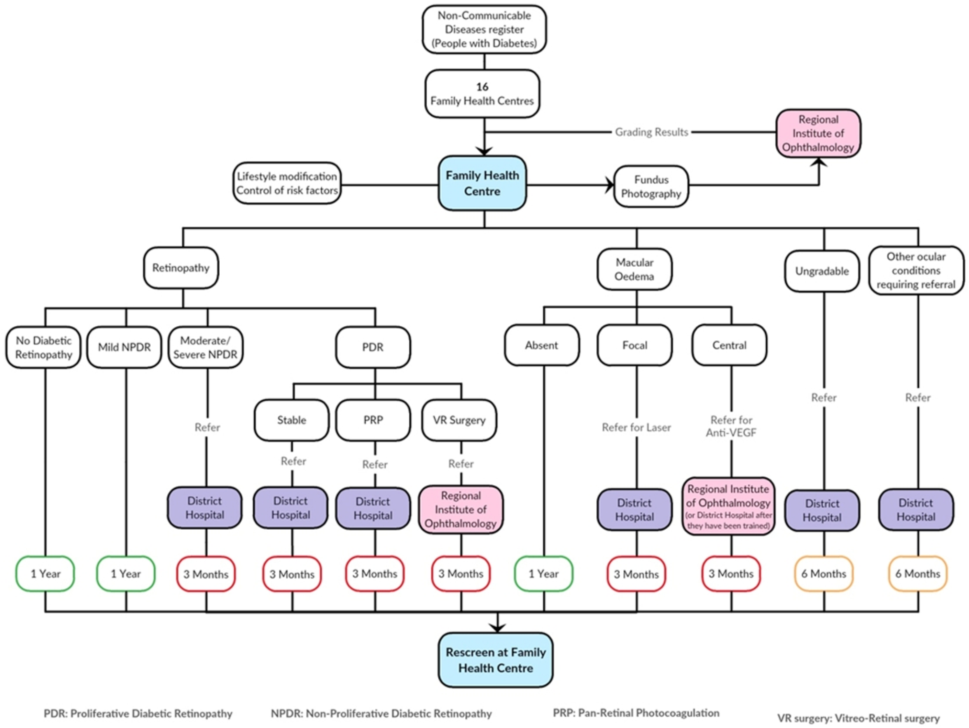

Figure !

186x138mm (300 x 300 DPI)

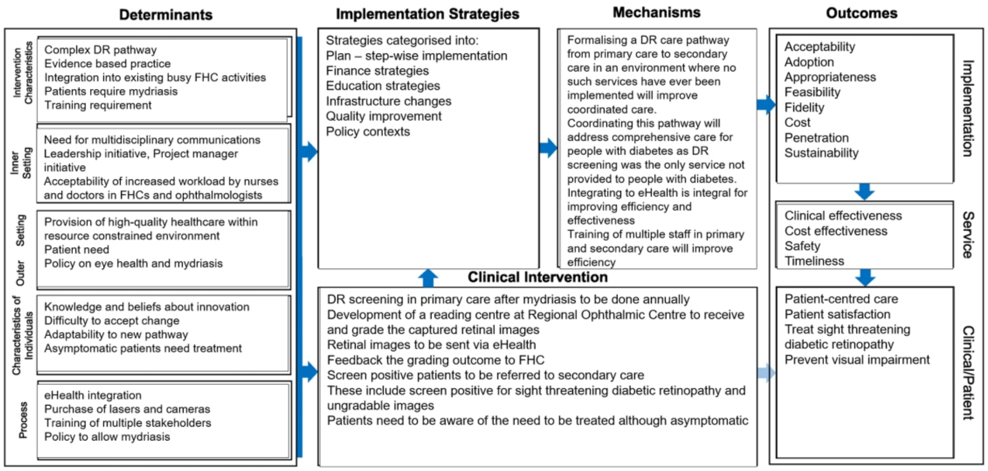

Figure 2 The Implementation Research Logic Model

188x90mm (300 x 300 DPI)

Supplementary file 1

EQ5D-5L-Vision Bolt on

By placing a tick in one box in each group below, please indicate which statements best describe your own health state today

**1.**	**Mobility**	**1**	**I have no problems in walking about**
		**2**	**I have slight problems in walking about**
		**3**	**I have moderate problems in walking about**
		**4**	**I have severe problems in walking about**
		**5**	**I am unable to walk about**
		777	*Not available or not applicable*
		888	*Not done*
		999	*Unknown*

**2.**	**Self-Care**	**1**	**I have no problems washing or dressing myself**
		**2**	**I have mild problems washing or dressing myself**
		**3**	**I have moderate problems washing or dressing myself**
		**4**	**I have severe problems washing or dressing myself**
		**5**	**I am unable to wash or dress myself**
		777	*Not available or not applicable*
		888	*Not done*
		999	*Unknown*

**3.**	**Usual Activities** (*e.g. work, study, housework, family or leisure activities)*	**1**	**I have no problems with performing my usual activities**
		**2**	**I have mild problems with performing my usual activities**
		**3**	**I have moderate problems with performing my usual activities**
		**4**	**I have severe problems with performing my usual activities**
		**5**	**I am unable to perform my usual activities**
		777	*Not available or not applicable*
		888	*Not done*
		999	*Unknown*

**4.**	**Pain/ Discomfort**	**1**	**I have no pain or discomfort**
		**2**	**I have mild pain or discomfort**
		**3**	**I have moderate pain or discomfort**
		**4**	**I have severe pain or discomfort**
		**5**	**I have extreme pain or discomfort**
		777	*Not available or not applicable*
		888	*Not done*
		999	*Unknown*

**5.**	**Anxiety/ Depression**	**1**	**I am not anxious or depressed**
		**2**	**I am mildly anxious or depressed**
		**3**	**I am moderately anxious or depressed**
		**4**	**I am severely anxious or depressed**
		**5**	**I am extremely anxious or depressed**
		777	*Not available or not applicable*
		888	*Not done*
		999	*Unknown*

**6.**	**Vision (using glasses or contact lenses if needed)**	**1**	**I have no problems seeing**
		**2**	**I have slight problems seeing**
		**3**	**I have some problems seeing**
		**4**	**I have severe problems seeing**
		**5**	**I am unable to see**
		777	*Not available or not applicable*
		888	*Not done*
		999	*Unknown*

Supplementary file 2

THE NAYANAMRITHAM STUDY CONSENT FORM

I understand that diabetic retinopathy is a condition that effects many people with diabetes. I agree to participate in the Nayanamritham study – A study on the challenges in the diagnosis and management of diabetic retinopathy in the public health sector of Kerala. As a part of this study, a photo of my eye will be captured with the help of a camera and diagnosis of diabetic retinopathy will be made after analysing the images. If I am diagnosed with this condition, the treatment for the same would be carried out in primary health centres, taluk and district hospitals and government eye hospital -Trivandrum

I understand and agree that during my participation in the study, my anonymized data pertaining to the diabetic retinopathy will be collected and stored on paper and / or electronically. These may also be accessed and reviewed by competent authorities from India and abroad who are obliged to keep my identity confidential and I also understand that this can lead to the development of newer strategies for the management of diabetic retinopathy for the public

This study has been explained to me in detail, including the goals of the research, the procedures involved, the potential risks and benefits, and all of my questions have been answered to my satisfaction. I understand that my decision to take part in the study is entirely voluntary. I am free to change my mind and withdraw from the study at any time. Such withdrawal will not affect the standard medical care I will receive. I also understand that participating in this study will not result in any adverse financial implications. By signing and dating this consent form, I have not waived any of the legal rights that I would have if I were not a participant in a research study. I agree to take part in the above study.

For peer review only

STANDARD PROTOCOL ITEMS: RECOMMENDATIONS FOR INTERVENTIONAL TRIALS

SPIRIT 2013 Checklist: Recommended items to address in a clinical trial protocol and related documents*

Section/item	Item No	Description	Addressed on page number
**Administrative information**			
Title	1	Descriptive title identifying the study design, population, interventions, and, if applicable, trial acronym	_ 1____________
Trial registration	2a	Trial identifier and registry name. If not yet registered, name of intended registry	__1___________
	2b	All items from the World Health Organization Trial Registration Data Set	__NA__________
Protocol version	3	Date and version identifier	_1____________
Funding	4	Sources and types of financial, material, and other support	__25_____
Roles and responsibilities	5a	Names, affiliations, and roles of protocol contributors	__24___________
	5b	Name and contact information for the trial sponsor	__25___________
	5c	Role of study sponsor and funders, if any, in study design; collection, management, analysis, and interpretation of data; writing of the report; and the decision to submit the report for publication, including whether they will have ultimate authority over any of these activities	__25___________
	5d	Composition, roles, and responsibilities of the coordinating centre, steering committee, endpoint adjudication committee, data management team, and other individuals or groups overseeing the trial, if applicable (see Item 21a for data monitoring committee)	__19_________

For peer review only

**Introduction**			
Background and rationale	6a	Description of research question and justification for undertaking the trial, including summary of relevant studies (published and unpublished) examining benefits and harms for each intervention	__4_and 5
	6b	Explanation for choice of comparators	__6____________
Objectives	7	Specific objectives or hypotheses	__6___________
Trial design	8	Description of trial design including type of trial (eg, parallel group, crossover, factorial, single group), allocation ratio, and framework (eg, superiority, equivalence, noninferiority, exploratory)	___6___________
**Methods: Participants, interventions, and outcomes**			
Study setting	9	Description of study settings (eg, community clinic, academic hospital) and list of countries where data will be collected. Reference to where list of study sites can be obtained	___7___________
Eligibility criteria	10	Inclusion and exclusion criteria for participants. If applicable, eligibility criteria for study centres and individuals who will perform the interventions (eg, surgeons, psychotherapists)	___7 _________
Interventions	11a	Interventions for each group with sufficient detail to allow replication, including how and when they will be administered	___7___________
	11b	Criteria for discontinuing or modifying allocated interventions for a given trial participant (eg, drug dose change in response to harms, participant request, or improving/worsening disease)	____7-8_______
	11c	Strategies to improve adherence to intervention protocols, and any procedures for monitoring adherence (eg, drug tablet return, laboratory tests)	_____18________
	11d	Relevant concomitant care and interventions that are permitted or prohibited during the trial	_____NA_______ _
Outcomes	12	Primary, secondary, and other outcomes, including the specific measurement variable (eg, systolic blood pressure), analysis metric (eg, change from baseline, final value, time to event), method of aggregation (eg, median, proportion), and time point for each outcome. Explanation of the clinical relevance of chosen efficacy and harm outcomes is strongly recommended	table 2 and 3

For peer review only - http://bmjopen.bmj.com/site/about/guidelines.xhtml

Participant timeline	13	Time schedule of enrolment, interventions (including any run-ins and washouts), assessments, and visits for participants. A schematic diagram is highly recommended (see Figure)	Figure 1
Sample size	14	Estimated number of participants needed to achieve study objectives and how it was determined, including clinical and statistical assumptions supporting any sample size calculations	20
Recruitment	15	Strategies for achieving adequate participant enrolment to reach target sample size	table 1

Methods: Assignment of interventions (for controlled trials)

Allocation:

Sequence generation	16a	Method of generating the allocation sequence (eg, computer-generated random numbers), and list of any factors for stratification. To reduce predictability of a random sequence, details of any planned restriction (eg, blocking) should be provided in a separate document that is unavailable to those who enrol participants or assign interventions	_NA_________
Allocation concealment mechanism	16b	Mechanism of implementing the allocation sequence (eg, central telephone; sequentially numbered, opaque, sealed envelopes), describing any steps to conceal the sequence until interventions are assigned	NA________
Implementation	16c	Who will generate the allocation sequence, who will enrol participants, and who will assign participants to interventions	NA_______
Blinding (masking)	17a	Who will be blinded after assignment to interventions (eg, trial participants, care providers, outcome assessors, data analysts), and how	NA_________
	17b	If blinded, circumstances under which unblinding is permissible, and procedure for revealing a participant's allocated intervention during the trial	NA________

Methods: Data collection, management, and analysis

Data collection methods	18a	Plans for assessment and collection of outcome, baseline, and other trial data, including any related processes to promote data quality (eg, duplicate measurements, training of assessors) and a description of study instruments (eg, questionnaires, laboratory tests) along with their reliability and validity, if known. Reference to where data collection forms can be found, if not in the protocol	_20-21____

	18b	Plans to promote participant retention and complete follow-up, including list of any outcome data to be collected for participants who discontinue or deviate from intervention protocols	____8-12______
Data management	19	Plans for data entry, coding, security, and storage, including any related processes to promote data quality (eg, double data entry; range checks for data values). Reference to where details of data management procedures can be found, if not in the protocol	____17-18__
Statistical methods	20a	Statistical methods for analysing primary and secondary outcomes. Reference to where other details of the statistical analysis plan can be found, if not in the protocol	____ 21_______
	20b	Methods for any additional analyses (eg, subgroup and adjusted analyses)	______21______
	20c	Definition of analysis population relating to protocol non-adherence (eg, as randomised analysis), and any statistical methods to handle missing data (eg, multiple imputation)	____NA________ _
**Methods: Monitoring**			
Data monitoring	21a	Composition of data monitoring committee (DMC); summary of its role and reporting structure; statement of whether it is independent from the sponsor and competing interests; and reference to where further details about its charter can be found, if not in the protocol. Alternatively, an explanation of why a DMC is not needed	____19________
	21b	Description of any interim analyses and stopping guidelines, including who will have access to these interim results and make the final decision to terminate the trial	____NA________ _
Harms	22	Plans for collecting, assessing, reporting, and managing solicited and spontaneously reported adverse events and other unintended effects of trial interventions or trial conduct	_____NA_______ _
Auditing	23	Frequency and procedures for auditing trial conduct, if any, and whether the process will be independent from investigators and the sponsor	____NA________ _
**Ethics and dissemination**			
Research ethics approval	24	Plans for seeking research ethics committee/institutional review board (REC/IRB) approval	______22_____

Protocol amendments	25	Plans for communicating important protocol modifications (eg, changes to eligibility criteria, outcomes, analyses) to relevant parties (eg, investigators, REC/IRBs, trial participants, trial registries, journals, regulators)	___1__________
Consent or assent	26a	Who will obtain informed consent or assent from potential trial participants or authorised surrogates, and how (see Item 32)	____22_________
	26b	Additional consent provisions for collection and use of participant data and biological specimens in ancillary studies, if applicable	____NA________
Confidentiality	27	How personal information about potential and enrolled participants will be collected, shared, and maintained in order to protect confidentiality before, during, and after the trial	_____19
Declaration of interests	28	Financial and other competing interests for principal investigators for the overall trial and each study site	___25__________
Access to data	29	Statement of who will have access to the final trial dataset, and disclosure of contractual agreements that limit such access for investigators	____25_________
Ancillary and post-trial care	30	Provisions, if any, for ancillary and post-trial care, and for compensation to those who suffer harm from trial participation	____NA________ _
Dissemination policy	31a	Plans for investigators and sponsor to communicate trial results to participants, healthcare professionals, the public, and other relevant groups (eg, via publication, reporting in results databases, or other data sharing arrangements), including any publication restrictions	____22_________
	31b	Authorship eligibility guidelines and any intended use of professional writers	____25_________
	31c	Plans, if any, for granting public access to the full protocol, participant-level dataset, and statistical code	___25_________
**Appendices**			
Informed consent materials	32	Model consent form and other related documentation given to participants and authorised surrogates	supplementary document 2
Biological specimens	33	Plans for collection, laboratory evaluation, and storage of biological specimens for genetic or molecular analysis in the current trial and for future use in ancillary studies, if applicable	________NA____ _

For peer review only - http://bmjopen.bmj.com/site/about/guidelines.xhtml

*It is strongly recommended that this checklist be read in conjunction with the SPIRIT 2013 Explanation & Elaboration for important clarification on the items. Amendments to the protocol should be tracked and dated. The SPIRIT checklist is copyrighted by the SPIRIT Group under the Creative Commons "Attribution-NonCommercial-NoDerivs 3.0 Unported" license.

For peer review only

BMJ Open

Complex interventions to implement a diabetic retinopathy care pathway in the public health system in Kerala: the Nayanamritham study protocol.

Journal:	*BMJ Open*
Manuscript ID	bmjopen-2020-040577.R4
Article Type:	Protocol
Date Submitted by the Author:	15-Apr-2021
Complete List of Authors:	Sivaprasad, Sobha; UCL, NIHR Moorfields Biomedical Research Centre; Moorfields Eye Hospital NHS Foundation Trust, Medical Retina Department<br>Netuveli, Gopalakrishnan; University of East London - Duncan House Campus, Institute of Connected Communities<br>Wittenberg, Raphael; Oxford University, Nuffield Department of Primary Health Care Sciences<br>Khobragade, Rajan; Government of Kerala, Directorate of Health<br>Sadanandan, Rajeev; Government of Kerala, Directorate of Health<br>Gopal, Bipin; Government Medical College Thiruvananthapuram, Non-Communicable Diseases Department, Directorate of Health Services<br>Premnazir, Lakshmi; Government Medical College Thiruvananthapuram, Directorate of Health Services<br>Conroy, Dolores; UCL, Department of Vision Sciences, Institute of Ophthalmology<br>Srinath, Jyotsna; University of East London - Duncan House Campus, Department of connected communities<br>Ramakrishnan, Radha; UCL, Department of Vision Sciences, Institute of Ophthalmology<br>George, Simon; Government Medical College Thiruvananthapuram, Regional Institute of Ophthalmology<br>Sahasranamam, Vasudeva Iyer ; Government Medical College Thiruvananthapuram, Ophthalmology Department, Regional Institute of Ophthalmology
**Primary Subject Heading**:	Ophthalmology
Secondary Subject Heading:	Public health, Diabetes and endocrinology
Keywords:	Diabetic retinopathy < DIABETES & ENDOCRINOLOGY, General diabetes < DIABETES & ENDOCRINOLOGY, Protocols & guidelines < HEALTH SERVICES ADMINISTRATION & MANAGEMENT

I, the Submitting Author has the right to grant and does grant on behalf of all authors of the Work (as defined in the below author licence), an exclusive licence and/or a non-exclusive licence for contributions from authors who are: i) UK Crown employees; ii) where BMJ has agreed a CC-BY licence shall apply, and/or iii) in accordance with the terms applicable for US Federal Government officers or employees acting as part of their official duties; on a worldwide, perpetual, irrevocable, royalty-free basis to BMJ Publishing Group Ltd ("BMJ") its licensees and where the relevant Journal is co-owned by BMJ to the co-owners of the Journal, to publish the Work in this journal and any other BMJ products and to exploit all rights, as set out in our licence.

The Submitting Author accepts and understands that any supply made under these terms is made by BMJ to the Submitting Author unless you are acting as an employee on behalf of your employer or a postgraduate student of an affiliated institution which is paying any applicable article publishing charge ("APC") for Open Access articles. Where the Submitting Author wishes to make the Work available on an Open Access basis (and intends to pay the relevant APC), the terms of reuse of such Open Access shall be governed by a Creative Commons licence – details of these licences and which Creative Commons licence will apply to this Work are set out in our licence referred to above.

Other than as permitted in any relevant BMJ Author's Self Archiving Policies, I confirm this Work has not been accepted for publication elsewhere, is not being considered for publication elsewhere and does not duplicate material already published. I confirm all authors consent to publication of this Work and authorise the granting of this licence.

For peer review only

Complex interventions to implement a diabetic retinopathy care pathway in the public health system in Kerala: the Nayanamritham study protocol.

Sobha Sivaprasad[1, 2], Gopalakrishnan Netuveli[3], Raphael Wittenberg[4], Rajan Khobragade[5], Rajeev Sadanandan[5], Bipin Gopal[6], Lakshmi Premnazir[6], Dolores Conroy[2], Jyotsna Srinath[3], Radha Ramakrishnan[2], Simon George[7], Vasudeva Iyer Sahasranamam[7] for the Nayanamritham Project Collaborators.

1. Medical Retina Department, NIHR Biomedical Research Centre, Moorfields Eye Hospital NHS Foundation Trust and University College London, London, UK
2. Department of Vision Sciences, UCL Institute of Ophthalmology, London, UK
3. Institute of Connected Communities, University of East London, UK
4. Nuffield Department of Primary Health Care Sciences, University of Oxford, Oxford, UK
5. Directorate of Health, Government of Kerala, India
6. Non-Communicable Diseases Department, Directorate of Health Services, Kerala, India
7. Ophthalmology Department, Regional Institute of Ophthalmology, Thiruvananthapuram, Kerala, India

Word count: 3696

Figures: 2

Table: 3

References: 31

Abstract: 295

Corresponding author

Professor Sobha Sivaprasad
NIHR Moorfields Biomedical Research Centre
Moorfields Eye Hospital NHS Foundation Trust,
162 City Road,
London EC1V 2PD, UK
Tel: 020 7253 3411
Email: senswathi@aol.com

Keywords: diabetic retinopathy, implementation study, Kerala, laser photocoagulation, diabetes, screening, visual impairment, cost-effectiveness.

Trial Registration Number: ISRCTN28942696

Protocol Version: 1.0 dated 13-03-2019

For peer review only

Abstract

Introduction: Using a type 2 hybrid effectiveness-implementation design, we aim to pilot a diabetic retinopathy (DR) care pathway in the public health system in Kerala to understand how it can be scaled up to and sustained in the whole state.

Methods and analysis: Currently, there is no systematic DR screening programme in Kerala. Our intervention is a teleophthalmology pathway for people with diabetes in the non-communicable disease registers in 16 family health centres. The planned implementation strategy of the pathway will be developed based on the discrete Expert Recommendations for Implementing Change (ERIC) taxonomy. We will utilise both quantitative data from a cross-sectional study and qualitative data obtained from structured interviews, surveys and group discussions with stakeholders to report the effectiveness of the DR care pathway and evaluation of the implementation strategy. We will use logistic regression models to assess crude associations DR and STDR and fractional polynomials to account for the form of continuous covariates to predict uptake of DR screening. The primary effectiveness outcome is the proportion of patients in the non-communicable disease register with diabetes screened for DR over 12 months. Other outcomes include cost-effectiveness, safety, efficiency, patient satisfaction, timeliness and equity. The outcomes of evaluation of the implementation strategies include acceptability, feasibility, adoption, appropriateness, fidelity, penetration, costs and sustainability. Addition of more family health centres during the staggered initial phase of the programme will be considered as a sign of acceptability and feasibility. In the long term, the state-wide adoption of the DR care pathway will be considered as a successful outcome of the Nayanamritham study.

Ethics and Dissemination: The study was approved by Indian Medical Research Council (2018-0551) dated 13/03/2019. Study findings will be disseminated through scientific publications and the report will inform adoption of the DR care pathway by Kerala state in future.

For peer review only

ARTICLE SUMMARY

- This study will examine the clinical and cost effectiveness of a new diabetic retinopathy care pathway at the patient, clinician and service levels and evaluate the implementation strategy within a resource constrained environment.
- The study will use a mixed-methods type 2 hybrid effectiveness-implementation design.
- The specific actions in the implementation strategy are based on the Expert Recommendations for Implementing Change (ERIC) taxonomy
- The study outlines the economic evaluation of the costs of the diabetic retinopathy care pathway**.**
- The study is limited by the absence of a comparator due to lack of previous data on the prevalence of diabetic retinopathy in the public health system.

For peer review only

For peer review only - http://bmjopen.bmj.com/site/about/guidelines.xhtml

INTRODUCTION

Background

The triad of diabetes, blindness and poverty is an urgent problem that needs an effective response in the Development Assistance Committee-listed countries as these countries are home to 80% of people with diabetes. [1] Diabetic retinopathy (DR) is one of the most common complications of diabetes. [2] Sight-threatening diabetic retinopathy (STDR) is a common cause of blindness in working-age people and unless this condition is managed early, it has considerable impact on the quality of life and productivity of the person and their family, as well as substantial financial costs to health systems. [3] In its early stages, STDR may be asymptomatic. Therefore, DR screening programmes are essential to identify STDR to enable timely treatment. [4] In DR screening programmes in high income countries, people with diabetes are systematically screened using office-based retinal cameras and the retinal images are graded according to severity of DR by dedicated accredited screeners and graders. Patients with STDR are identified and referred to ophthalmologists for timely treatment with laser and/or intravitreal injections of anti-vascular endothelial growth factor. [4] In order to successfully reduce the risk of blindness due to DR in low-middle income countries (LMICs), without a well-developed primary care infrastructure, the pathway needs to begin with systematically screening for diabetes, educating the public and healthcare professionals on early detection[5] and timely treatment of STDR and the need to optimally treat the risk factors for DR such as hyperglycaemia, hypertension and hyperlipidemia. [2] Moreover, DR screening and treatment are challenging due to the required technology and technical expertise needed to grade retinal images and deliver costly treatment options, adding to the cost and complexity of the required interventions.

Diabetes in Kerala

Kerala is the most advanced state in India in terms of literacy, health, social uplift and demographic transition.[6] This has been accompanied by high prevalence of diabetes. A recent report from Kerala suggests that one in five of the Kerala adult population may have diabetes.[7] The Government of Kerala launched the Aardram Mission in 2017 to transform and gear up the State's public health care system to achieve the Sustainable Development Goals (SDGs) in phases with short term goals on building infrastructure and quality care services. The overarching objectives of the Aardram Mission included providing equitable, affordable and quality care to citizens from all socio-economic strata; strengthening the public care system by decentralising healthcare from the secondary and tertiary level to primary care-led services and

For peer review only - http://bmjopen.bmj.com/site/about/guidelines.xhtml

initiating preventive medicine to address the impact of non-communicable diseases, especially hypertension and diabetes. Primary care centres have been converted to family health centres (FHCs) with three doctors and four nurses in each family health centre assigned to provide individual care plans to the allocated population whose records are tracked through a recently established Electronic Health Records (EHR) called eHealth. The transformation of primary care with a focus on non-communicable diseases provided the backdrop to the implementation of a DR care pathway.

The rationale for a complex DR care pathway

The Government of Kerala is pressing forward to achieve universal health coverage and address the sustained development goals (SDG) goals on poverty (SDG 1), health access (SDG 3), education (SDG 4) and gender equality (SDG 5). [8, 9] Therefore, there is an urgent need to tackle the complications of diabetes. Systematic DR screening has been shown to be effective in reducing blindness. [3] However, in high income countries, DR screening is achieved by dedicated services due to the technicalities and expertise required in DR screening and evaluation. [10-12] Introducing an isolated DR care pathway will not be sufficient to address the challenge in LMICs as these countries need to initiate holistic screening service for diabetes and all its complications simultaneously and embed DR screening within the primary care.

Rationale for an Implementation Strategy

Each aspect of the DR screening and care pathway has to be adapted to local needs and resources, requiring a locally appropriate implementation strategy. For example, mydriasis (pupil dilatation) is compulsory in some screening programmes but not in others and this process requires local approval by stakeholders. [10-12] In Kerala, mydriasis needs to be approved by the health department. Other issues faced by low and middle income countries are that the number of undiagnosed diabetes cases is high, [13] the non-availability of a state-wide diabetes register with recall facilities, the lack of resources for standardised non-portable retinal cameras, limited capacity of staff in primary care to screen for DR, and that the number of people with diabetes is larger than the capacity of the services provided by the secondary care hospitals. [14-16] Research capacity and capability are in their infancy in LMICs and implementing change in a busy environment when demands on staff are great and resources are limited is challenging. [15, 16] In addition, as cataract is more prevalent in low and middle income countries, [17] the proportion of ungradable retinal images is higher compared to developed countries. [17-19]

For peer review only - http://bmjopen.bmj.com/site/about/guidelines.xhtml

Therefore, we need to evaluate the implementation strategy using both qualitative and quantitative methods. [20-22]

Aims and Objectives

The Nayanamritham study is facilitated by a UK-Government of Kerala partnership as a part of the ORNATE-India project funded by Global Challenges Research Fund (GCRF) and UK Research and Innovation (UKRI). We aim to introduce a DR care pathway that spans primary, secondary and tertiary care in the public health system in Kerala in a pilot study in Thiruvananthapuram. The proposed study will (1) examine the clinical and cost effectiveness of the DR care pathway at the patient, clinician and service levels and (2) evaluate the implementation strategy of the pathway.

METHODS

Design: We chose a mixed-methods type 2 hybrid effectiveness-implementation design to evaluate the effectiveness of the clinical interventions and the implementation strategies. [20-23] The DR care pathway was developed by the Kerala Health Secretary, non-communicable diseases lead, Health and Medical Education service providers, technical and EHR teams, local authorities and the GCRF/UKRI- funded co-applicants from the UK. The DR care pathway will be set-up in a staggered approach with 5 family health centres initiated in the first three months and the remaining 11 centres will be added based on acceptance of all stakeholders and adapting and training phase for 12 months from 15-03-2018. A further 12-15 months will be allocated to recruit consenting patients to a cross-sectional study to gather quantitative data for the effectiveness outcomes. A minimal dataset from this study will also be entered into EHR to enable future screening for the consented patients. Qualitative data collection from interviews of staff, patients and focus groups at baseline and end of study and all field notes gathered during the study will be utilised to inform evaluation of the implementation strategy. [20-22]

Target Population

People with diabetes registered in the non-communicable diseases register at 16 family health centres in Thiruvananthapuram district will be invited to be screened for complications of diabetes including screening for DR. As the non-communicable diseases register is likely to show an increase in newly diagnosed diabetes as a result of training the accredited social health activists (ASHAs), in diabetes and DR, we will evaluate the effectiveness of the complex interventions delivered at each of the 16 family health centres for this study. The implementation

For peer review only - http://bmjopen.bmj.com/site/about/guidelines.xhtml

of the pathway will take into account all patients in the non-communicable diseases register at the start of the cross-sectional study.

Setting

Target sites for implementation of DR screening and treatment in Thiruvananthapuram will include 16 family health centres for DR screening which represents the primary care centres where patients are screened for all complications of diabetes. The retinal images, captured by the trained, resident nursing staff will be sent to a newly developed reading centre at the Regional Institute of Ophthalmology, a tertiary care centre, where newly accredited graders will grade the images. Patients with screen-positive images will be referred to three secondary care hospitals (district hospitals). Severe cases that require complex interventions will be referred to the tertiary care centre, the tertiary for specialist management of DR.

Description of the standard of care

Currently, patients with diabetes are not systematically screened for DR in the public health system. Most patients present to the tertiary centre voluntarily either because of increased awareness of complications of diabetes or due to visual impairment. Therefore, the current standard of care will be captured as the number of patients presenting to the tertiary centre for an eye consultation for DR over a period of time as there is no baseline data in the primary and secondary care. This cross-sectional survey of the patients presenting at the family health centres will provide information on the uptake of screening of people registered in the non-communicable diseases register at the start of the study. The prevalence of DR and STDR of the screened population will be estimated from the numbers screened in all the non-communicable diseases registers during this period.

Evidence-based clinical intervention:

The new DR care pathway is the intervention and is shown in Figure 1. The pathway will span primary, secondary and tertiary care. The components of the pathway are:

1. DR screening of patients with diabetes registered in the non-communicable diseases register at family health centres (primary care). The retinal images will be graded remotely at the reading centre at the tertiary centre.
2. Prompt referral for timely treatment of STDR to secondary care and tertiary centres depending on the severity of the DR.
3. Treatment of patients with sight threatening DR (at secondary and tertiary care).

For peer review only - http://bmjopen.bmj.com/site/about/guidelines.xhtml

Implementation strategies

The implementation strategies of the new DR care pathway are categorised as shown in Table 1 into plan, finance, education, infrastructure, quality improvement and policy contexts. These categories are developed based on the discrete Expert Recommendations for Implementing Change (ERIC) taxonomy.[20, 21] The logic model is shown in Figure 2.

Table 1: Implementation strategies

Categories of Implementation Strategies[21]	Discrete Implementation Strategy[20]	Specific actions
Plan	Conduct local needs assessment	The study team will conduct a situational analysis of data from self-referred patients.
	Assess for readiness and identify barriers and facilitators	Assess barriers that may impede implementation of the DR care pathway, and strengths that can be used in the implementation effort by interviewing key stakeholders i.e. family health centres staff, ophthalmologists in secondary and tertiary care, health service employees and patients.
	Develop a formal implementation blueprint	The blueprint will be used to guide the implementation of the whole DR care pathway and will be updated with time.
	Tailor strategies	Regular collaborative meetings will be held to tailor the implementation strategies to address barriers and leverage facilitators that are identified during various steps in the implementation.
	Stage implementation scale up and integration	The implementation will be staggered with five family health centres starting as pilots to enable small changes before gradually moving to other 11 centres. Addition of more centres during the staggered phase will be considered as sign of acceptability and feasibility.<br>Ensure integration of the DR care pathway into EHR. In the long-term, the adoption of the DR care pathway across the state of Kerala will be considered as success.
	Build a coalition	Cultivate relationships with staff at family health centres and secondary care centres, data entry operators, EHR team, project manager, and the Government of Kerala

For peer review only

For peer review only - http://bmjopen.bmj.com/site/about/guidelines.xhtml

		health service departmental staff in the implementation effort and create a learning system to incorporate practitioner feedback.
	Develop academic partnerships	Partner with UK academic institutions for the purposes of shared training and bringing research skills to the implementation of the pathway.
	Recruit, designate, and train for leadership	A project manager will be recruited to manage the programme under the supervision of the non-communicable diseases lead. The Health Secretary will conduct periodic meetings with the project team to assess performance.
	Obtain formal commitments	A collaborative agreement is in place between Government of Kerala and Moorfields Eye Hospital, London, UK outlining their roles and responsibilities in the implementation of the DR care pathway.
	Prepare patients to be active participants	Community level health workers will prepare patients to be active in their care, to ask questions, and specifically to inquire about care guidelines, the evidence behind clinical decisions, or about available evidence-supported treatments. Each patient will provide written consent to be screened for DR in this project as well as participate in interviews after verbal consent.
	Inform local opinion leaders	The Health Minister of the Kerala state and members of the local government will be briefed on the project and encouraged to be champions of the project to enable scale up to all family health centres in the state.
Finance	Access new funding	Use GCRF/UKRI funding to purchase smartphone retinal cameras and retinal lasers, develop EHR, train staff at family health centres and secondary care, employ project manager, data entry operators and personnel to conduct interviews, create study database linked to hand-held applications for data entry operators. State and local government funds will be sourced for scale up.
	Incentivisation	ASHAs will be incentivised to accompany participants with diabetes who are referred to secondary care for treatment.
Education	Develop educational materials	Develop DR training modules and retinal image capture modules for family health centres staff<br>Develop laser training material to train ophthalmologists.

For peer review only - http://bmjopen.bmj.com/site/about/guidelines.xhtml

	Distribute educational materials	Distribute educational materials (including guidelines, manuals, and toolkits) in person, by mail, and/or electronically. Multiple training sessions are planned at each family health centres. The camera manufacturers will be invited to provide hands-on training on capturing good quality retinal images. Training on laser surgery will also be provided to the ophthalmologists at secondary care.
	Conduct educational meetings	Hold meetings targeting different stakeholder groups (ophthalmologists, family health centres staff, ASHAs, patients). Three educational meetings are planned by the UK team to meet with the providers in the family health centres settings to share knowledge and educate providers about the DR care pathway and its integration into their clinical practice.
	Achieve accreditation for graders of retinal images	Train and certify the optometrists in the Reading Centre in the tertiary centre to obtain accreditation as graders using the online international Test and Training for retinal graders.
	Conduct ongoing training	Nurses and doctors in the family health centres will receive on-going training on DR, pupil dilatation and capture of retinal images using low-cost smartphone retinal cameras.[24, 25]
	Create a learning collaborative	The Health Secretary of State will foster a collaborative learning environment to improve implementation of the DR care pathway.
	Use train-the-trainer strategies	The UK team will first train a batch of family health centres nurses to capture retinal images and then some will be trained to train the rest of the nurses. The same cascading pathway will apply to laser training.
	Work with educational institutions	Moorfields Eye Hospital staff will provide the necessary guidance and share knowledge on UK diabetic retinopathy screening pathway.
	Make training dynamic	All staff involved will receive continual online or face to face training.
	Increase demand	The ASHAs will be trained to increase public awareness of this pathway in their house-to-house health visits.
	Provide ongoing consultation	The UK team from Moorfields Eye Hospital will provide ongoing consultation to support implementation of DR care pathway.

For peer review only - http://bmjopen.bmj.com/site/about/guidelines.xhtml

Infrastructure	Centralize technical assistance	The project manager and the EHR team will provide central assistance focused on implementation issues. The telemedicine project will link the patient's retinal images to EHR and transfer through a secure cloud to the tertiary centre, where the images will be graded for their DR status in a newly developed reading centre, with the results fed back to the family health centres.[26]
	Change physical structure and equipment	Evaluate current configuration of family health centres consulting rooms to allow suitable furniture for cameras and adapt illumination to capture retinal photographs.
	Change record systems	Change records systems to allow better assessment of implementation or clinical outcomes by having data entry operators interview patients and input full data into EHR within the busy family health centres clinics.
	Change service sites	Change the timetable for clinics in secondary care to allow a patients referred from primary care to access treatment.
	Revise professional roles	The non-communicable diseases lead will be delegated to take on the responsibility of implementing this DR care pathway in Thiruvananthapuram aiming for a Kerala state-wide roll out.
Quality Improvement	Develop and implement tools for quality monitoring	Develop, test, and introduce into quality-monitoring systems the right input—the appropriate language for the consent form, protocols on DR screening, grading retinal images, and measures of processes, patient outcomes, and implementation outcomes.
	Develop and organize quality monitoring systems	Develop and organise systems and procedures that monitor clinical processes and/or outcomes for the purpose of quality assurance and improvement.
	Audit and provide feedback	Collect and summarise clinical performance data every month and feedback to family health centres and secondary care staff to monitor, evaluate, and modify pathway for acceptability to patients and staff. A continuous monitoring of retinal image quality and gradability, and the referral pathway will be done to cyclically input change into the DR pathway to improve the quality and integration.
	Conduct cyclical small tests of change	Staggered set up of family health centres to allow implementation of changes in a cyclical fashion using small tests of change before

For peer review only - http://bmjopen.bmj.com/site/about/guidelines.xhtml

		taking changes system-wide. Tests of change benefit will be measured continually by increase uptake of training, screening, referral and treatment.
	Use data experts	The EHR team will develop an application for the data entry operators to collect data. The UK collaborators will collaborate in the analysis of the data.
	Use data warehousing techniques	Integrate clinical records from this research project into EHR on an on-going basis.
	Capture and share local knowledge	Capture local knowledge from implementation sites on how staff and doctors make something work in their family health centres and then share it with other centres.
	Obtain and use patients feedback	The participating patients will be asked to provide feedback on the implementation effort.
	Promote adaptability	Identify ways the DR care pathway can be tailored to meet local needs and clarify which elements of the pathway must be maintained to preserve fidelity.
	Provide clinical supervision	The Director of the tertiary centre will provide the overall clinical supervision of the whole DR care pathway. He will also delegate ophthalmologists to provide ongoing supervision for the optometrists involved in grading retinal images.
	Provide local technical assistance	Develop and use a system to deliver technical assistance focused on implementation issues using relevant service providers.
	Purposely re-examine the implementation	Monthly monitoring of screened patients at each family health centre, barriers observed and small changes that are required will be implemented to continuously improve the pathway.
	Remind clinicians	Primary care doctors in family health centres will receive monthly newsletter on the numbers recruited across all centres to highlighting successes and providing potential solutions to issues encountered.
Policy contexts	Issue Government of Kerala approval to enable dilatation of pupils of patients under supervision of family health centres doctors	Ensure doctors at family health centres receive approval for mydriasis in order to deliver the DR care pathway.

For peer review only - http://bmjopen.bmj.com/site/about/guidelines.xhtml

	Mandate change	The Health Secretary of State will provide leadership to ensure the prioritisation of the implementation of the DR care pathway.

DR-diabetic retinopathy; ASHAs -accredited social health activists; GCRF/UKRI -Global Challenge Research Fund/UK Research and Innovation; EHR –Electronic health records.

Outcomes

Pre-specified outcomes of the effectiveness of the DR pathway and the evaluation of the implementation strategy are tabulated in table 2 and table 3, respectively.

Table 2: DR pathway- (intervention) related outcomes

Outcomes	Indicators	Source of data
Clinical Effectiveness	– Proportion of people screened for DR in each family health centres over 12 months.<br>– Proportion of referred patients with STDR.<br>– Proportion of ungradable retinal images and percentage of cataract identified from referring these patients.<br>– Risk and complication burden in people screened for DR<br>– Presenting visual acuity of those referred from the DR pathway compared to those who self-referred to secondary care.<br>– Generalisability of the prevalence data compared to house-to-house survey in a neighbouring district.	Data from monthly entries into EHR and study database.<br>–
Safety	Data on complications of the DR pathway	EHR and study database

For peer review only

Cost-effectiveness	The key outcome is the number of cases of severe visual impairment or blindness due to STDR averted as a result of the new DR care pathway and resultant QALY gain.<br>Estimated QALY gain from laser treatment of STDR and from cataract surgery, using utility values from Rachapelle et al 2013.[27]<br>Assumption that the effect of no screening is as in historic studies that reported the rate at which STDR leads to blindness if left untreated.	Data collected in the study, data from the literature and expert opinion where data could not be collected.
Efficiency	Efficiency is defined as optimal use of the service. For this purpose, we will first estimate the time required to process one patient from the path-process analysis. This estimate will be used to determine the number of patients who can be seen on a day. We will then calculate (1- number of patients screened)/number of patients that can be screened). proportion can be used in a p-type control chart.[28]	Capture efficiencies in structures, resources and processes across the DR care pathway from staff interviews.<br><br>Path process mining of patients screened in family health centres analysis will be based on detailed time-event logs of approximately 70 patients from the 16 FHCs
Patient satisfaction	Patients willingness to be screened and their satisfaction with the information provided to them and the care they received	Patient interviews and telephone survey of patients referred to secondary care for treatment
Timeliness	Path process mapping at family health centres to understand delays in pathway Reasons for non-attendance for those referred to secondary care for treatment of STDR	Path process mapping at family health centres to understand delays in DR care pathway<br><br>Patient survey and secondary care records
Equity	Age and gender-based prevalence of DR and STDR will be reported on consented patients from all family health centres. Reasons of non-referral of patients that require referrals will be recorded. Numbers denied screening despite willingness to be screened will also be reported.	Data from EHR and study database.

For peer review only - http://bmjopen.bmj.com/site/about/guidelines.xhtml

256 DR -diabetic retinopathy; STDR-sight threatening diabetic retinopathy; QALY- quality-adjusted life year

259 **Table 3: Outcomes of the evaluation of the implementation strategy**

Outcomes	Indicators	Assessment and data source
Acceptability	Willingness of each family health centres to be involved in the DR care pathway.	**Assessment:** Readiness to implement the DR care pathway in the 16 family health centres and any reasons for delay.<br>*Data source:* Clinical performance from family health centres that will include monthly uptake of DR screening at each centre and any reports on barriers causing delays in implementation provided by the project manager.
	Staff willingness to train	**Assessment:** Proportions of trained ASHAs, nurses and doctors per family health centres, increase in doctors trained in laser surgery in secondary care, proportions willing to be included in train the trainer programme. The denominator will be total numbers of each personnel available to be trained on the date of implementation.<br>*Data sources:* Accreditation and certification records; Structured interviews of at least 5 nurses and 5 primary care doctors from 16 family health centres, 3-5 ophthalmologists from secondary and tertiary care, 2 data entry operators and 5 ASHAs and 1 health service administrator to understand challenges for acceptance.
	Patients acceptability of the pathway	**Assessment:** Screening attendances per family health centres over 12 months.<br>*Data sources:* Clinical performance data and a telephone questionnaire survey for screened and referred patients to evaluate reasons for attendance and non-attendance.
Adoption	Uptake of DR care pathway at primary, secondary and tertiary care<br>Uptake of training by staff	**Assessment:** Proportion of staff at primary, secondary and tertiary care willing to integrate DR care pathway in their workload.<br>*Data source:* family health centres data and staff interviews

For peer review only - http://bmjopen.bmj.com/site/about/guidelines.xhtml

	Uptake of screening programme by patients<br><br>Adoption of DR care pathway by the Government of Kerala for state-wide implementation.	**Assessment:** Demand training sessions; Attendance rates at training sessions<br><br>*Data source:* number of training sessions and attendance registers at training sessions.<br><br>**Assessment:** Increase in proportions of patient screened over 12 months.<br><br>*Data source*: EHR and study database.<br><br>**Assessment:** A policy paper by the Government of Kerala for state-wide adoption of the DR care pathway as part of diabetes care for all patients.<br><br>*Data source:* A detailed scale-up plan will be developed for each district based on the clinical and cost-effectiveness data from this study to inform policy.
Appropriateness	Appropriateness of family health centres for DR screening<br><br>Appropriateness of the complex DR care pathway across primary and secondary care<br><br>Appropriateness of referrals to secondary care	**Assessment:** Qualitative data on barriers and facilitators of the delivery of screening and treatment in secondary care.<br><br>*Data sources:* interview of staff at family health centres and secondary care, pPatient telephone survey on pathway from FHCs to secondary care<br><br>**Assessment:** False positive referrals from data collection at secondary care.<br><br>*Data sources:* study database; data from patient telephone survey
Cost	Costs to the Government of Kerala and societal costs of the DR screening programme and subsequent treatment	**Assessment:** Costs of the DR screening and treatment costs in total and per person, including the costs of staff training and cameras (suitably annuitized), staff costs and travel costs of patients.<br><br>*Data sources:* Data collected in the study baseline and data from other services in India and from the literature.
Feasibility	Screening rate per family health centres Barriers to monthly screening uptake	**Assessment:** Monthly increase in numbers of FHCs added to the programme

For peer review only - http://bmjopen.bmj.com/site/about/guidelines.xhtml

		Data source:Interview and monthly report from project manager on facilitators and barriers on each part of the pathway
Fidelity	Degree of the DR care pathway implementation<br><br>Validation of the DR grading within the telemedicine pathway<br><br>Delivery of training and the impact on the DR care pathway	**Assessment:** Fidelity evaluation in 16 family health centres using a path-process analysis of the screening to identify the common screening pathway across centres .<br><br>*Data source*: The path-process analysis will be based on detailed time-event logs of 70 patients from the 16 family health centres; a telephone follow-up of referred patients to evaluate uptake of referrals.<br>**Assessment:** Validation of the DR grading will be assessed by the agreement of the DR grade reported by the optometrists at the reading centre in the tertiary centre and those reported by the ophthalmologists in the secondary care centres.<br><br>*Data sources*: DR grading of referred patients obtained from the reading centre versus secondary care.<br><br>**Assessment:** Increase in numbers of trained staff per month.<br><br>*Data sources:* Staff interviews, structured observations and review of facility records. The proportions of doctors trained on laser delivery; data quality on the database; Increase in non-communicable diseases registration following start of programme due to training of ASHA workers on diabetes and DR; Increase in numbers of patients treated for STDR following training.
Penetration	Increase patient awareness of diabetes and DR and uptake of the DR screening pathway in patients with diabetes	**Assessment**: Increase uptake of DR screening by patients registered in non-communicable diseases registers.<br><br>*Data sources*: from non-communicable diseases register and EHR
Sustainability	Policy makers to prepare policy on up-scale and sustainability plan for DR pathway	**Assessment:** Availability of a policy paper from Government of Kerala on state-wide scale up of the DR care pathway. This will be considered a success of the programme.<br><br>*Data sources*: Scale up plan for other districts including plan to purchase retinal cameras and

For peer review only

	Integration of DR pathway in weekly activities in FHCs	acceptance of guidelines and training material for state-wide roll out.<br><br>**Assessment:** Integration of DR pathway in EHR<br><br>*Data source:* EHR records of DR screening as part of non-communicable diseases care pathway.

DR-diabetic retinopathy; ASHA -accredited social health activists, EHR- electronic health records

Data collection

Quantitative data

Effectiveness outcomes will originate from the data sources shown in table 2. Data collected by nurses or data operators from the EHR include age, gender, duration of diabetes, use of insulin, parental history of diabetes, other complications of diabetes including diabetic kidney disease, cardiovascular complications, and diabetic foot, random blood sugar results, urine dipstick test for albuminuria and blood pressure record. Other study-specific data collected by nurses or data operators on the day of screening include education status, occupation and income categories, and previous history of DR, cataract surgery or any other ocular history. In addition, they will measure body mass index, waist circumference and complete a lifestyle questionnaire on smoking, diet, physical activity, EQ-5D vision bolt-on[29]. The EQ-5D vision bolt-on will be used to calculate the quality adjusted life-years and utility value for economic analysis. EQ-5D alone does not capture visual acuity deficits.[30] The EQ-5D vision bolt-on asks patients to rate their health across 6 dimensions: mobility, self-care, usual activities, pain/discomfort, anxiety/depression and vision. Each dimension is scored in 5 levels: no problems, slight problems, moderate problems, severe problems, and extreme problems. A recent study provided utility values based on EQ-5D vision bolt-on. The mapping was done in a clinical trial cohort with macular oedema in central retinal vein occlusion. [31]

In the reading centre, the data collection will include the grade of retinopathy in both eyes, presence of cataract and gradability of the retinal images. Data collected on referred patients will include numbers with ungradable images due to cataract, treatment options offered for DR and review appointment.

For peer review only - http://bmjopen.bmj.com/site/about/guidelines.xhtml

Qualitative data collection and analysis

Data sources used for evaluation of the implementation strategy are shown in table 3. These will include data from a structured interview of a maximum variable sample that will reflect the context (primary, secondary and tertiary care), and the functions and skill levels of the staff (e.g. nurses, doctors etc.). Based on pragmatic considerations, at least 5 nurses and 5 primary care doctors from 16 FHCs, 3-5 ophthalmologists from secondary and tertiary care, 2 data entry operators and 5 ASHAs and 1 health service administrator should be included to get the maximum variability. Verbal consent will be obtained from these health professionals. The interviews will be conducted by an independent member from the GCRF/UKRI-funded team, who is not involved in this study, in the local language within the premises of the healthcare provider. The focus group will consist of groups of patients and staff within one family health centres. In addition, to the voice-recording of the interviews and focus group, interviewers will write field notes to describe the interview situation. Field notes, the interviews and focus group content will provide the basis for the data analysis, which will be based on a descriptive phenomenological approach without data or opinion interpretation and will include transcription, condensation, coding and categorisation using qualitative analysis tools. A survey of all referred patients will be done using a structured questionnaire to evaluate their satisfaction and their perception of the barriers and facilitators. All qualitative data will be coded using NVivo and analysed using descriptive phenomenological approach following the strategy.[32] We will transcribe all the participants' descriptions, extract significant statements or phrases, create formulated meanings or meaning units, aggregate formulated meanings and incorporate the result into descriptions.

Data Monitoring

Data will be coded before entry into the study database by the clinical teams. Only anonymised extracted data from EHR by the nurses or data entry operators will be used for analysis. Data quality will be monitored by the study project manager. Anonymised data will be checked for range checks and data quality at University of East London by the study statistics team. The ORNATE India International Advisory Board will have an overview of the conduct of the project and the Executive Group consisting of the UK-India collaborators will monitor the conduct of the study.

Sample size

For peer review only - http://bmjopen.bmj.com/site/about/guidelines.xhtml

We have chosen the proportion of patients in the non-communicable diseases register with diabetes screened for DR as our primary outcome variable.

Justification of the choice of primary outcome: This variable captures the effectiveness of our intervention as well as the fidelity of implementation. Other facts that informed our choice of the primary outcome is that we expect a short implementation time of 7 to 9 months during which other outcomes such as number of patients treated may not be a feasible option. Therefore, we have estimated the sample size based on this primary outcome.

Our calculations are complicated by the expected increase in number of people with diabetes in the non-communicable diseases register as public awareness of diabetes and DR increases so the denominator of numbers screened will be the number of people registered in the non-communicable diseases in each family health centres at the start of the cross-sectional study. There is no data on the baseline proportion of patients that attend the tertiary centre for screening. The prevalence of diabetes is between 10-16% in Thiruvananthapuram of which 8% are estimated to have DR and 3% to have STDR but about 20% may have to be referred. Assuming a finite population of 40,000 patients with diabetes, a simple random sample of 377 patients will be needed. However, we expect large design effects due to clustering patients within family health centres. There is not enough data to calculate the value of this design effect and therefore we assume it to be 3 to give a final sample size of 1,131, which is equivalent to assuming a within-family health centres intracluster correlation coefficient of approximately 0.03 (n=16 family health centres) with negligible residual clustering by the intermediate cluster level of ASHAs, where mean cluster size is smaller.

As described by Becker et al in mammography screening,[33] we expect sources of bias in the implementation programme that will likely influence the sample size calculation. Unscreened DR cases that already exist in the community may contribute to an over-estimation of the effect of screening. We expect lead-time bias because of our short implementation period and so some DR in the community may be missed. A comparison of numbers with DR in family health centres with longer implementation period with those of shorter period may adjust for this bias. We intend to provide a descriptive analysis as well as a temporal comparison using time series analysis.

Statistical Analyses

We will create a complete case dataset of the cross-sectional survey for use in the analyses of effectiveness and assess the potential impact of missing data using sensitivity analysis incorporating a range of optimistic and pessimistic scenarios for the impact of missing data. We

For peer review only

will model two outcomes that we hypothesised would be influenced by the intervention: (1) uptake of DR screening (primary outcome) and (2) numbers of patients referred for STDR as a result of screening. We will use bivariate logistic regression models to assess crude associations between sociodemographic factors such as age, gender, education, income, and living arrangements, and other clinical data with DR and STDR and use fractional polynomials to account for the form of continuous covariates to predict uptake of DR screening. Factors associated with a P<0.25 will be candidates for inclusion in the multivariable logistic regression models. We will test interactions amongst identified main effects to capture improvement in model prediction assessed by reduced residual variance-based statistics. To account for the staggered entry into the study, we will add a variable indicating the month of entry of each family health centres into the programme (e.g., 1. if the family health centre join in the first month of the programme, 2. if in the second month and so on). A non-significant coefficient for this variable will suggest the staggered approach had no effect and a significant coefficient will allow the effect to be quantified for each family health centre. In our final adjusted model, we will consider associations with a P<.05 as statistically significant. Adequacy of model discrimination and calibration will be assessed using Receiver Operating Characteristic (ROC) curves. The validation of the telemedicine will be reported as the agreement (kappa statistics) between screen positive patients graded by the graders at the reading centre versus the DR grade as recorded by the ophthalmologists in secondary care centres. A kappa coefficient of 0.6 or higher was pre-specified to indicate validity.

Patient and public involvement (PPI)

A patient and public group will be involved in plans to disseminate the study results and provide their input on the scale-up of this DR care pathway, should the implementation in Thiruvananthapuram be deemed successful by the Government of Kerala.

Ethics and dissemination

The study was approved by Indian Medical Research Council (2018-0551). The study complies with the guidelines of the Declaration of Helsinki. Results of this research are expected to be disseminated through stakeholder reports and via scientific forums, specifically peer-reviewed publications and conference presentations. All participants will give written informed consent prior to entry to the study by the family health centres nurses and will be made aware that participation is strictly voluntary.

For peer review only

For peer review only - http://bmjopen.bmj.com/site/about/guidelines.xhtml

Discussion

At the conclusion of this study, we hope to assess the effectiveness and implementation outcomes of a complex DR care pathway integrating care at primary, secondary and tertiary care, covering a proportion of the diabetic population in Thiruvananthapuram. The study will set the scene for a policy for State-wide Screening and Treatment Pathway for Diabetes. Early identification of STDR and other complications due to this holistic approach and timely treatment, are expected to have a positive impact on rates of blindness, chronic kidney disease, cardiovascular complications and thereby improve health, reduce multi-morbidity and mortality. A DR pathway that straddles primary, secondary and tertiary levels of care, leveraging technology may have advantages of cost effectiveness and ease of implementation in low and middle income countries compared to the current practice of detection and management of self-reported cases in tertiary centres.

This study outlines how the effectiveness of a DR care pathway and its implementation will be evaluated. [21, 22] This study is timely given the increasing numbers of people with diabetes and pressure on finances available for healthcare, necessitating task shifting to enable better coverage of the population.. The study will also examine the organisation functions such as structure, resources and processes that would contribute to better outcomes. We will also examine whether this additional task is feasible given the current skill levels and workload of family health centres and secondary hospitals. The study will reveal whether current health seeking behaviour of patients will support screening, especially when an invasive procedure is requested when there is no obvious impact on quality of life of the patient. By addressing a key gap in knowledge due to lack of research in this area, we will be able to decipher the barriers and facilitators that influence the successful implementation of such a programme in the public system in India. The results of this study may inform the adoption of this pathway in other areas in India and globally. However, the complexity and number of implementation strategies, local contextual factors and lack of validated implementation outcomes may limit generalisability of the results and implementation of this pathway elsewhere.

There are limitations, however, to the study design. We do not have baseline data on DR screening in the study location as these screening programmes are non-existent. For this reason, we are examining the effectiveness of the pathway in terms of presenting visual acuity for referable cases as 'proximal' outcomes. In addition, when there is no concurrent control group, causal inferences are difficult to make and may lead to both measured and unmeasured biases. When examining implementation, logistical issues, variations in health care facilities,

For peer review only - http://bmjopen.bmj.com/site/about/guidelines.xhtml

socio-economic variation, and quality of health care personnel may likely affect our study. However, the study design avoids the ethical challenges of having a control group with no DR screening. Therefore, the study design is by necessity non-randomised and observational and will rely on newly trained staff members to collect data, which is likely to differ in completeness between the 16 family health centres . We expect an increase in referral rates after implementation of the intervention due to better public awareness, increasing knowledge of ASHAs and improved case ascertainment, at least in some family health centres . We will also examine the impact of cataract in the community and this has not been studied before in any other DR pathway. We have tried to minimise the limitations by the use of robust statistical techniques and the use of various data sources to elicit a greater understanding of how the programme will lead to better health outcomes.

One of the strengths of this study is that quantitative data backed by qualitative data will be collected to strengthen our findings and enable generalisation of our findings. Secondly, we will use robust statistical methods to reduce bias including selection bias and other confounders. Finally, the access to and collaboration with the UK is a key strength of the study, as it facilitates the co-development of the interventions from the outset. Active involvement of policy makers engaged in transformation of primary care to screen for and address complications of non-communicable diseases who value research to generate evidence for policy making and who are prepared to learn from, and adapt the DR care pathway based on implementation experience is a unique feature of this study.. Findings on the mechanisms and contexts that optimise the implementation of this complex multi-faceted intervention using the ERIC taxonomy will be useful to those developing and implementing these programmes in other health systems. The health economic model may highlight the health expenditure required at individual, family and Kerala State level for forecasting and planning health budgets.

Perhaps the most important aspect of the chosen evaluation is that it is built within a simultaneous developing public health strategy on population-based screening of diabetes and hypertension and a recently introduced EHR called eHealth. Conducting research in such an environment is a good example of health policy and systems research.

Despite the limitations, this study holds promise for providing high-quality data and detailed implementation information on a complex intervention in a resources-limited setting. We hope to

For peer review only - http://bmjopen.bmj.com/site/about/guidelines.xhtml

contribute to the literature on the implementation and effectiveness of DR screening and treatment in the public health sector in low and middle income countries.

Author Contributorship

Sobha Sivaprasad (S.S), Gopalakrishnan Netuveli (G.N), Raphael Wittenberg (R.W), Rajan Khobragade (R.K), Rajeev Sadanandan (R.S), Bipin Gopal (B.G), Lakshmi Premnazir (L.P), Dolores Conroy (D.C), Jyotsna Srinath (J.S), Radha Ramakrishnan (R.R), Simon George (S.G), Vasudeva Iyer Sahasranamam (V.S)

Conceptualisation, S.S, G.N, R.W, R.S, B.G, S.G, V.S; methodology, S.S, G.N, R.R, R.S, B.G, V.S, S.G, D.C, R.R, R.K; acquisition of data, S.S, G.N, R.W, D.C, R.R, J.S, L.P, B.G; writing—original draft preparation, S.S, D.C, G.N, R.W, L.P, R.R; writing—review and editing, S.S, G.N, R.R, R.S, B.G, V.S, S.G, D.C, R.R, R.K, J.S, L.P; funding acquisition, S.S, G.N, R.W, R.S, on behalf of the Collaborators.

Role of study sponsor and funders, if any, in study design; collection, management, analysis, and interpretation of data; writing of the report; and the decision to submit the report for publication, including whether they will have ultimate authority over any of these activities; None

Role of sponsor: The Director of Regional Institute of Ophthalmology contributed to study design; management, writing of the report; and the decision to submit the report for publication, but have no authority over any of these activities.

Authorship eligibility guidelines will be based on ICJME and there is no intention to use professional writers.

Acknowledgements:
Nayanamritham Project Collaborators: Dr Rajeevan Palpoo and Dr Chitra Raghavan will manage the referred patients in Regional Institute of Ophthalmology; Alwin Aniyankunju and Anju V.Molly will be responsible for grading the retinal images.
Steering Committee: Members of the International Advisory Board from Kerala

Funding statement: This work is part of the ORNATE India project funded by Global Challenges Research Fund and UK Research and Innovation through the Medical Research Council grant number MR/P027881/1

Data Sharing Agreement: The technical appendix, statistical code and dataset will be made available on request after obtaining permission from the Government of Kerala.

Competing interests statement: None

For peer review only

Sponsor: Dr V Sahasranamam, Director, Regional Institute of Ophthalmology, Thiruvananthapuram, Kerala

References

1. International Diabetes Federation. *IDF Diabetes Atlas*. 9th edition 2019 https://www.diabetesatlas.org/en/
2. Yau JW, Rogers SL, Kawasaki R, et al. Meta-Analysis for Eye Disease (META-EYE) Study Group. Global prevalence and risk factors of diabetic retinopathy. *Diabetes Care* 2012; 35:556–64.
3. IDF Diabetes Atlas: Global estimates of diabetes prevalence for 2017 and projections for 2045. Cho NH, Shaw JE, Karuranga S, Huang Y, da Rocha Fernandes JD, Ohlrogge AW, Malanda B Diabetes Res Clin Pract. 2018; 138():271-281.
4. Scanlon PH. The English National Screening Programme for diabetic retinopathy 2003-2016. *Acta Diabetol* 2017; 54(6):515-25
5. Cavan D, Makaroff L, da Rocha Fernandes J, et al. The Diabetic Retinopathy Barometer Study: Global perspectives on access to and experiences of diabetic retinopathy screening and treatment. *Diabetes Res Clin Pract* 2017; 129:16-24.
6. NITI Ayog (2020), SDG India Index, Baseline Report, 2018, New Delhi [Last accessed on 2021 Jan 17]. Available from: www.niti.gove.in
7. Menon VU, Kumar KV, Gilchrist A, et al. Prevalence of known and undetected diabetes and associated risk factors in central Kerala--ADEPS. *Diabetes Res Clin Pract* 2006; 74:289–94.
8. Sustainable Development Goals. United Nations. [Last accessed on 2020 May12]. Available from: http://www.un.org/sustainabledevelopment/sustainable-developmentgoals/
9. The WHO STEPwise approach to Surveillance of noncommunicable diseases (STEPS). Noncommunicable Diseases and Mental Health. World Health Organization. 20 Avenue Appia, 1211 Geneva 27, Switzerland.
10. Scanlon PH, Aldington SJ, Leal J, et al. Development of a cost-effectiveness model for optimisation of the screening interval in diabetic retinopathy screening. *Health Technol Assess* 2015;19(74):1-116.
11. Leese GP, Morris AD, Olson J. A national retinal screening programme for diabetes in Scotland. *Diabet Med* 2003;20(12):962-4.
12. Lund SH, Aspelund T, Kirby P et al. Individualised risk assessment for diabetic retinopathy and optimisation of screening intervals: a scientific approach to reducing healthcare costs. *Br J Ophthalmol*. 2016;100(5):683-7.
13. Manne-Goehler J, Geldsetzer P, Agoudavi K et al. Health system performance for people with diabetes in 28 low- and middle-income countries: A cross-sectional study of nationally representative surveys. *PLoS Med*. 2019;16(3):e1002751.
14. Ting DS, Cheung GC, Wong TY. Diabetic retinopathy: global prevalence, major

For peer review only - http://bmjopen.bmj.com/site/about/guidelines.xhtml

risk factors, screening practices and public health challenges: a review. *Clin Exp Ophthalmol.* 201;44(4):260-77.

15. Hazin R, Barazi MK, Summerfield M. Challenges to establishing nationwide diabetic retinopathy screening programs. *Curr Opin Ophthalmol*. 2011;22(3):174-9.

16. Burgess PI, Msukwa G, Beare NA. Diabetic retinopathy in sub-Saharan Africa: meeting the challenges of an emerging epidemic. *BMC Med*. 2013;11:157.

17. Ramke J, Petkovic J, Welch V et al. Interventions to improve access to cataract surgical services and their impact on equity in low- and middle-income countries. *Cochrane Database Syst Rev*. 2017;11(11):CD011307.

18. Rani PK, Bhattarai Y, Sheeladevi S et al. Analysis of yield of retinal imaging in a rural diabetes eye care model. *Indian J Ophthalmol*. 2018 Feb;66(2):233-237.

19. Scanlon PH, Foy C, Malhotra R, Aldington SJ. The influence of age, duration of diabetes, cataract, and pupil size on image quality in digital photographic retinal screening. *Diabetes Care*. 2005;28(10):2448-53.

20. Powell BJ, Waltz TJ, Chinman MJ et al. A refined compilation of implementation strategies: results from the Expert Recommendations for Implementing Change (ERIC) project. *Implement Sci*. 2015;10:21.

21. Powell BJ, McMillen JC, Proctor EK, et al. A compilation of strategies for implementing clinical innovations in health and mental health. *Med Care Res Rev*. 2012;69(2):123-157.

22. Smith JD, Li DH, Rafferty MR. The Implementation Research Logic Model: a method for planning, executing, reporting, and synthesizing implementation projects. *Implement Sci*. 2020 Sep 25;15(1):84.

23. Landes SJ, McBain SA, Curran GM. Reprint of: An introduction to effectiveness-implementation hybrid designs. *Psychiatry Res*. 2020 Jan;283:112630.

24. Rajalakshmi R, Subashini R, Anjana RM, et al. Automated diabetic retinopathy detection in smartphone-based fundus photography using artificial intelligence. *Eye* (Lond) 2018;32(6):1138-1144

25. Piyasena MMPN, Murthy GVS, Yip JLY et al. Systematic review and meta-analysis of diagnostic accuracy of detection of any level of diabetic retinopathy using digital retinal imaging. *Syst Rev*. 2018 Nov 7;7(1):182. doi: 10.1186/s13643-018-0846-y. Erratum in: Syst Rev. 2019 Apr 30;8(1):106.

26. Boucher MC, Nguyen MTD, Qian J. Assessment of Training Outcomes of Nurse Readers for Diabetic Retinopathy Telescreening: Validation Study. *JMIR Diabetes*. 2020;5(2):e17309.

27. Rachapelle S, Legood R, Alavi Y, Lindfield R, Sharma T, Kuper H, Polack S. The cost-utility of telemedicine to screen for diabetic retinopathy in India. *Ophthalmology*. 2013 Mar;120(3):566-573.

For peer review only

28. NIST/SEMATECH e-Handbook of Statistical Methods. Proportion Control Chart https://www.itl.nist.gov/div898/handbook/pmc/section3/pmc332.htm, Accessed 5/1/2021

29. Finch A.P., Brazier J.E., Mukuria C., Bjorner J.B. An exploratory study on using principal-component analysis and confirmatory factor analysis to identify bolt-on dimensions: the EQ-5D case study. *Value Health.* 2017;**20**(10):1362–1375.

30. Tosh J, Brazier J, Evans P, Longworth L. A review of generic preference-based measures of health-related quality of life in visual disorders. Value Health. 2012 Jan;15(1):118-27.

31. Pennington BM, Hernández-Alava M, Hykin P, et al. Mapping From Visual Acuity to EQ-5D, EQ-5D With Vision Bolt-On, and VFQ-UI in Patients With Macular Edema in the LEAVO Trial. *Value Health*. 2020;23(7):928-935.

32. Colaizzi PF. Psychological research as the phenomenologist views it. In: Valle R, editor. KM, New York: Oxford University Press; 1978.

33. Becker N, Hakama M, Nyström L. Evaluation of effectiveness of quality-assured mammography screening in Germany: sample size considerations and design options. *Eur J Cancer Prev* 2007;16(3):225-31.

Legend: Figure 1: Proposed Diabetic Retinopathy Care Pathway

Figure 2: The Implementation Research Logic Model

For peer review only - http://bmjopen.bmj.com/site/about/guidelines.xhtml

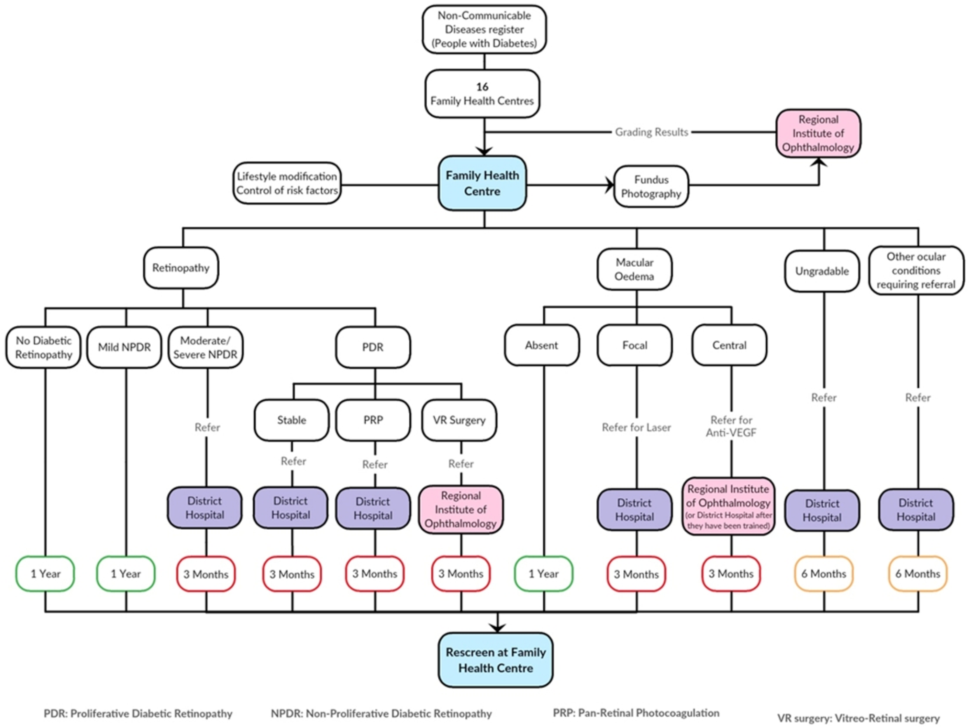

Figure !

186x138mm (300 x 300 DPI)

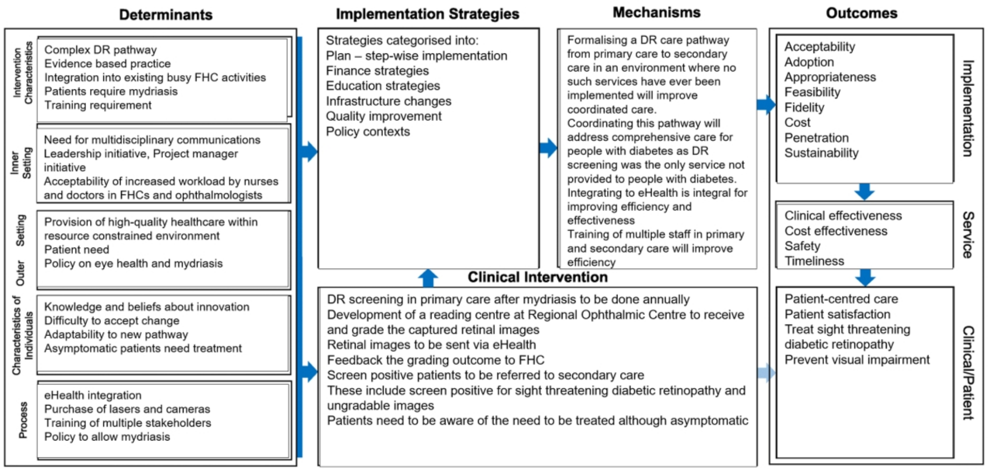

Figure 2 The Implementation Research Logic Model

188x90mm (300 x 300 DPI)

SPIRIT
STANDARD PROTOCOL ITEMS: RECOMMENDATIONS FOR INTERVENTIONAL TRIALS

SPIRIT 2013 Checklist: Recommended items to address in a clinical trial protocol and related documents*

Section/item	Item No	Description	Addressed on page number
**Administrative information**			
Title	1	Descriptive title identifying the study design, population, interventions, and, if applicable, trial acronym	_ 1____________
Trial registration	2a	Trial identifier and registry name. If not yet registered, name of intended registry	__1___________
	2b	All items from the World Health Organization Trial Registration Data Set	__NA__________
Protocol version	3	Date and version identifier	_1____________
Funding	4	Sources and types of financial, material, and other support	__25_____
Roles and responsibilities	5a	Names, affiliations, and roles of protocol contributors	__24__________
	5b	Name and contact information for the trial sponsor	__25__________
	5c	Role of study sponsor and funders, if any, in study design; collection, management, analysis, and interpretation of data; writing of the report; and the decision to submit the report for publication, including whether they will have ultimate authority over any of these activities	__25__________
	5d	Composition, roles, and responsibilities of the coordinating centre, steering committee, endpoint adjudication committee, data management team, and other individuals or groups overseeing the trial, if applicable (see Item 21a for data monitoring committee)	__19_________

For peer review only

For peer review only - http://bmjopen.bmj.com/site/about/guidelines.xhtml

Introduction

Background and rationale	6a	Description of research question and justification for undertaking the trial, including summary of relevant studies (published and unpublished) examining benefits and harms for each intervention	__4_and 5
	6b	Explanation for choice of comparators	__6___________
Objectives	7	Specific objectives or hypotheses	__6__________
Trial design	8	Description of trial design including type of trial (eg, parallel group, crossover, factorial, single group), allocation ratio, and framework (eg, superiority, equivalence, noninferiority, exploratory)	___6___________

Methods: Participants, interventions, and outcomes

Study setting	9	Description of study settings (eg, community clinic, academic hospital) and list of countries where data will be collected. Reference to where list of study sites can be obtained	___7___________
Eligibility criteria	10	Inclusion and exclusion criteria for participants. If applicable, eligibility criteria for study centres and individuals who will perform the interventions (eg, surgeons, psychotherapists)	___7 _________
Interventions	11a	Interventions for each group with sufficient detail to allow replication, including how and when they will be administered	___7___________
	11b	Criteria for discontinuing or modifying allocated interventions for a given trial participant (eg, drug dose change in response to harms, participant request, or improving/worsening disease)	____7-8_______
	11c	Strategies to improve adherence to intervention protocols, and any procedures for monitoring adherence (eg, drug tablet return, laboratory tests)	_____18________
	11d	Relevant concomitant care and interventions that are permitted or prohibited during the trial	_____NA_______ _
Outcomes	12	Primary, secondary, and other outcomes, including the specific measurement variable (eg, systolic blood pressure), analysis metric (eg, change from baseline, final value, time to event), method of aggregation (eg, median, proportion), and time point for each outcome. Explanation of the clinical relevance of chosen efficacy and harm outcomes is strongly recommended	table 2 and 3

For peer review only - http://bmjopen.bmj.com/site/about/guidelines.xhtml

Participant timeline	13	Time schedule of enrolment, interventions (including any run-ins and washouts), assessments, and visits for participants. A schematic diagram is highly recommended (see Figure)	Figure 1
Sample size	14	Estimated number of participants needed to achieve study objectives and how it was determined, including clinical and statistical assumptions supporting any sample size calculations	20
Recruitment	15	Strategies for achieving adequate participant enrolment to reach target sample size	table 1

Methods: Assignment of interventions (for controlled trials)

Allocation:

Sequence generation	16a	Method of generating the allocation sequence (eg, computer-generated random numbers), and list of any factors for stratification. To reduce predictability of a random sequence, details of any planned restriction (eg, blocking) should be provided in a separate document that is unavailable to those who enrol participants or assign interventions	_NA_________
Allocation concealment mechanism	16b	Mechanism of implementing the allocation sequence (eg, central telephone; sequentially numbered, opaque, sealed envelopes), describing any steps to conceal the sequence until interventions are assigned	NA________
Implementation	16c	Who will generate the allocation sequence, who will enrol participants, and who will assign participants to interventions	NA_______
Blinding (masking)	17a	Who will be blinded after assignment to interventions (eg, trial participants, care providers, outcome assessors, data analysts), and how	NA_________
	17b	If blinded, circumstances under which unblinding is permissible, and procedure for revealing a participant's allocated intervention during the trial	NA________

Methods: Data collection, management, and analysis

Data collection methods	18a	Plans for assessment and collection of outcome, baseline, and other trial data, including any related processes to promote data quality (eg, duplicate measurements, training of assessors) and a description of study instruments (eg, questionnaires, laboratory tests) along with their reliability and validity, if known. Reference to where data collection forms can be found, if not in the protocol	_20-21____

	18b	Plans to promote participant retention and complete follow-up, including list of any outcome data to be collected for participants who discontinue or deviate from intervention protocols	____8-12______
Data management	19	Plans for data entry, coding, security, and storage, including any related processes to promote data quality (eg, double data entry; range checks for data values). Reference to where details of data management procedures can be found, if not in the protocol	____17-18__
Statistical methods	20a	Statistical methods for analysing primary and secondary outcomes. Reference to where other details of the statistical analysis plan can be found, if not in the protocol	____ 21_______
	20b	Methods for any additional analyses (eg, subgroup and adjusted analyses)	______21______
	20c	Definition of analysis population relating to protocol non-adherence (eg, as randomised analysis), and any statistical methods to handle missing data (eg, multiple imputation)	____NA________ _
**Methods: Monitoring**			
Data monitoring	21a	Composition of data monitoring committee (DMC); summary of its role and reporting structure; statement of whether it is independent from the sponsor and competing interests; and reference to where further details about its charter can be found, if not in the protocol. Alternatively, an explanation of why a DMC is not needed	____19________
	21b	Description of any interim analyses and stopping guidelines, including who will have access to these interim results and make the final decision to terminate the trial	____NA________ _
Harms	22	Plans for collecting, assessing, reporting, and managing solicited and spontaneously reported adverse events and other unintended effects of trial interventions or trial conduct	_____NA_______ _
Auditing	23	Frequency and procedures for auditing trial conduct, if any, and whether the process will be independent from investigators and the sponsor	____NA________ _
**Ethics and dissemination**			
Research ethics approval	24	Plans for seeking research ethics committee/institutional review board (REC/IRB) approval	______22_____

Protocol amendments	25	Plans for communicating important protocol modifications (eg, changes to eligibility criteria, outcomes, analyses) to relevant parties (eg, investigators, REC/IRBs, trial participants, trial registries, journals, regulators)	___1__________
Consent or assent	26a	Who will obtain informed consent or assent from potential trial participants or authorised surrogates, and how (see Item 32)	____22__________
	26b	Additional consent provisions for collection and use of participant data and biological specimens in ancillary studies, if applicable	____NA_________
Confidentiality	27	How personal information about potential and enrolled participants will be collected, shared, and maintained in order to protect confidentiality before, during, and after the trial	_____19
Declaration of interests	28	Financial and other competing interests for principal investigators for the overall trial and each study site	___25___________
Access to data	29	Statement of who will have access to the final trial dataset, and disclosure of contractual agreements that limit such access for investigators	____25__________
Ancillary and post-trial care	30	Provisions, if any, for ancillary and post-trial care, and for compensation to those who suffer harm from trial participation	____NA_________ _
Dissemination policy	31a	Plans for investigators and sponsor to communicate trial results to participants, healthcare professionals, the public, and other relevant groups (eg, via publication, reporting in results databases, or other data sharing arrangements), including any publication restrictions	____22__________
	31b	Authorship eligibility guidelines and any intended use of professional writers	____25__________
	31c	Plans, if any, for granting public access to the full protocol, participant-level dataset, and statistical code	___25__________
**Appendices**			
Informed consent materials	32	Model consent form and other related documentation given to participants and authorised surrogates	supplementary document 2
Biological specimens	33	Plans for collection, laboratory evaluation, and storage of biological specimens for genetic or molecular analysis in the current trial and for future use in ancillary studies, if applicable	_________NA_____ _

For peer review only - http://bmjopen.bmj.com/site/about/guidelines.xhtml

*It is strongly recommended that this checklist be read in conjunction with the SPIRIT 2013 Explanation & Elaboration for important clarification on the items. Amendments to the protocol should be tracked and dated. The SPIRIT checklist is copyrighted by the SPIRIT Group under the Creative Commons "Attribution-NonCommercial-NoDerivs 3.0 Unported" license.

For peer review only

BMJ Open

Complex interventions to implement a diabetic retinopathy care pathway in the public health system in Kerala: the Nayanamritham study protocol.

Journal:	*BMJ Open*
Manuscript ID	bmjopen-2020-040577.R5
Article Type:	Protocol
Date Submitted by the Author:	15-May-2021
Complete List of Authors:	Sivaprasad, Sobha; UCL, NIHR Moorfields Biomedical Research Centre; Moorfields Eye Hospital NHS Foundation Trust, Medical Retina Department<br>Netuveli, Gopalakrishnan; University of East London - Duncan House Campus, Institute of Connected Communities<br>Wittenberg, Raphael; Oxford University, Nuffield Department of Primary Health Care Sciences<br>Khobragade, Rajan; Government of Kerala, Directorate of Health<br>Sadanandan, Rajeev; Government of Kerala, Directorate of Health<br>Gopal, Bipin; Government Medical College Thiruvananthapuram, Non-Communicable Diseases Department, Directorate of Health Services<br>Premnazir, Lakshmi; Government Medical College Thiruvananthapuram, Directorate of Health Services<br>Conroy, Dolores; UCL, Department of Vision Sciences, Institute of Ophthalmology<br>Srinath, Jyotsna; University of East London - Duncan House Campus, Department of connected communities<br>Ramakrishnan, Radha; UCL, Department of Vision Sciences, Institute of Ophthalmology<br>George, Simon; Government Medical College Thiruvananthapuram, Regional Institute of Ophthalmology<br>Sahasranamam, Vasudeva Iyer ; Government Medical College Thiruvananthapuram, Ophthalmology Department, Regional Institute of Ophthalmology
**Primary Subject Heading**:	Ophthalmology
Secondary Subject Heading:	Public health, Diabetes and endocrinology
Keywords:	Diabetic retinopathy < DIABETES & ENDOCRINOLOGY, General diabetes < DIABETES & ENDOCRINOLOGY, Protocols & guidelines < HEALTH SERVICES ADMINISTRATION & MANAGEMENT

For peer review only - http://bmjopen.bmj.com/site/about/guidelines.xhtml

I, the Submitting Author has the right to grant and does grant on behalf of all authors of the Work (as defined in the below author licence), an exclusive licence and/or a non-exclusive licence for contributions from authors who are: i) UK Crown employees; ii) where BMJ has agreed a CC-BY licence shall apply, and/or iii) in accordance with the terms applicable for US Federal Government officers or employees acting as part of their official duties; on a worldwide, perpetual, irrevocable, royalty-free basis to BMJ Publishing Group Ltd ("BMJ") its licensees and where the relevant Journal is co-owned by BMJ to the co-owners of the Journal, to publish the Work in this journal and any other BMJ products and to exploit all rights, as set out in our licence.

The Submitting Author accepts and understands that any supply made under these terms is made by BMJ to the Submitting Author unless you are acting as an employee on behalf of your employer or a postgraduate student of an affiliated institution which is paying any applicable article publishing charge ("APC") for Open Access articles. Where the Submitting Author wishes to make the Work available on an Open Access basis (and intends to pay the relevant APC), the terms of reuse of such Open Access shall be governed by a Creative Commons licence – details of these licences and which Creative Commons licence will apply to this Work are set out in our licence referred to above.

Other than as permitted in any relevant BMJ Author's Self Archiving Policies, I confirm this Work has not been accepted for publication elsewhere, is not being considered for publication elsewhere and does not duplicate material already published. I confirm all authors consent to publication of this Work and authorise the granting of this licence.

For peer review only

Complex interventions to implement a diabetic retinopathy care pathway in the public health system in Kerala: the Nayanamritham study protocol.

Sobha Sivaprasad[1, 2], Gopalakrishnan Netuveli[3], Raphael Wittenberg[4], Rajan Khobragade[5], Rajeev Sadanandan[5], Bipin Gopal[6], Lakshmi Premnazir[6], Dolores Conroy[2], Jyotsna Srinath[3], Radha Ramakrishnan[2], Simon George[7], Vasudeva Iyer Sahasranamam[7] for the Nayanamritham Project Collaborators.

1. Medical Retina Department, NIHR Biomedical Research Centre, Moorfields Eye Hospital NHS Foundation Trust and University College London, London, UK
2. Department of Vision Sciences, UCL Institute of Ophthalmology, London, UK
3. Institute of Connected Communities, University of East London, UK
4. Nuffield Department of Primary Health Care Sciences, University of Oxford, Oxford, UK
5. Directorate of Health, Government of Kerala, India
6. Non-Communicable Diseases Department, Directorate of Health Services, Kerala, India
7. Ophthalmology Department, Regional Institute of Ophthalmology, Thiruvananthapuram, Kerala, India

Word count: 3696

Figures: 2

Table: 3

References: 33

Abstract: 295

Corresponding author

Professor Sobha Sivaprasad
NIHR Moorfields Biomedical Research Centre
Moorfields Eye Hospital NHS Foundation Trust,
162 City Road,
London EC1V 2PD, UK
Tel: 020 7253 3411
Email: senswathi@aol.com

Keywords: diabetic retinopathy, implementation study, Kerala, laser photocoagulation, diabetes, screening, visual impairment, cost-effectiveness.

Trial Registration Number: ISRCTN28942696

Protocol Version: 1.0 dated 13-03-2019

For peer review only

For peer review only - http://bmjopen.bmj.com/site/about/guidelines.xhtml

Abstract

Introduction: Using a type 2 hybrid effectiveness-implementation design, we aim to pilot a diabetic retinopathy (DR) care pathway in the public health system in Kerala to understand how it can be scaled up to and sustained in the whole state.

Methods and analysis: Currently, there is no systematic DR screening programme in Kerala. Our intervention is a teleophthalmology pathway for people with diabetes in the non-communicable disease registers in 16 family health centres. The planned implementation strategy of the pathway will be developed based on the discrete Expert Recommendations for Implementing Change (ERIC) taxonomy. We will utilise both quantitative data from a cross-sectional study and qualitative data obtained from structured interviews, surveys and group discussions with stakeholders to report the effectiveness of the DR care pathway and evaluation of the implementation strategy. We will use logistic regression models to assess crude associations DR and STDR and fractional polynomials to account for the form of continuous covariates to predict uptake of DR screening. The primary effectiveness outcome is the proportion of patients in the non-communicable disease register with diabetes screened for DR over 12 months. Other outcomes include cost-effectiveness, safety, efficiency, patient satisfaction, timeliness and equity. The outcomes of evaluation of the implementation strategies include acceptability, feasibility, adoption, appropriateness, fidelity, penetration, costs and sustainability. Addition of more family health centres during the staggered initial phase of the programme will be considered as a sign of acceptability and feasibility. In the long term, the state-wide adoption of the DR care pathway will be considered as a successful outcome of the Nayanamritham study.

Ethics and Dissemination: The study was approved by Indian Medical Research Council (2018-0551) dated 13/03/2019. Study findings will be disseminated through scientific publications and the report will inform adoption of the DR care pathway by Kerala state in future.

For peer review only - http://bmjopen.bmj.com/site/about/guidelines.xhtml

ARTICLE SUMMARY

- This study will examine the clinical and cost effectiveness of a new diabetic retinopathy care pathway at the patient, clinician and service levels and evaluate the implementation strategy within a resource constrained environment.
- This type 2 hybrid effectiveness-implementation study will use mixed methods as method of evaluation.
- The specific actions in the implementation strategy are based on the Expert Recommendations for Implementing Change (ERIC) taxonomy
- The study outlines the economic evaluation of the costs of the diabetic retinopathy care pathway**.**
- The study is limited by the absence of a comparator due to lack of previous data on the prevalence of diabetic retinopathy in the public health system.

For peer review only

INTRODUCTION

Background

The triad of diabetes, blindness and poverty is an urgent problem that needs an effective response in the Development Assistance Committee-listed countries as these countries are home to 80% of people with diabetes. [1] Diabetic retinopathy (DR) is one of the most common complications of diabetes. [2] Sight-threatening diabetic retinopathy (STDR) is a common cause of blindness in working-age people and unless this condition is managed early, it has considerable impact on the quality of life and productivity of the person and their family, as well as substantial financial costs to health systems. [3] In its early stages, STDR may be asymptomatic. Therefore, DR screening programmes are essential to identify STDR to enable timely treatment. [4] In DR screening programmes in high income countries, people with diabetes are systematically screened using office-based retinal cameras and the retinal images are graded according to severity of DR by dedicated accredited screeners and graders. Patients with STDR are identified and referred to ophthalmologists for timely treatment with laser and/or intravitreal injections of anti-vascular endothelial growth factor. [4] In order to successfully reduce the risk of blindness due to DR in low-middle income countries (LMICs), without a well-developed primary care infrastructure, the pathway needs to begin with systematically screening for diabetes, educating the public and healthcare professionals on early detection[5] and timely treatment of STDR and the need to optimally treat the risk factors for DR such as hyperglycaemia, hypertension and hyperlipidemia. [2] Moreover, DR screening and treatment are challenging due to the required technology and technical expertise needed to grade retinal images and deliver costly treatment options, adding to the cost and complexity of the required interventions.

Diabetes in Kerala

Kerala is the most advanced state in India in terms of literacy, health, social uplift and demographic transition.[6] This has been accompanied by high prevalence of diabetes. A recent report from Kerala suggests that one in five of the Kerala adult population may have diabetes.[7] The Government of Kerala launched the Aardram Mission in 2017 to transform and gear up the State's public health care system to achieve the Sustainable Development Goals (SDGs) in phases with short term goals on building infrastructure and quality care services. The overarching objectives of the Aardram Mission included providing equitable, affordable and quality care to citizens from all socio-economic strata; strengthening the public care system by

For peer review only - http://bmjopen.bmj.com/site/about/guidelines.xhtml

decentralising healthcare from the secondary and tertiary level to primary care-led services and initiating preventive medicine to address the impact of non-communicable diseases, especially hypertension and diabetes. Primary care centres have been converted to family health centres (FHCs) with three doctors and four nurses in each family health centre assigned to provide individual care plans to the allocated population whose records are tracked through a recently established Electronic Health Records (EHR) called eHealth. The transformation of primary care with a focus on non-communicable diseases provided the backdrop to the implementation of a DR care pathway.

The rationale for a complex DR care pathway

The Government of Kerala is pressing forward to achieve universal health coverage and address the sustained development goals (SDG) goals on poverty (SDG 1), health access (SDG 3), education (SDG 4) and gender equality (SDG 5).[8, 9] Therefore, there is an urgent need to tackle the complications of diabetes. Systematic DR screening has been shown to be effective in reducing blindness.[3] However, in high income countries, DR screening is achieved by dedicated services due to the technicalities and expertise required in DR screening and evaluation.[10-12] Introducing an isolated DR care pathway will not be sufficient to address the challenge in LMICs as these countries need to initiate holistic screening service for diabetes and all its complications simultaneously and embed DR screening within the primary care.

Rationale for an Implementation Strategy

Each aspect of the DR screening and care pathway has to be adapted to local needs and resources, requiring a locally appropriate implementation strategy. For example, mydriasis (pupil dilatation) is compulsory in some screening programmes but not in others and this process requires local approval by stakeholders.[10-12] In Kerala, mydriasis needs to be approved by the health department. Other issues faced by low and middle income countries are that the number of undiagnosed diabetes cases is high,[13] the non-availability of a state-wide diabetes register with recall facilities, the lack of resources for standardised non-portable retinal cameras, limited capacity of staff in primary care to screen for DR, and that the number of people with diabetes is larger than the capacity of the services provided by the secondary care hospitals.[14-16] Research capacity and capability are in their infancy in LMICs and implementing change in a busy environment when demands on staff are great and resources are limited is challenging.[15, 16] In addition, as cataract is more prevalent in low and middle income countries,[17] the proportion of ungradable retinal images is higher compared to developed countries.[17-19]

For peer review only - http://bmjopen.bmj.com/site/about/guidelines.xhtml

Therefore, we need to evaluate the implementation strategy using both qualitative and quantitative methods. [20-22]

Aims and Objectives

The Nayanamritham study is facilitated by a UK-Government of Kerala partnership as a part of the ORNATE-India project funded by Global Challenges Research Fund (GCRF) and UK Research and Innovation (UKRI). We aim to introduce a DR care pathway that spans primary, secondary and tertiary care in the public health system in Kerala in a pilot study in Thiruvananthapuram. The proposed study will (1) examine the clinical and cost effectiveness of the DR care pathway at the patient, clinician and service levels and (2) evaluate the implementation strategy of the pathway.

METHODS

Design: We chose a type 2 hybrid effectiveness-implementation design to evaluate the effectiveness of the clinical interventions and the implementation strategies. [20-23] Mixed methods will be used as the method of evaluation. DR care pathway was developed by the Kerala Health Secretary, non-communicable diseases lead, Health and Medical Education service providers, technical and EHR teams, local authorities and the GCRF/UKRI- funded co-applicants from the UK. The DR care pathway will be set-up in a staggered approach with 5 family health centres initiated in the first three months and the remaining 11 centres will be added based on acceptance of all stakeholders and adapting and training phase for 12 months from 15-03-2018. A further 12-15 months will be allocated to recruit consenting patients to a cross-sectional study to gather quantitative data for the effectiveness outcomes. A minimal dataset from this study will also be entered into EHR to enable future screening for the consented patients. Qualitative data collection from interviews of staff, patients and focus groups at baseline and end of study and all field notes gathered during the study will be utilised to inform evaluation of the implementation strategy. [20-22]

Target Population

People with diabetes registered in the non-communicable diseases register at 16 family health centres in Thiruvananthapuram district will be invited to be screened for complications of diabetes including screening for DR. As the non-communicable diseases register is likely to show an increase in newly diagnosed diabetes as a result of training the accredited social health activists (ASHAs), in diabetes and DR, we will evaluate the effectiveness of the complex

For peer review only - http://bmjopen.bmj.com/site/about/guidelines.xhtml

interventions delivered at each of the 16 family health centres for this study. The implementation of the pathway will take into account all patients in the non-communicable diseases register at the start of the cross-sectional study.

Setting

Target sites for implementation of DR screening and treatment in Thiruvananthapuram will include 16 family health centres for DR screening which represents the primary care centres where patients are screened for all complications of diabetes. The retinal images, captured by the trained, resident nursing staff will be sent to a newly developed reading centre at the Regional Institute of Ophthalmology, a tertiary care centre, where newly accredited graders will grade the images. Patients with screen-positive images will be referred to three secondary care hospitals (district hospitals). Severe cases that require complex interventions will be referred to the tertiary care centre, the tertiary for specialist management of DR.

Description of the standard of care

Currently, patients with diabetes are not systematically screened for DR in the public health system. Most patients present to the tertiary centre voluntarily either because of increased awareness of complications of diabetes or due to visual impairment. Therefore, the current standard of care will be captured as the number of patients presenting to the tertiary centre for an eye consultation for DR over a period of time as there is no baseline data in the primary and secondary care. This cross-sectional survey of the patients presenting at the family health centres will provide information on the uptake of screening of people registered in the non-communicable diseases register at the start of the study. The prevalence of DR and STDR of the screened population will be estimated from the numbers screened in all the non-communicable diseases registers during this period.

Evidence-based clinical intervention:

The new DR care pathway is the intervention and is shown in Figure 1. The pathway will span primary, secondary and tertiary care. The components of the pathway are:

1. DR screening of patients with diabetes registered in the non-communicable diseases register at family health centres (primary care). The retinal images will be graded remotely at the reading centre at the tertiary centre.
2. Prompt referral for timely treatment of STDR to secondary care and tertiary centres depending on the severity of the DR.

For peer review only

3. Treatment of patients with sight threatening DR (at secondary and tertiary care).

Implementation strategies

The implementation strategies of the new DR care pathway are categorised as shown in Table 1 into plan, finance, education, infrastructure, quality improvement and policy contexts. These categories are developed based on the discrete Expert Recommendations for Implementing Change (ERIC) taxonomy.[20, 21] The logic model is shown in Figure 2.

Table 1: Implementation strategies

Categories of Implementation Strategies[21]	Discrete Implementation Strategy[20]	Specific actions
Plan	Conduct local needs assessment	The study team will conduct a situational analysis of data from self-referred patients.
	Assess for readiness and identify barriers and facilitators	Assess barriers that may impede implementation of the DR care pathway, and strengths that can be used in the implementation effort by interviewing key stakeholders i.e. family health centres staff, ophthalmologists in secondary and tertiary care, health service employees and patients.
	Develop a formal implementation blueprint	The blueprint will be used to guide the implementation of the whole DR care pathway and will be updated with time.
	Tailor strategies	Regular collaborative meetings will be held to tailor the implementation strategies to address barriers and leverage facilitators that are identified during various steps in the implementation.
	Stage implementation scale up and integration	The implementation will be staggered with five family health centres starting as pilots to enable small changes before gradually moving to other 11 centres. Addition of more centres during the staggered phase will be considered as sign of acceptability and feasibility.<br>Ensure integration of the DR care pathway into EHR. In the long-term, the adoption of the DR care pathway across the state of Kerala will be considered as success.
	Build a coalition	Cultivate relationships with staff at family health centres and secondary care centres, data entry operators, EHR team, project

For peer review only

For peer review only - http://bmjopen.bmj.com/site/about/guidelines.xhtml

		manager, and the Government of Kerala health service departmental staff in the implementation effort and create a learning system to incorporate practitioner feedback.
	Develop academic partnerships	Partner with UK academic institutions for the purposes of shared training and bringing research skills to the implementation of the pathway.
	Recruit, designate, and train for leadership	A project manager will be recruited to manage the programme under the supervision of the non-communicable diseases lead. The Health Secretary will conduct periodic meetings with the project team to assess performance.
	Obtain formal commitments	A collaborative agreement is in place between Government of Kerala and Moorfields Eye Hospital, London, UK outlining their roles and responsibilities in the implementation of the DR care pathway.
	Prepare patients to be active participants	Community level health workers will prepare patients to be active in their care, to ask questions, and specifically to inquire about care guidelines, the evidence behind clinical decisions, or about available evidence-supported treatments. Each patient will provide written consent to be screened for DR in this project as well as participate in interviews after verbal consent.
	Inform local opinion leaders	The Health Minister of the Kerala state and members of the local government will be briefed on the project and encouraged to be champions of the project to enable scale up to all family health centres in the state.
Finance	Access new funding	Use GCRF/UKRI funding to purchase smartphone retinal cameras and retinal lasers, develop EHR, train staff at family health centres and secondary care, employ project manager, data entry operators and personnel to conduct interviews, create study database linked to hand-held applications for data entry operators. State and local government funds will be sourced for scale up.
	Incentivisation	ASHAs will be incentivised to accompany participants with diabetes who are referred to secondary care for treatment.
Education	Develop educational materials	Develop DR training modules and retinal image capture modules for family health centres staff

For peer review only - http://bmjopen.bmj.com/site/about/guidelines.xhtml

		Develop laser training material to train ophthalmologists.
	Distribute educational materials	Distribute educational materials (including guidelines, manuals, and toolkits) in person, by mail, and/or electronically. Multiple training sessions are planned at each family health centres. The camera manufacturers will be invited to provide hands-on training on capturing good quality retinal images. Training on laser surgery will also be provided to the ophthalmologists at secondary care.
	Conduct educational meetings	Hold meetings targeting different stakeholder groups (ophthalmologists, family health centres staff, ASHAs, patients). Three educational meetings are planned by the UK team to meet with the providers in the family health centres settings to share knowledge and educate providers about the DR care pathway and its integration into their clinical practice.
	Achieve accreditation for graders of retinal images	Train and certify the optometrists in the Reading Centre in the tertiary centre to obtain accreditation as graders using the online international Test and Training for retinal graders.
	Conduct ongoing training	Nurses and doctors in the family health centres will receive on-going training on DR, pupil dilatation and capture of retinal images using low-cost smartphone retinal cameras.[24, 25]
	Create a learning collaborative	The Health Secretary of State will foster a collaborative learning environment to improve implementation of the DR care pathway.
	Use train-the-trainer strategies	The UK team will first train a batch of family health centres nurses to capture retinal images and then some will be trained to train the rest of the nurses. The same cascading pathway will apply to laser training.
	Work with educational institutions	Moorfields Eye Hospital staff will provide the necessary guidance and share knowledge on UK diabetic retinopathy screening pathway.
	Make training dynamic	All staff involved will receive continual online or face to face training.
	Increase demand	The ASHAs will be trained to increase public awareness of this pathway in their house-to-house health visits.

For peer review only

	Provide ongoing consultation	The UK team from Moorfields Eye Hospital will provide ongoing consultation to support implementation of DR care pathway.
Infrastructure	Centralize technical assistance	The project manager and the EHR team will provide central assistance focused on implementation issues. The telemedicine project will link the patient's retinal images to EHR and transfer through a secure cloud to the tertiary centre, where the images will be graded for their DR status in a newly developed reading centre, with the results fed back to the family health centres.[26]
	Change physical structure and equipment	Evaluate current configuration of family health centres consulting rooms to allow suitable furniture for cameras and adapt illumination to capture retinal photographs.
	Change record systems	Change records systems to allow better assessment of implementation or clinical outcomes by having data entry operators interview patients and input full data into EHR within the busy family health centres clinics.
	Change service sites	Change the timetable for clinics in secondary care to allow a patients referred from primary care to access treatment.
	Revise professional roles	The non-communicable diseases lead will be delegated to take on the responsibility of implementing this DR care pathway in Thiruvananthapuram aiming for a Kerala state-wide roll out.
Quality Improvement	Develop and implement tools for quality monitoring	Develop, test, and introduce into quality-monitoring systems the right input—the appropriate language for the consent form, protocols on DR screening, grading retinal images, and measures of processes, patient outcomes, and implementation outcomes.
	Develop and organize quality monitoring systems	Develop and organise systems and procedures that monitor clinical processes and/or outcomes for the purpose of quality assurance and improvement.
	Audit and provide feedback	Collect and summarise clinical performance data every month and feedback to family health centres and secondary care staff to monitor, evaluate, and modify pathway for acceptability to patients and staff. A continuous monitoring of retinal image quality and gradability, and the referral pathway will be done to cyclically input change into the DR pathway to improve the quality and integration.

For peer review only - http://bmjopen.bmj.com/site/about/guidelines.xhtml

	Conduct cyclical small tests of change	Staggered set up of family health centres to allow implementation of changes in a cyclical fashion using small tests of change before taking changes system-wide. Tests of change benefit will be measured continually by increase uptake of training, screening, referral and treatment.
	Use data experts	The EHR team will develop an application for the data entry operators to collect data. The UK collaborators will collaborate in the analysis of the data.
	Use data warehousing techniques	Integrate clinical records from this research project into EHR on an on-going basis.
	Capture and share local knowledge	Capture local knowledge from implementation sites on how staff and doctors make something work in their family health centres and then share it with other centres.
	Obtain and use patients feedback	The participating patients will be asked to provide feedback on the implementation effort.
	Promote adaptability	Identify ways the DR care pathway can be tailored to meet local needs and clarify which elements of the pathway must be maintained to preserve fidelity.
	Provide clinical supervision	The Director of the tertiary centre will provide the overall clinical supervision of the whole DR care pathway. He will also delegate ophthalmologists to provide ongoing supervision for the optometrists involved in grading retinal images.
	Provide local technical assistance	Develop and use a system to deliver technical assistance focused on implementation issues using relevant service providers.
	Purposely re-examine the implementation	Monthly monitoring of screened patients at each family health centre, barriers observed and small changes that are required will be implemented to continuously improve the pathway.
	Remind clinicians	Primary care doctors in family health centres will receive monthly newsletter on the numbers recruited across all centres to highlighting successes and providing potential solutions to issues encountered.
Policy contexts	Issue Government of Kerala approval to enable dilatation of pupils of patients under supervision	Ensure doctors at family health centres receive approval for mydriasis in order to deliver the DR care pathway.

For peer review only - http://bmjopen.bmj.com/site/about/guidelines.xhtml

	of family health centres doctors	
	Mandate change	The Health Secretary of State will provide leadership to ensure the prioritisation of the implementation of the DR care pathway.

DR-diabetic retinopathy; ASHAs -accredited social health activists; GCRF/UKRI -Global Challenge Research Fund/UK Research and Innovation; EHR –Electronic health records.

Outcomes

Pre-specified outcomes of the effectiveness of the DR pathway and the evaluation of the implementation strategy are tabulated in table 2 and table 3, respectively.

Table 2: DR pathway- (intervention) related outcomes

Outcomes	Indicators	Source of data
Clinical Effectiveness	– Proportion of people screened for DR in each family health centres over 12 months.<br>– Proportion of referred patients with STDR.<br>– Proportion of ungradable retinal images and percentage of cataract identified from referring these patients.<br>– Risk and complication burden in people screened for DR<br>– Presenting visual acuity of those referred from the DR pathway compared to those who self-referred to secondary care.<br>– Generalisability of the prevalence data compared to house-to-house survey in a neighbouring district.	Data from monthly entries into EHR and study database.<br>–
Safety	Data on complications of the DR pathway	EHR and study database

For peer review only

For peer review only - http://bmjopen.bmj.com/site/about/guidelines.xhtml

Cost-effectiveness	The key outcome is the number of cases of severe visual impairment or blindness due to STDR averted as a result of the new DR care pathway and resultant QALY gain.<br>Estimated QALY gain from laser treatment of STDR and from cataract surgery, using utility values from Rachapelle et al 2013.[27]<br>Assumption that the effect of no screening is as in historic studies that reported the rate at which STDR leads to blindness if left untreated.	Data collected in the study, data from the literature and expert opinion where data could not be collected.
Efficiency	Efficiency is defined as optimal use of the service. For this purpose, we will first estimate the time required to process one patient from the path-process analysis. This estimate will be used to determine the number of patients who can be seen on a day. We will then calculate (1-number of patients screened)/number of patients that can be screened). proportion can be used in a p-type control chart.[28]	Capture efficiencies in structures, resources and processes across the DR care pathway from staff interviews.<br><br>Path process mining of patients screened in family health centres analysis will be based on detailed time-event logs of approximately 70 patients from the 16 FHCs
Patient satisfaction	Patients willingness to be screened and their satisfaction with the information provided to them and the care they received	Patient interviews and telephone survey of patients referred to secondary care for treatment
Timeliness	Path process mapping at family health centres to understand delays in pathway Reasons for non-attendance for those referred to secondary care for treatment of STDR	Path process mapping at family health centres to understand delays in DR care pathway<br><br>Patient survey and secondary care records
Equity	Age and gender-based prevalence of DR and STDR will be reported on consented patients from all family health centres. Reasons of non-referral of patients that require referrals will be recorded. Numbers denied screening despite willingness to be screened will also be reported.	Data from EHR and study database.

For peer review only - http://bmjopen.bmj.com/site/about/guidelines.xhtml

DR -diabetic retinopathy; STDR-sight threatening diabetic retinopathy; QALY- quality-adjusted life year

Table 3: Outcomes of the evaluation of the implementation strategy

**Outcomes**	**Indicators**	**Assessment and data source**
Acceptability	Willingness of each family health centres to be involved in the DR care pathway.	**Assessment:** Readiness to implement the DR care pathway in the 16 family health centres and any reasons for delay.<br><br>*Data source:* Clinical performance from family health centres that will include monthly uptake of DR screening at each centre and any reports on barriers causing delays in implementation provided by the project manager.
	Staff willingness to train	**Assessment:** Proportions of trained ASHAs, nurses and doctors per family health centres, increase in doctors trained in laser surgery in secondary care, proportions willing to be included in train the trainer programme. The denominator will be total numbers of each personnel available to be trained on the date of implementation.<br><br>*Data sources:* Accreditation and certification records; Structured interviews of at least 5 nurses and 5 primary care doctors from 16 family health centres, 3-5 ophthalmologists from secondary and tertiary care, 2 data entry operators and 5 ASHAs and 1 health service administrator to understand challenges for acceptance.
	Patients acceptability of the pathway	**Assessment:** Screening attendances per family health centres over 12 months.<br><br>*Data sources:* Clinical performance data and a telephone questionnaire survey for screened and referred patients to evaluate reasons for attendance and non-attendance.
Adoption	Uptake of DR care pathway at primary, secondary and tertiary care<br><br>Uptake of training by staff	**Assessment:** Proportion of staff at primary, secondary and tertiary care willing to integrate DR care pathway in their workload.<br><br>*Data source:* family health centres data and staff interviews

For peer review only

	Uptake of screening programme by patients	**Assessment:** Demand training sessions; Attendance rates at training sessions<br><br>*Data source:* number of training sessions and attendance registers at training sessions.<br><br>**Assessment:** Increase in proportions of patient screened over 12 months.<br><br>*Data source:* EHR and study database.
	Adoption of DR care pathway by the Government of Kerala for state-wide implementation.	**Assessment:** A policy paper by the Government of Kerala for state-wide adoption of the DR care pathway as part of diabetes care for all patients.<br><br>*Data source:* A detailed scale-up plan will be developed for each district based on the clinical and cost-effectiveness data from this study to inform policy.
Appropriateness	Appropriateness of family health centres for DR screening<br><br>Appropriateness of the complex DR care pathway across primary and secondary care	**Assessment:** Qualitative data on barriers and facilitators of the delivery of screening and treatment in secondary care.<br><br>*Data sources:* interview of staff at family health centres and secondary care, pPatient telephone survey on pathway from FHCs to secondary care
	Appropriateness of referrals to secondary care	**Assessment:** False positive referrals from data collection at secondary care.<br><br>*Data sources:* study database; data from patient telephone survey
Cost	Costs to the Government of Kerala and societal costs of the DR screening programme and subsequent treatment	**Assessment:** Costs of the DR screening and treatment costs in total and per person, including the costs of staff training and cameras (suitably annuitized), staff costs and travel costs of patients.<br><br>*Data sources:* Data collected in the study baseline and data from other services in India and from the literature.
Feasibility	Screening rate per family health centres Barriers to monthly screening uptake	**Assessment:** Monthly increase in numbers of FHCs added to the programme

For peer review only - http://bmjopen.bmj.com/site/about/guidelines.xhtml

For peer review only

		Data source:Interview and monthly report from project manager on facilitators and barriers on each part of the pathway
Fidelity	Degree of the DR care pathway implementation<br><br>Validation of the DR grading within the telemedicine pathway<br><br>Delivery of training and the impact on the DR care pathway	**Assessment:** Fidelity evaluation in 16 family health centres using a path-process analysis of the screening to identify the common screening pathway across centres .<br><br>*Data source*: The path-process analysis will be based on detailed time-event logs of 70 patients from the 16 family health centres; a telephone follow-up of referred patients to evaluate uptake of referrals.<br>**Assessment:** Validation of the DR grading will be assessed by the agreement of the DR grade reported by the optometrists at the reading centre in the tertiary centre and those reported by the ophthalmologists in the secondary care centres.<br><br>*Data sources*: DR grading of referred patients obtained from the reading centre versus secondary care.<br><br>**Assessment:** Increase in numbers of trained staff per month.<br><br>*Data sources:* Staff interviews, structured observations and review of facility records. The proportions of doctors trained on laser delivery; data quality on the database; Increase in non-communicable diseases registration following start of programme due to training of ASHA workers on diabetes and DR; Increase in numbers of patients treated for STDR following training.
Penetration	Increase patient awareness of diabetes and DR and uptake of the DR screening pathway in patients with diabetes	**Assessment**: Increase uptake of DR screening by patients registered in non-communicable diseases registers.<br><br>*Data sources*: from non-communicable diseases register and EHR
Sustainability	Policy makers to prepare policy on up-scale and sustainability plan for DR pathway	**Assessment:** Availability of a policy paper from Government of Kerala on state-wide scale up of the DR care pathway. This will be considered a success of the programme.<br><br>*Data sources*: Scale up plan for other districts including plan to purchase retinal cameras and

For peer review only - http://bmjopen.bmj.com/site/about/guidelines.xhtml

	Integration of DR pathway in weekly activities in FHCs	acceptance of guidelines and training material for state-wide roll out.<br><br>**Assessment:** Integration of DR pathway in EHR<br><br>*Data source:* EHR records of DR screening as part of non-communicable diseases care pathway.

DR-diabetic retinopathy; ASHA -accredited social health activists, EHR- electronic health records

Data collection

Quantitative data

Effectiveness outcomes will originate from the data sources shown in table 2. Data collected by nurses or data operators from the EHR include age, gender, duration of diabetes, use of insulin, parental history of diabetes, other complications of diabetes including diabetic kidney disease, cardiovascular complications, and diabetic foot, random blood sugar results, urine dipstick test for albuminuria and blood pressure record. Other study-specific data collected by nurses or data operators on the day of screening include education status, occupation and income categories, and previous history of DR, cataract surgery or any other ocular history. In addition, they will measure body mass index, waist circumference and complete a lifestyle questionnaire on smoking, diet, physical activity, EQ-5D vision bolt-on[29]. The EQ-5D vision bolt-on will be used to calculate the quality adjusted life-years and utility value for economic analysis. EQ-5D alone does not capture visual acuity deficits.[30] The EQ-5D vision bolt-on asks patients to rate their health across 6 dimensions: mobility, self-care, usual activities, pain/discomfort, anxiety/depression and vision. Each dimension is scored in 5 levels: no problems, slight problems, moderate problems, severe problems, and extreme problems. A recent study provided utility values based on EQ-5D vision bolt-on. The mapping was done in a clinical trial cohort with macular oedema in central retinal vein occlusion. [31]

In the reading centre, the data collection will include the grade of retinopathy in both eyes, presence of cataract and gradability of the retinal images. Data collected on referred patients will include numbers with ungradable images due to cataract, treatment options offered for DR and review appointment.

For peer review only

For peer review only - http://bmjopen.bmj.com/site/about/guidelines.xhtml

Qualitative data collection and analysis

Data sources used for evaluation of the implementation strategy are shown in table 3. These will include data from a structured interview of a maximum variable sample that will reflect the context (primary, secondary and tertiary care), and the functions and skill levels of the staff (e.g. nurses, doctors etc.). Based on pragmatic considerations, at least 5 nurses and 5 primary care doctors from 16 FHCs, 3-5 ophthalmologists from secondary and tertiary care, 2 data entry operators and 5 ASHAs and 1 health service administrator should be included to get the maximum variability. Verbal consent will be obtained from these health professionals. The interviews will be conducted by an independent member from the GCRF/UKRI-funded team, who is not involved in this study, in the local language within the premises of the healthcare provider. The focus group will consist of groups of patients and staff within one family health centres. In addition, to the voice-recording of the interviews and focus group, interviewers will write field notes to describe the interview situation. The interviews and focus group content will provide the basis for the data analysis, which will be based on a descriptive phenomenological approach without data or opinion interpretation and will include transcription, condensation, coding and categorisation using qualitative analysis tools. We will use the field notes collected during the interviews to inform the understanding of the phenomenon studied.

A survey of all referred patients will be done using a structured questionnaire to evaluate their satisfaction and their perception of the barriers and facilitators. All qualitative data will be coded using NVivo and analysed using descriptive phenomenological approach following the strategy.[32] We will transcribe the interview data, identify statements or phrases, create formulated meanings or meaning units, aggregate formulated meanings and incorporate the result into descriptions.

Data Monitoring

Data will be coded before entry into the study database by the clinical teams. Only anonymised extracted data from EHR by the nurses or data entry operators will be used for analysis. Data quality will be monitored by the study project manager. Anonymised data will be checked for range checks and data quality at University of East London by the study statistics team. The ORNATE India International Advisory Board will have an overview of the conduct of the project and the Executive Group consisting of the UK-India collaborators will monitor the conduct of the study.

For peer review only - http://bmjopen.bmj.com/site/about/guidelines.xhtml

Sample size

We have chosen the proportion of patients in the non-communicable diseases register with diabetes screened for DR as our primary outcome variable.

Justification of the choice of primary outcome: This variable captures the effectiveness of our intervention as well as the fidelity of implementation. Other facts that informed our choice of the primary outcome is that we expect a short implementation time of 7 to 9 months during which other outcomes such as number of patients treated may not be a feasible option. Therefore, we have estimated the sample size based on this primary outcome.

Our calculations are complicated by the expected increase in number of people with diabetes in the non-communicable diseases register as public awareness of diabetes and DR increases so the denominator of numbers screened will be the number of people registered in the non-communicable diseases in each family health centres at the start of the cross-sectional study. There is no data on the baseline proportion of patients that attend the tertiary centre for screening. The prevalence of diabetes is between 10-16% in Thiruvananthapuram of which 8% are estimated to have DR and 3% to have STDR but about 20% may have to be referred. Assuming a finite population of 40,000 patients with diabetes, a simple random sample of 377 patients will be needed. However, we expect large design effects due to clustering patients within family health centres. There is not enough data to calculate the value of this design effect and therefore we assume it to be 3 to give a final sample size of 1,131, which is equivalent to assuming a within-family health centres intracluster correlation coefficient of approximately 0.03 (n=16 family health centres) with negligible residual clustering by the intermediate cluster level of ASHAs, where mean cluster size is smaller.

As described by Becker et al in mammography screening,[33] we expect sources of bias in the implementation programme that will likely influence the sample size calculation. Unscreened DR cases that already exist in the community may contribute to an over-estimation of the effect of screening. We expect lead-time bias because of our short implementation period and so some DR in the community may be missed. A comparison of numbers with DR in family health centres with longer implementation period with those of shorter period may adjust for this bias. We intend to provide a descriptive analysis as well as a temporal comparison using time series analysis.

Statistical Analyses

We will create a complete case dataset of the cross-sectional survey for use in the analyses of effectiveness and assess the potential impact of missing data using sensitivity analysis

For peer review only

For peer review only - http://bmjopen.bmj.com/site/about/guidelines.xhtml

incorporating a range of optimistic and pessimistic scenarios for the impact of missing data. We will model two outcomes that we hypothesised would be influenced by the intervention: (1) uptake of DR screening (primary outcome) and (2) numbers of patients referred for STDR as a result of screening. We will use bivariate logistic regression models to assess crude associations between sociodemographic factors such as age, gender, education, income, and living arrangements, and other clinical data with DR and STDR and use fractional polynomials to account for the form of continuous covariates to predict uptake of DR screening. Factors associated with a $P<0.25$ will be candidates for inclusion in the multivariable logistic regression models. We will test interactions amongst identified main effects to capture improvement in model prediction assessed by reduced residual variance-based statistics. To account for the staggered entry into the study, we will add a variable indicating the month of entry of each family health centres into the programme (e.g., 1. if the family health centre join in the first month of the programme, 2. if in the second month and so on). A non-significant coefficient for this variable will suggest the staggered approach had no effect and a significant coefficient will allow the effect to be quantified for each family health centre. In our final adjusted model, we will consider associations with a $P<.05$ as statistically significant. Adequacy of model discrimination and calibration will be assessed using Receiver Operating Characteristic (ROC) curves. The validation of the telemedicine will be reported as the agreement (kappa statistics) between screen positive patients graded by the graders at the reading centre versus the DR grade as recorded by the ophthalmologists in secondary care centres. A kappa coefficient of 0.6 or higher was pre-specified to indicate validity.

Patient and public involvement (PPI)

A patient and public group will be involved in plans to disseminate the study results and provide their input on the scale-up of this DR care pathway, should the implementation in Thiruvananthapuram be deemed successful by the Government of Kerala.

Ethics and dissemination

The study was approved by Indian Medical Research Council (2018-0551). The study complies with the guidelines of the Declaration of Helsinki. Results of this research are expected to be disseminated through stakeholder reports and via scientific forums, specifically peer-reviewed publications and conference presentations. All participants will give written informed consent prior to entry to the study by the family health centres nurses and will be made aware that participation is strictly voluntary.

For peer review only

For peer review only - http://bmjopen.bmj.com/site/about/guidelines.xhtml

Discussion

At the conclusion of this study, we hope to assess the effectiveness and implementation outcomes of a complex DR care pathway integrating care at primary, secondary and tertiary care, covering a proportion of the diabetic population in Thiruvananthapuram. The study will set the scene for a policy for State-wide Screening and Treatment Pathway for Diabetes. Early identification of STDR and other complications due to this holistic approach and timely treatment, are expected to have a positive impact on rates of blindness, chronic kidney disease, cardiovascular complications and thereby improve health, reduce multi-morbidity and mortality. A DR pathway that straddles primary, secondary and tertiary levels of care, leveraging technology may have advantages of cost effectiveness and ease of implementation in low and middle income countries compared to the current practice of detection and management of self-reported cases in tertiary centres.

This study outlines how the effectiveness of a DR care pathway and its implementation will be evaluated. [21, 22] This study is timely given the increasing numbers of people with diabetes and pressure on finances available for healthcare, necessitating task shifting to enable better coverage of the population.. The study will also examine the organisation functions such as structure, resources and processes that would contribute to better outcomes. We will also examine whether this additional task is feasible given the current skill levels and workload of family health centres and secondary hospitals. The study will reveal whether current health seeking behaviour of patients will support screening, especially when an invasive procedure is requested when there is no obvious impact on quality of life of the patient. By addressing a key gap in knowledge due to lack of research in this area, we will be able to decipher the barriers and facilitators that influence the successful implementation of such a programme in the public system in India. The results of this study may inform the adoption of this pathway in other areas in India and globally. However, the complexity and number of implementation strategies, local contextual factors and lack of validated implementation outcomes may limit generalisability of the results and implementation of this pathway elsewhere.

There are limitations, however, to the study design. We do not have baseline data on DR screening in the study location as these screening programmes are non-existent. For this reason, we are examining the effectiveness of the pathway in terms of presenting visual acuity for referable cases as ‘proximal’ outcomes. In addition, when there is no concurrent control

For peer review only - http://bmjopen.bmj.com/site/about/guidelines.xhtml

group, causal inferences are difficult to make and may lead to both measured and unmeasured biases. When examining implementation, logistical issues, variations in health care facilities, socio-economic variation, and quality of health care personnel may likely affect our study. However, the study design avoids the ethical challenges of having a control group with no DR screening. Therefore, the study design is by necessity non-randomised and observational and will rely on newly trained staff members to collect data, which is likely to differ in completeness between the 16 family health centres. We expect an increase in referral rates after implementation of the intervention due to better public awareness, increasing knowledge of ASHAs and improved case ascertainment, at least in some family health centres . We will also examine the impact of cataract in the community and this has not been studied before in any other DR pathway. We have tried to minimise the limitations by the use of robust statistical techniques and the use of various data sources to elicit a greater understanding of how the programme will lead to better health outcomes.

One of the strengths of this study is that quantitative data backed by qualitative data will be collected to strengthen our findings and enable generalisation of our findings. Secondly, we will use robust statistical methods to reduce bias including selection bias and other confounders. Finally, the access to and collaboration with the UK is a key strength of the study, as it facilitates the co-development of the interventions from the outset. Active involvement of policy makers engaged in transformation of primary care to screen for and address complications of non-communicable diseases who value research to generate evidence for policy making and who are prepared to learn from, and adapt the DR care pathway based on implementation experience is a unique feature of this study.. Findings on the mechanisms and contexts that optimise the implementation of this complex multi-faceted intervention using the ERIC taxonomy will be useful to those developing and implementing these programmes in other health systems. The health economic model may highlight the health expenditure required at individual, family and Kerala State level for forecasting and planning health budgets.

Perhaps the most important aspect of the chosen evaluation is that it is built within a simultaneous developing public health strategy on population-based screening of diabetes and hypertension and a recently introduced EHR called eHealth. Conducting research in such an environment is a good example of health policy and systems research.

For peer review only

For peer review only - http://bmjopen.bmj.com/site/about/guidelines.xhtml

Despite the limitations, this study holds promise for providing high-quality data and detailed implementation information on a complex intervention in a resources-limited setting. We hope to contribute to the literature on the implementation and effectiveness of DR screening and treatment in the public health sector in low and middle income countries.

Author Contributorship

Sobha Sivaprasad (S.S), Gopalakrishnan Netuveli (G.N), Raphael Wittenberg (R.W), Rajan Khobragade (R.K), Rajeev Sadanandan (R.S), Bipin Gopal (B.G), Lakshmi Premnazir (L.P), Dolores Conroy (D.C), Jyotsna Srinath (J.S), Radha Ramakrishnan (R.R), Simon George (S.G), Vasudeva Iyer Sahasranamam (V.S)

Conceptualisation, S.S, G.N, R.W, R.S, B.G, S.G, V.S; methodology, S.S, G.N, R.R, R.S, B.G, V.S, S.G, D.C, R.R, R.K; acquisition of data, S.S, G.N, R.W, D.C, R.R, J.S, L.P, B.G; writing—original draft preparation, S.S, D.C, G.N, R.W, L.P, R.R; writing—review and editing, S.S, G.N, R.R, R.S, B.G, V.S, S.G, D.C, R.R, R.K, J.S, L.P; funding acquisition, S.S, G.N, R.W, R.S, on behalf of the Collaborators.

Role of study sponsor and funders, if any, in study design; collection, management, analysis, and interpretation of data; writing of the report; and the decision to submit the report for publication, including whether they will have ultimate authority over any of these activities; None

Role of sponsor: The Director of Regional Institute of Ophthalmology contributed to study design; management, writing of the report; and the decision to submit the report for publication, but have no authority over any of these activities.

Authorship eligibility guidelines will be based on ICJME and there is no intention to use professional writers.

Acknowledgements:
Nayanamritham Project Collaborators: Dr Rajeevan Palpoo and Dr Chitra Raghavan will manage the referred patients in Regional Institute of Ophthalmology; Alwin Aniyankunju and Anju V.Molly will be responsible for grading the retinal images.
Steering Committee: Members of the International Advisory Board from Kerala

Funding statement: This work is part of the ORNATE India project funded by Global Challenges Research Fund and UK Research and Innovation through the Medical Research Council grant number MR/P027881/1

Data Sharing Agreement: The technical appendix, statistical code and dataset will be made available on request after obtaining permission from the Government of Kerala.

For peer review only - http://bmjopen.bmj.com/site/about/guidelines.xhtml

Competing interests statement: None

Sponsor: Dr V Sahasranamam, Director, Regional Institute of Ophthalmology, Thiruvananthapuram, Kerala

References

1. International Diabetes Federation. *IDF Diabetes Atlas*. 9th edition 2019 https://www.diabetesatlas.org/en/
2. Yau JW, Rogers SL, Kawasaki R, et al. Meta-Analysis for Eye Disease (META-EYE) Study Group. Global prevalence and risk factors of diabetic retinopathy. *Diabetes Care* 2012; 35:556–64.
3. IDF Diabetes Atlas: Global estimates of diabetes prevalence for 2017 and projections for 2045. Cho NH, Shaw JE, Karuranga S, Huang Y, da Rocha Fernandes JD, Ohlrogge AW, Malanda B Diabetes Res Clin Pract. 2018; 138():271-281.
4. Scanlon PH. The English National Screening Programme for diabetic retinopathy 2003-2016. *Acta Diabetol* 2017; 54(6):515-25
5. Cavan D, Makaroff L, da Rocha Fernandes J, et al. The Diabetic Retinopathy Barometer Study: Global perspectives on access to and experiences of diabetic retinopathy screening and treatment. *Diabetes Res Clin Pract* 2017; 129:16-24.
6. NITI Ayog (2020), SDG India Index, Baseline Report, 2018, New Delhi [Last accessed on 2021 Jan 17]. Available from: www.niti.gove.in
7. Menon VU, Kumar KV, Gilchrist A, et al. Prevalence of known and undetected diabetes and associated risk factors in central Kerala--ADEPS. *Diabetes Res Clin Pract* 2006; 74:289–94.
8. Sustainable Development Goals. United Nations. [Last accessed on 2020 May12]. Available from: http://www.un.org/sustainabledevelopment/sustainable-developmentgoals/
9. The WHO STEPwise approach to Surveillance of noncommunicable diseases (STEPS). Noncommunicable Diseases and Mental Health. World Health Organization. 20 Avenue Appia, 1211 Geneva 27, Switzerland.
10. Scanlon PH, Aldington SJ, Leal J, et al. Development of a cost-effectiveness model for optimisation of the screening interval in diabetic retinopathy screening. *Health Technol Assess* 2015;19(74):1-116.
11. Leese GP, Morris AD, Olson J. A national retinal screening programme for diabetes in Scotland. *Diabet Med* 2003;20(12):962-4.
12. Lund SH, Aspelund T, Kirby P et al. Individualised risk assessment for diabetic retinopathy and optimisation of screening intervals: a scientific approach to reducing healthcare costs. *Br J Ophthalmol*. 2016;100(5):683-7.

For peer review only - http://bmjopen.bmj.com/site/about/guidelines.xhtml

13. Manne-Goehler J, Geldsetzer P, Agoudavi K et al. Health system performance for people with diabetes in 28 low- and middle-income countries: A cross-sectional study of nationally representative surveys. *PLoS Med*. 2019;16(3):e1002751.

14. Ting DS, Cheung GC, Wong TY. Diabetic retinopathy: global prevalence, major risk factors, screening practices and public health challenges: a review. *Clin Exp Ophthalmol.* 201;44(4):260-77.

15. Hazin R, Barazi MK, Summerfield M. Challenges to establishing nationwide diabetic retinopathy screening programs. *Curr Opin Ophthalmol*. 2011;22(3):174-9.

16. Burgess PI, Msukwa G, Beare NA. Diabetic retinopathy in sub-Saharan Africa: meeting the challenges of an emerging epidemic. *BMC Med*. 2013;11:157.

17. Ramke J, Petkovic J, Welch V et al. Interventions to improve access to cataract surgical services and their impact on equity in low- and middle-income countries. *Cochrane Database Syst Rev*. 2017;11(11):CD011307.

18. Rani PK, Bhattarai Y, Sheeladevi S et al. Analysis of yield of retinal imaging in a rural diabetes eye care model. *Indian J Ophthalmol*. 2018 Feb;66(2):233-237.

19. Scanlon PH, Foy C, Malhotra R, Aldington SJ. The influence of age, duration of diabetes, cataract, and pupil size on image quality in digital photographic retinal screening. *Diabetes Care*. 2005;28(10):2448-53.

20. Powell BJ, Waltz TJ, Chinman MJ et al. A refined compilation of implementation strategies: results from the Expert Recommendations for Implementing Change (ERIC) project. *Implement Sci*. 2015;10:21.

21. Powell BJ, McMillen JC, Proctor EK, et al. A compilation of strategies for implementing clinical innovations in health and mental health. *Med Care Res Rev*. 2012;69(2):123-157.

22. Smith JD, Li DH, Rafferty MR. The Implementation Research Logic Model: a method for planning, executing, reporting, and synthesizing implementation projects. *Implement Sci*. 2020 Sep 25;15(1):84.

23. Landes SJ, McBain SA, Curran GM. Reprint of: An introduction to effectiveness-implementation hybrid designs. *Psychiatry Res*. 2020 Jan;283:112630.

24. Rajalakshmi R, Subashini R, Anjana RM, et al. Automated diabetic retinopathy detection in smartphone-based fundus photography using artificial intelligence. *Eye* (Lond) 2018;32(6):1138-1144

25. Piyasena MMPN, Murthy GVS, Yip JLY et al. Systematic review and meta-analysis of diagnostic accuracy of detection of any level of diabetic retinopathy using digital retinal imaging. *Syst Rev*. 2018 Nov 7;7(1):182. doi: 10.1186/s13643-018-0846-y. Erratum in: Syst Rev. 2019 Apr 30;8(1):106.

26. Boucher MC, Nguyen MTD, Qian J. Assessment of Training Outcomes of Nurse Readers for Diabetic Retinopathy Telescreening: Validation Study. *JMIR Diabetes*.

For peer review only - http://bmjopen.bmj.com/site/about/guidelines.xhtml

2020;5(2):e17309.

27. Rachapelle S, Legood R, Alavi Y, Lindfield R, Sharma T, Kuper H, Polack S. The cost-utility of telemedicine to screen for diabetic retinopathy in India. *Ophthalmology*. 2013 Mar;120(3):566-573.

28. NIST/SEMATECH e-Handbook of Statistical Methods. Proportion Control Chart https://www.itl.nist.gov/div898/handbook/pmc/section3/pmc332.htm, Accessed 5/1/2021

29. Finch A.P., Brazier J.E., Mukuria C., Bjorner J.B. An exploratory study on using principal-component analysis and confirmatory factor analysis to identify bolt-on dimensions: the EQ-5D case study. *Value Health.* 2017;**20**(10):1362–1375.

30. Tosh J, Brazier J, Evans P, Longworth L. A review of generic preference-based measures of health-related quality of life in visual disorders. Value Health. 2012 Jan;15(1):118-27.

31. Pennington BM, Hernández-Alava M, Hykin P, et al. Mapping From Visual Acuity to EQ-5D, EQ-5D With Vision Bolt-On, and VFQ-UI in Patients With Macular Edema in the LEAVO Trial. *Value Health*. 2020;23(7):928-935.

32. Colaizzi PF. Psychological research as the phenomenologist views it. In: Valle R, editor. KM, New York: Oxford University Press; 1978.

33. Becker N, Hakama M, Nyström L. Evaluation of effectiveness of quality-assured mammography screening in Germany: sample size considerations and design options. *Eur J Cancer Prev* 2007;16(3):225-31.

Legend: Figure 1: Proposed Diabetic Retinopathy Care Pathway

Figure 2: The Implementation Research Logic Model

For peer review only - http://bmjopen.bmj.com/site/about/guidelines.xhtml

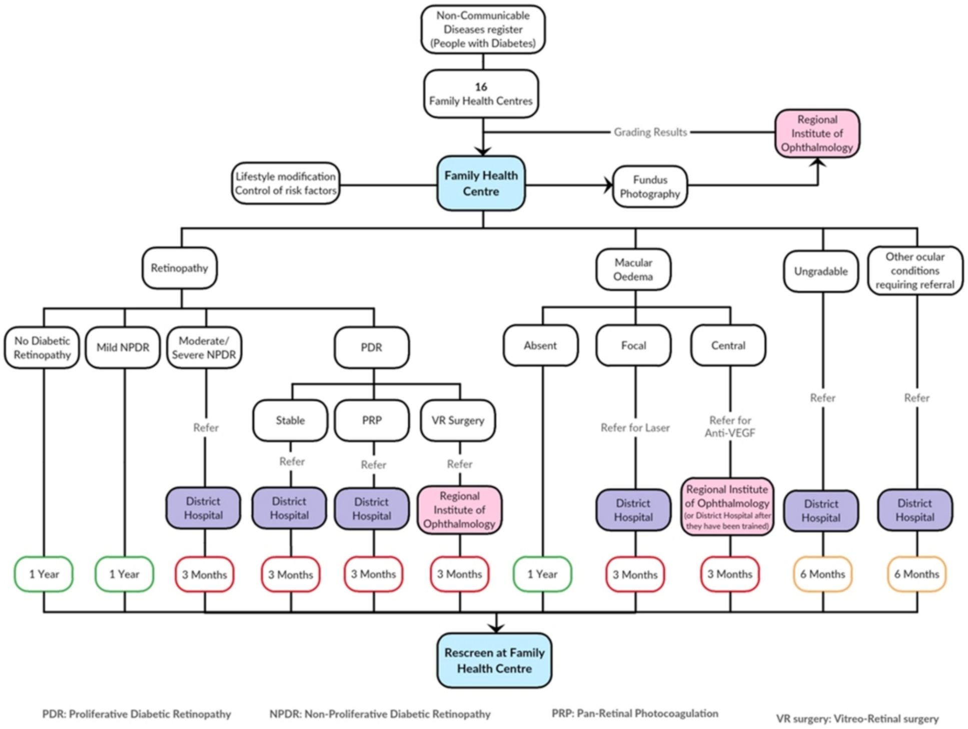

Figure !

186x138mm (300 x 300 DPI)

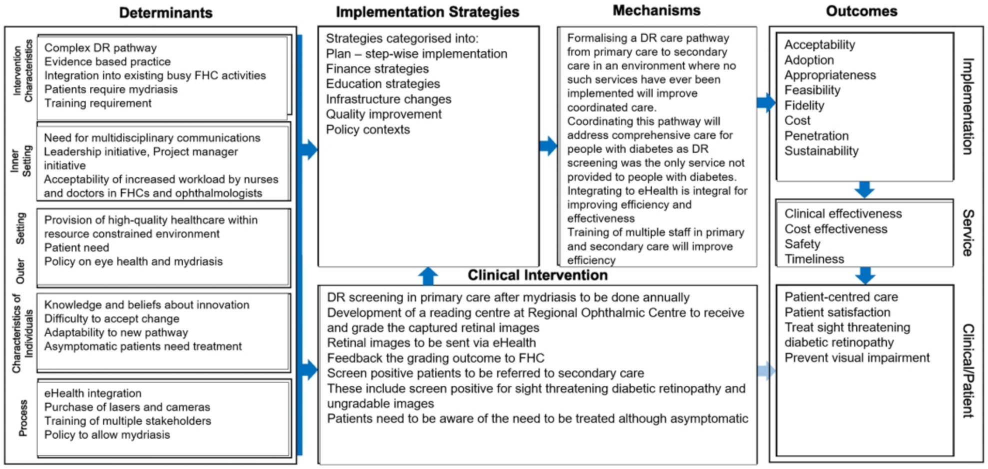

Figure 2 The Implementation Research Logic Model

188x90mm (300 x 300 DPI)

SPIRIT

STANDARD PROTOCOL ITEMS: RECOMMENDATIONS FOR INTERVENTIONAL TRIALS

SPIRIT 2013 Checklist: Recommended items to address in a clinical trial protocol and related documents*

Section/item	Item No	Description	Addressed on page number
**Administrative information**			
Title	1	Descriptive title identifying the study design, population, interventions, and, if applicable, trial acronym	_ 1____________
Trial registration	2a	Trial identifier and registry name. If not yet registered, name of intended registry	__1___________
	2b	All items from the World Health Organization Trial Registration Data Set	__NA__________
Protocol version	3	Date and version identifier	_1_____________
Funding	4	Sources and types of financial, material, and other support	__25_____
Roles and responsibilities	5a	Names, affiliations, and roles of protocol contributors	__24___________
	5b	Name and contact information for the trial sponsor	__25___________
	5c	Role of study sponsor and funders, if any, in study design; collection, management, analysis, and interpretation of data; writing of the report; and the decision to submit the report for publication, including whether they will have ultimate authority over any of these activities	__25___________
	5d	Composition, roles, and responsibilities of the coordinating centre, steering committee, endpoint adjudication committee, data management team, and other individuals or groups overseeing the trial, if applicable (see Item 21a for data monitoring committee)	__19_________

For peer review only

**Introduction**			
Background and rationale	6a	Description of research question and justification for undertaking the trial, including summary of relevant studies (published and unpublished) examining benefits and harms for each intervention	__4_and 5
	6b	Explanation for choice of comparators	__6___________
Objectives	7	Specific objectives or hypotheses	__6__________
Trial design	8	Description of trial design including type of trial (eg, parallel group, crossover, factorial, single group), allocation ratio, and framework (eg, superiority, equivalence, noninferiority, exploratory)	___6__________
**Methods: Participants, interventions, and outcomes**			
Study setting	9	Description of study settings (eg, community clinic, academic hospital) and list of countries where data will be collected. Reference to where list of study sites can be obtained	___7__________
Eligibility criteria	10	Inclusion and exclusion criteria for participants. If applicable, eligibility criteria for study centres and individuals who will perform the interventions (eg, surgeons, psychotherapists)	___7 _________
Interventions	11a	Interventions for each group with sufficient detail to allow replication, including how and when they will be administered	___7__________
	11b	Criteria for discontinuing or modifying allocated interventions for a given trial participant (eg, drug dose change in response to harms, participant request, or improving/worsening disease)	____7-8_______
	11c	Strategies to improve adherence to intervention protocols, and any procedures for monitoring adherence (eg, drug tablet return, laboratory tests)	_____18________
	11d	Relevant concomitant care and interventions that are permitted or prohibited during the trial	_____NA_______ _
Outcomes	12	Primary, secondary, and other outcomes, including the specific measurement variable (eg, systolic blood pressure), analysis metric (eg, change from baseline, final value, time to event), method of aggregation (eg, median, proportion), and time point for each outcome. Explanation of the clinical relevance of chosen efficacy and harm outcomes is strongly recommended	table 2 and 3

For peer review only - http://bmjopen.bmj.com/site/about/guidelines.xhtml

Participant timeline	13	Time schedule of enrolment, interventions (including any run-ins and washouts), assessments, and visits for participants. A schematic diagram is highly recommended (see Figure)	Figure 1
Sample size	14	Estimated number of participants needed to achieve study objectives and how it was determined, including clinical and statistical assumptions supporting any sample size calculations	20
Recruitment	15	Strategies for achieving adequate participant enrolment to reach target sample size	table 1

Methods: Assignment of interventions (for controlled trials)

Allocation:

Sequence generation	16a	Method of generating the allocation sequence (eg, computer-generated random numbers), and list of any factors for stratification. To reduce predictability of a random sequence, details of any planned restriction (eg, blocking) should be provided in a separate document that is unavailable to those who enrol participants or assign interventions	_NA_________
Allocation concealment mechanism	16b	Mechanism of implementing the allocation sequence (eg, central telephone; sequentially numbered, opaque, sealed envelopes), describing any steps to conceal the sequence until interventions are assigned	NA________
Implementation	16c	Who will generate the allocation sequence, who will enrol participants, and who will assign participants to interventions	NA_______
Blinding (masking)	17a	Who will be blinded after assignment to interventions (eg, trial participants, care providers, outcome assessors, data analysts), and how	NA_________
	17b	If blinded, circumstances under which unblinding is permissible, and procedure for revealing a participant's allocated intervention during the trial	NA________

Methods: Data collection, management, and analysis

Data collection methods	18a	Plans for assessment and collection of outcome, baseline, and other trial data, including any related processes to promote data quality (eg, duplicate measurements, training of assessors) and a description of study instruments (eg, questionnaires, laboratory tests) along with their reliability and validity, if known. Reference to where data collection forms can be found, if not in the protocol	_20-21____

	18b	Plans to promote participant retention and complete follow-up, including list of any outcome data to be collected for participants who discontinue or deviate from intervention protocols	____8-12______
Data management	19	Plans for data entry, coding, security, and storage, including any related processes to promote data quality (eg, double data entry; range checks for data values). Reference to where details of data management procedures can be found, if not in the protocol	____17-18__
Statistical methods	20a	Statistical methods for analysing primary and secondary outcomes. Reference to where other details of the statistical analysis plan can be found, if not in the protocol	____ 21_______
	20b	Methods for any additional analyses (eg, subgroup and adjusted analyses)	______21______
	20c	Definition of analysis population relating to protocol non-adherence (eg, as randomised analysis), and any statistical methods to handle missing data (eg, multiple imputation)	____NA_________
**Methods: Monitoring**			
Data monitoring	21a	Composition of data monitoring committee (DMC); summary of its role and reporting structure; statement of whether it is independent from the sponsor and competing interests; and reference to where further details about its charter can be found, if not in the protocol. Alternatively, an explanation of why a DMC is not needed	____19________
	21b	Description of any interim analyses and stopping guidelines, including who will have access to these interim results and make the final decision to terminate the trial	____NA_________
Harms	22	Plans for collecting, assessing, reporting, and managing solicited and spontaneously reported adverse events and other unintended effects of trial interventions or trial conduct	_____NA________
Auditing	23	Frequency and procedures for auditing trial conduct, if any, and whether the process will be independent from investigators and the sponsor	____NA_________
**Ethics and dissemination**			
Research ethics approval	24	Plans for seeking research ethics committee/institutional review board (REC/IRB) approval	______22_____

Protocol amendments	25	Plans for communicating important protocol modifications (eg, changes to eligibility criteria, outcomes, analyses) to relevant parties (eg, investigators, REC/IRBs, trial participants, trial registries, journals, regulators)	___1__________
Consent or assent	26a	Who will obtain informed consent or assent from potential trial participants or authorised surrogates, and how (see Item 32)	____22_________
	26b	Additional consent provisions for collection and use of participant data and biological specimens in ancillary studies, if applicable	____NA________
Confidentiality	27	How personal information about potential and enrolled participants will be collected, shared, and maintained in order to protect confidentiality before, during, and after the trial	_____19
Declaration of interests	28	Financial and other competing interests for principal investigators for the overall trial and each study site	___25__________
Access to data	29	Statement of who will have access to the final trial dataset, and disclosure of contractual agreements that limit such access for investigators	____25_________
Ancillary and post-trial care	30	Provisions, if any, for ancillary and post-trial care, and for compensation to those who suffer harm from trial participation	____NA________ _
Dissemination policy	31a	Plans for investigators and sponsor to communicate trial results to participants, healthcare professionals, the public, and other relevant groups (eg, via publication, reporting in results databases, or other data sharing arrangements), including any publication restrictions	____22_________
	31b	Authorship eligibility guidelines and any intended use of professional writers	____25_________
	31c	Plans, if any, for granting public access to the full protocol, participant-level dataset, and statistical code	___25_________
**Appendices**			
Informed consent materials	32	Model consent form and other related documentation given to participants and authorised surrogates	supplementary document 2
Biological specimens	33	Plans for collection, laboratory evaluation, and storage of biological specimens for genetic or molecular analysis in the current trial and for future use in ancillary studies, if applicable	________NA____ _

For peer review only - http://bmjopen.bmj.com/site/about/guidelines.xhtml

*It is strongly recommended that this checklist be read in conjunction with the SPIRIT 2013 Explanation & Elaboration for important clarification on the items. Amendments to the protocol should be tracked and dated. The SPIRIT checklist is copyrighted by the SPIRIT Group under the Creative Commons "Attribution-NonCommercial-NoDerivs 3.0 Unported" license.

For peer review only
